# Supplementary material for: Point-of-care ultrasound use in austere environments: A scoping review
Source: PLoS One. 2024 Dec 5;19(12):e0312017. doi: 10.1371/journal.pone.0312017 (PMC11620461; doi:10.1371/journal.pone.0312017)
Supplement: S1 Appendix — (DOCX) [file pone.0312017.s002.docx]

Point-of-care ultrasound use in austere environments - searches performed on August 6, 2024:

PubMed: 1121 articles

Search: **(((((((point-of-care ultrasound in austere environments) OR (point-of-care ultrasound in military combat)) OR (point-of-care ultrasound military)) OR (point-of-care ultrasound prehospital)) OR (point-of-care ultrasound in resource-limited settings)) OR (point-of-care ultrasound low and middle income countries)) OR (point-of-care ultrasound in microgravity)) OR (point-of-care ultrasound at high altitude)**

(("point of care systems"[MeSH Terms] OR ("point of care"[All Fields] AND "systems"[All Fields]) OR "point of care systems"[All Fields] OR ("point"[All Fields] AND "care"[All Fields]) OR "point of care"[All Fields]) AND ("diagnostic imaging"[MeSH Subheading] OR ("diagnostic"[All Fields] AND "imaging"[All Fields]) OR "diagnostic imaging"[All Fields] OR "ultrasound"[All Fields] OR "ultrasonography"[MeSH Terms] OR "ultrasonography"[All Fields] OR "ultrasonics"[MeSH Terms] OR "ultrasonics"[All Fields] OR "ultrasounds"[All Fields] OR "ultrasound s"[All Fields]) AND ("resource limited settings"[MeSH Terms] OR ("resource limited"[All Fields] AND "settings"[All Fields]) OR "resource limited settings"[All Fields] OR ("austere"[All Fields] AND "environments"[All Fields]) OR "austere environments"[All Fields])) OR (("point of care systems"[MeSH Terms] OR ("point of care"[All Fields] AND "systems"[All Fields]) OR "point of care systems"[All Fields] OR ("point"[All Fields] AND "care"[All Fields]) OR "point of care"[All Fields]) AND ("diagnostic imaging"[MeSH Subheading] OR ("diagnostic"[All Fields] AND "imaging"[All Fields]) OR "diagnostic imaging"[All Fields] OR "ultrasound"[All Fields] OR "ultrasonography"[MeSH Terms] OR "ultrasonography"[All Fields] OR "ultrasonics"[MeSH Terms] OR "ultrasonics"[All Fields] OR "ultrasounds"[All Fields] OR "ultrasound s"[All Fields]) AND ("militaries"[All Fields] OR "military personnel"[MeSH Terms] OR ("military"[All Fields] AND "personnel"[All Fields]) OR "military personnel"[All Fields] OR "military"[All Fields] OR "military s"[All Fields]) AND ("combat"[All Fields] OR "combatant"[All Fields] OR "combatants"[All Fields] OR "combated"[All Fields] OR "combating"[All Fields] OR "combats"[All Fields] OR "combatted"[All Fields] OR "combatting"[All Fields])) OR (("point of care systems"[MeSH Terms] OR ("point of care"[All Fields] AND "systems"[All Fields]) OR "point of care systems"[All Fields] OR ("point"[All Fields] AND "care"[All Fields]) OR "point of care"[All Fields]) AND ("diagnostic imaging"[MeSH Subheading] OR ("diagnostic"[All Fields] AND "imaging"[All Fields]) OR "diagnostic imaging"[All Fields] OR "ultrasound"[All Fields] OR "ultrasonography"[MeSH Terms] OR "ultrasonography"[All Fields] OR "ultrasonics"[MeSH Terms] OR "ultrasonics"[All Fields] OR "ultrasounds"[All Fields] OR "ultrasound s"[All Fields]) AND ("militaries"[All Fields] OR "military personnel"[MeSH Terms] OR ("military"[All Fields] AND "personnel"[All Fields]) OR "military personnel"[All Fields] OR "military"[All Fields] OR "military s"[All Fields])) OR (("point of care systems"[MeSH Terms] OR ("point of care"[All Fields] AND "systems"[All Fields]) OR "point of care systems"[All Fields] OR ("point"[All Fields] AND "care"[All Fields]) OR "point of care"[All Fields]) AND ("diagnostic imaging"[MeSH Subheading] OR ("diagnostic"[All Fields] AND "imaging"[All Fields]) OR "diagnostic imaging"[All Fields] OR "ultrasound"[All Fields] OR "ultrasonography"[MeSH Terms] OR "ultrasonography"[All Fields] OR "ultrasonics"[MeSH Terms] OR "ultrasonics"[All Fields] OR "ultrasounds"[All Fields] OR "ultrasound s"[All Fields]) AND ("prehospital"[All Fields] OR "prehospitally"[All Fields])) OR (("point of care systems"[MeSH Terms] OR ("point of care"[All Fields] AND "systems"[All Fields]) OR "point of care systems"[All Fields] OR ("point"[All Fields] AND "care"[All Fields]) OR "point of care"[All Fields]) AND ("diagnostic imaging"[MeSH Subheading] OR ("diagnostic"[All Fields] AND "imaging"[All Fields]) OR "diagnostic imaging"[All Fields] OR "ultrasound"[All Fields] OR "ultrasonography"[MeSH Terms] OR "ultrasonography"[All Fields] OR "ultrasonics"[MeSH Terms] OR "ultrasonics"[All Fields] OR "ultrasounds"[All Fields] OR "ultrasound s"[All Fields]) AND ("resource limited settings"[MeSH Terms] OR ("resource limited"[All Fields] AND "settings"[All Fields]) OR "resource limited settings"[All Fields] OR ("resource"[All Fields] AND "limited"[All Fields] AND "settings"[All Fields]) OR "resource limited settings"[All Fields])) OR (("point of care systems"[MeSH Terms] OR ("point of care"[All Fields] AND "systems"[All Fields]) OR "point of care systems"[All Fields] OR ("point"[All Fields] AND "care"[All Fields]) OR "point of care"[All Fields]) AND ("diagnostic imaging"[MeSH Subheading] OR ("diagnostic"[All Fields] AND "imaging"[All Fields]) OR "diagnostic imaging"[All Fields] OR "ultrasound"[All Fields] OR "ultrasonography"[MeSH Terms] OR "ultrasonography"[All Fields] OR "ultrasonics"[MeSH Terms] OR "ultrasonics"[All Fields] OR "ultrasounds"[All Fields] OR "ultrasound s"[All Fields]) AND ("developing countries"[MeSH Terms] OR ("developing"[All Fields] AND "countries"[All Fields]) OR "developing countries"[All Fields] OR ("low"[All Fields] AND "middle"[All Fields] AND "income"[All Fields] AND "countries"[All Fields]) OR "low and middle income countries"[All Fields])) OR (("point of care systems"[MeSH Terms] OR ("point of care"[All Fields] AND "systems"[All Fields]) OR "point of care systems"[All Fields] OR ("point"[All Fields] AND "care"[All Fields]) OR "point of care"[All Fields]) AND ("diagnostic imaging"[MeSH Subheading] OR ("diagnostic"[All Fields] AND "imaging"[All Fields]) OR "diagnostic imaging"[All Fields] OR "ultrasound"[All Fields] OR "ultrasonography"[MeSH Terms] OR "ultrasonography"[All Fields] OR "ultrasonics"[MeSH Terms] OR "ultrasonics"[All Fields] OR "ultrasounds"[All Fields] OR "ultrasound s"[All Fields]) AND ("weightlessness"[MeSH Terms] OR "weightlessness"[All Fields] OR "microgravity"[All Fields])) OR (("point of care systems"[MeSH Terms] OR ("point of care"[All Fields] AND "systems"[All Fields]) OR "point of care systems"[All Fields] OR ("point"[All Fields] AND "care"[All Fields]) OR "point of care"[All Fields]) AND ("diagnostic imaging"[MeSH Subheading] OR ("diagnostic"[All Fields] AND "imaging"[All Fields]) OR "diagnostic imaging"[All Fields] OR "ultrasound"[All Fields] OR "ultrasonography"[MeSH Terms] OR "ultrasonography"[All Fields] OR "ultrasonics"[MeSH Terms] OR "ultrasonics"[All Fields] OR "ultrasounds"[All Fields] OR "ultrasound s"[All Fields]) AND "high"[All Fields] AND ("altitude"[MeSH Terms] OR "altitude"[All Fields] OR "altitudes"[All Fields])) AND English[Language]

Embase: 31

(**'point-of-care ultrasound in austere environments'** OR ((**'point of care'**/exp OR **'point of care'**) AND (**'ultrasound'**/exp OR **ultrasound**) AND **in** AND **austere** AND **environments**) OR **'point-of-care ultrasound in military combat'** OR ((**'point of care'**/exp OR **'point of care'**) AND (**'ultrasound'**/exp OR **ultrasound**) AND **in** AND (**'military'**/exp OR **military**) AND **combat**) OR **'point-of-care ultrasound military'** OR ((**'point of care'**/exp OR **'point of care'**) AND (**'ultrasound'**/exp OR **ultrasound**) AND (**'military'**/exp OR **military**)) OR **'point-of-care ultrasound prehospital'** OR ((**'point of care'**/exp OR **'point of care'**) AND (**'ultrasound'**/exp OR **ultrasound**) AND **prehospital**) OR **'point-of-care ultrasound in resource-limited settings'** OR ((**'point of care'**/exp OR **'point of care'**) AND (**'ultrasound'**/exp OR **ultrasound**) AND **in** AND **'resource limited'** AND **settings**) OR **'point-of-care ultrasound low'** OR ((**'point of care'**/exp OR **'point of care'**) AND (**'ultrasound'**/exp OR **ultrasound**) AND **low**)) AND (**'middle income countries'** OR (**middle** AND (**'income'**/exp OR **income**) AND **countries**)) OR **'point-of-care ultrasound in microgravity'** OR ((**'point of care'**/exp OR **'point of care'**) AND (**'ultrasound'**/exp OR **ultrasound**) AND **in** AND (**'microgravity'**/exp OR **microgravity**)) OR **'point-of-care ultrasound at high altitude'** OR ((**'point of care'**/exp OR **'point of care'**) AND (**'ultrasound'**/exp OR **ultrasound**) AND **at** AND **high** AND (**'altitude'**/exp OR **altitude**)) AND 'English (language)'

Web of Science: 7

“point-of-care ultrasound in austere environments” OR “point-of-care ultrasound in military combat” OR “point-of-care ultrasound military” OR “point-of-care ultrasound prehospital” OR “point-of-care ultrasound in resource-limited settings” OR “point-of-care ultrasound low and middle income countries” OR “point-of-care ultrasound in microgravity” OR “point-of-care ultrasound at high altitude”

Total: 1159

Without duplicates: 766
