# Supplementary material for: Point-of-care ultrasound use in austere environments: A scoping review
Source: PLoS One. 2024 Dec 5;19(12):e0312017. doi: 10.1371/journal.pone.0312017 (PMC11620461; doi:10.1371/journal.pone.0312017)
Supplement: S1 Table — (DOCX) [file pone.0312017.s003.docx]

**Supplementary Table 1a-f. Summary of included ultrasound in austere environments studies in narrative scoping review**

*All studies were screened and data extracted by the two reviewers (Aubree Anderson and Rebecca Theophanous) between August 6 – August 31, 2024. All 324 studies included in the table met all eligibility criteria for study inclusion.

*There was no missing data during data extraction.

| **Supplementary Table 1a-f. Summary of included ultrasound in austere environments studies in narrative scoping review** | | | | |
| --- | --- | --- | --- | --- |
| **Table 1a. Military medicine:** | | | | |
| **Author name (year)** | **Study design** | **Study topic** | **Study setting** | **Study Quality Assessment (GRADE certainty of evidence)** |
| Alonso (2017) | Case report | Swimming-induced pulmonary edema diagnosed with lung US | Bournemouth, UK | Very low |
| Backlund (2010) | prospective | Feasibility of training Army National Guard medics in focused cardiac US | Denver, CO | moderate |
| Balasoupramanien (2022) | Cross-sectional | Survey of military nurses deployed in Operation Barkhane on willingness to be trained in POCUS | France | low |
| Baribeau (2023) | Prospective cohort | 5-day POCUS course, motion analysis while performing RUSH exams | Dartmouth, NH | moderate |
| Betcher (2018) | prospective | ONSD measurement by military trainees after brief POCUS training session | Muskegon, MI | moderate |
| Blenkinsop (2023) | Case report | Radiologist guiding military frontline healthcare provider using teleultrasound | Lichfield, UK | Very low |
| Bornemann (2014) | Cross-sectional | Surveys of military family physicians’ perceptions of handheld POCUS device | Honolulu, HI | low |
| Cais (2022) | Cross-sectional survey | Anesthesia, intensive care, medicine, EM military physicians | Royal College of EM, London, UK | low |
| Carter (2018) | retrospective | Diagnostic accuracy of FAST performed by trauma bay physicians in 187 pts | Role 3 Medical Treatment Facility, Camp Bastion, Plymouth, UK | moderate |
| Dana (2023) | Narrative review | Barriers to POCUS implementation in low resource areas (rural, military, and conflict zones) | Tel Aviv, Israel; N'Djamena, Chad; Toronto, Canada | low |
| Dubecq (2021) | Retrospective observational multicenter study | POCUS triage and treatment for casualties treated by 5 medical teams in Africa and the Middle East | French Military Health Service | moderate |
| Dulchavsky (2002) | prospective | Diagnostic accuracy of lower extremity MSK US to xrays for long-bone fractures | Detroit, MI | moderate |
| Duncan (2020) | Cross-sectional | Survey of 279 clinicians working in Role 1 British Army on POCUS use | Defence Primary Healthcare (DPHC), England, UK | low |
| Gleeson (2018) | Narrative review | POCUS in trauma, procedural anesthesia and vascular access | University of Massachusetts, MA | moderate |
| Hall (2010) | Case report | MSK US diagnosis of complete patellar tendon rupture in deployed setting | Darnell Army Medical Center, Fort Hood, TX | Very low |
| Hampton (2016) | Narrative review | SOLCUS military program for medics | Washington University in St Louis, MO | moderate |
| Heiner (2010) | Cross-sectional | Fracture simulation model from turkey leg bone for miliary MSK fracture POCUS training | U.S. Army Special Forces, Seattle, WA | Low |
| Hile (2012) | Narrative review | POCUS by military medical technicians for cardiac, pneumothorax, fracture | Army Special Forces, Tacoma, WA | low |
| Hubler (2010) | Case series | Special Forces Medical Sergeant fracture detection using POCUS vs xrays | U.S. Army | Very low |
| LaDuke (2017) | Prospective observational | Non-physician U.S. Army medical providers using POCUS to detect soft tissue abscess | Madigan Army Medical Center, Tacoma, WA | high |
| McNeil (2009) | Prospective observational | Military EM physician MSK fracture identification POCUS vs xray | Brooke Army Medical Center, San Antonio, TX | moderate |
| Merrill (2021) | Case report | Gunshot wound with pulmonary contusion, EFAST | Brooke Army Medical Center, San Antonio, TX | Very low |
| Monti (2020) | prospective | Impact of 4-hour POCUS training program in U.S. Army medics eFAST application | Madigan Army Medical Center, Tacoma, WA | moderate |
| Morgan (2010) | retrospective | Training of Special Operation Forces medics in POCUS | U.S. Army | low |
| Mount (2023) | Cross-sectional comparison | IV fluid bags vs gel standoff vs water bath for MSK POCUS | Madigan Army Medical Center, Tacoma, WA | low |
| Nations (2011) | Narrative review | Overview of history, current state, limitations, and future of POCUS in the battlefield | National Naval Medical Center, Bethesda, MD | moderate |
| Perrier (2020) | prospective | Assessment of POCUS training in French military medicine residents | France | moderate |
| Qi (2019) | Meta-analysis | FAST for injury assessment in military settings | Liaoning Province, China | high |
| Renard (2019) | prospective | Feasibility of EFAST during medical courses in hostile battlefield environment | Service Médical du Bataillon des Marins Pompiers de Marseille, Marseille, France | moderate |
| Rippey (2009) | Narrative review | Military uses of POCUS (EFAST, MSK, procedural guidance) | Nedlands, Australia | moderate |
| Rozanski (2005) | prospective | POCUS use in forward-deployed military hospital | Operation Iraqi Freedom | moderate |
| Rozycki (1999) | Narrative review | Surgeon-performed POCUS for abdominal injuries | Emory, GA | low |
| Rupp (2018) | Case report | Novel POCUS technique of systematic sonography of the abdomen in child in northern Iraq | Northern Iraq | Very low |
| Savell (2021) | Narrative review | Diagnostic accuracy of POCUS by military clinicians | United States | moderate |
| Sellon (2021) | Prospective, cross-sectional | Survey of role 2 hospital setting military clinicians performing FAST | Gosport, UK | low |
| Shokoohi (2018) | Narrative review | POCUS utility in targeted automobile ramming mass casualty attacks | Boston, MA | low |
| Snyder (2023) | Case report | POCUS by Special Operations Forces medical personnel for diagnosis and treatment of rib fractures in 47M | United States | Very low |
| Stephens (2000) | Cross-sectional | Survey of maternal beliefs on prenatal US at military medical center | Uniformed Services, Bethesda, MD | low |
| Whitfield (2012) | Case report | Facial gunshot wound with closed globe retinal detachment | Iraqi police officer | Very low |

| **Table 1b. Pre-hospital medicine:** | | | | |
| --- | --- | --- | --- | --- |
| **Author name (year)** | **Study design** | **Study topic** | **Study setting** | **Study Quality Assessment (GRADE certainty of evidence)** |
| Canadian Agency for Drugs and Technologies in Health (2015) | Narrative review - book | Portable US use in the pre-hospital setting (diagnostic and procedural, e.g. FAST, cardiac, lung for pneumothorax, aortic aneurysm, vascular access, fractures, etc.) | Ottawa, Canada | Moderate |
| Amaral (2020) | Narrative review | History of prehospital US, training needs, skill maintenance, QA, devices, and indications | Gainesville, Florida, USA | Moderate |
| Aziz (2024) | Retrospective database review | POCUS archive system implementation and governance in helicopter EMS | United Kingdom | Low |
| Bhat (2015) | Prospective observational study | Prehospital evaluation of effusion, pneumothorax, and standstill via POCUS | California, USA | Low |
| Blaivas (2010) | Prospective study | Assessment of prehospital needle chest thoracostomy via ED POCUS | Northside Hospital Forsyth, Georgia, USA | Moderate |
| Bobbia (2018) | Cross-sectional, descriptive, multicenter survey | Change in availability of prehospital and bedside POCUS | France | Low |
| Bobbia (2014) | Cross-sectional, descriptive, multicenter survey | Determining the availability of ultrasound devices | France | Low |
| Bobbia (2015) | Prospective observational study (monocentric) | Evaluating interpretability of prehospital echo based on physician experience level | Nimes University Hospital, France | Low |
| Botker (2018) | Systematic review | Role of POCUS in prehospital critical care (feasibility, changes in management, education) | N/A | High |
| Brun (2014) | RCT | On-site vs during transfer EFAST | France | High |
| Brun (2014) | Case report | Prehospital POCUS in blunt thoracic trauma | France | Very low |
| Brun (2013) | Case report | Prehospital echocardiogram in shock | France | Very low |
| Brun (2014) | Prospective observational study | Prehospital gastric tube confirmation by US | France | Low |
| Charron (2015) | Prospective study | Assessing the feasibility of prehospital portable US device use | France | Moderate |
| Chen (2022) | RCT, blinded | Novice prehospital EMS provider POCUS use with or without teleultrasound | Saba, Israel | High |
| Chenaitia (2012) | Prospective multicenter study | Prehospital gastric tube confirmation by US | Marseille, France  Grasse, France | Moderate |
| Chin (2013) | Prospective, educational intervention pilot study | Viability of Prehospital Assessment with UltraSound for Emergencies (PAUSE) protocol | San Antonio Military Medical Center, Houston, Texas | Moderate |
| Cover (2019) | Prospective study | Flight nurse US protocol creation | Ann Arbor, Michigan, USA | Moderate |
| Dan (2010) | Retrospective study | US applications in an MCI: earthquake | Chengdu Military General Hospital, China | Low |
| Darocha (2014) | Case Reports | POCUS during air rescue operations | Poland | Very low |
| Dewar (2022) | Educational study | Simulated prehospital needle thoracostomy with portable US | Rural North America | Low |
| Eimer (2024) | Cross-sectional (Nationwide survey) | POCUS education and experience | Germany | Low |
| Engelsen (2024) | Feasibility study | Telemonitored EFAST in flight | Oslo, Norway | Moderate |
| Fitzgerald (2024) | Prospective study | Prehospital thoracic US in respiratory distress for diagnosis and treatment | USA | Moderate |
| Fitzgibbon (2019) | Retrospective study | Feasibility of out-of-hospital cardiac arrest US by EMS physicians | Pennsylvania, USA | Low |
| Foster (2021) | Narrative review | POCUS in pediatric and neonatal transport |  | Moderate |
| Griffiths (2021) | Systemic review and meta-analysis | Helicopter POCUS use for pneumothorax | United Kingdom | High |
| Gundersen (2023) | Prospective observational study | Prehospital US in undifferentiated dyspnea | Denmark | Low |
| Guy (2019) | Prospective study | Implementation and evaluation of prehospital US curriculum | British Columbia, Canada | Moderate |
| Hafner (2024) | Feasibility study | Prehospital POCUS livestream during CPR | Vienna, Austria | Moderate |
| Harjola (2020) | Retrospective study/survey | Assessing the difficulty of diagnosis and management protocols for acute heart failure | Multinational (Finland, Spain, France, Belgium, Switzerland, USA) | Low |
| Heegaard (2010) | Prospective pilot project | Assessment of paramedic use and interpretation of FAST and abdominal aortic US | Hennepin County Medical Center, Minneapolis, MN, USA | Moderate |
| Heiner (2010) | Educational study | Simulated prehospital identification by US of simulation long bone fractures | Madigan Army Medical Center, Tacoma, WA, USA | Moderate |
| Hermann (2022) | Feasibility study | Remote real-time supervision of prehospital POCUS | Vienna, Austria | Moderate |
| Hill (2024) | Case report | Confirmation of mechanical capture in prehospital transcutaneous pacing by cardiac US | University of Wisconsin-Madison, Madison, WI | Very low |
| Holscher (2008) | Feasibility study | Prehospital transcranial duplex use | University of California San Diego, San Diego, California, USA | Moderate |
| Hoyer (2010) | Feasibility study | Evaluation of handheld US use in helicopters and emergency vehicles | Maximillians University of Munich, Munich, Germany | Moderate |
| Humphries (2023) | Case report | Prehospital carotid artery US indicates ROSC in OHCA | USA | Very low |
| Ienghong (2022) | Cross-sectional observational study | Prehospital handheld US device use and accuracy | Thailand | Low |
| Ienghong (2023) | Cross-sectional study | Impact of POCUS on ED length of stay | Thailand | Low |
| Ienghong (2023) | Retrospective observational study | International elective program with prehospital POCUS training | Laos | Moderate |
| Javaudin (2019) | Study protocol for a prospective multicenter observational study | Early POCUS with asystole, predictive value for the absence of ROSC in OHCA | France | Low |
| Jonck (2024) | Prospective observational study and comparison | Paramedic POCUS curriculum development and evaluation | Germany | Moderate |
| Jones (2024) | Case report | POCUS guided prehospital pericardiocentesis | Madison, WI, USA | Very low |
| Jorgensen (2010) | Systematic review | Review of impact of prehospital POCUS in trauma patients | Copenhagen, Denmark | High |
| Joyce (2020) | Educational study | Educating critical care medics in US verification of ETT placement (simulated cases) | Virginia Commonwealth University, Richmond, VA, USA | Low |
| Ketelaars (2013) | Retrospective observational study | Prehospital helicopter thoracic US | Netherlands | Low |
| Ketelaars (2018) | Narrative review | Overview of prehospital civilian setting US literature | Netherlands | Moderate |
| Khalil (2021) | RCT | Education of paramedics in recognizing tension pneumothorax via POCUS (simulated) | Denver, Colorado, USA | High |
| Knott (2024) | Narrative review | Discussion of advantages and disadvantages of prehospital POCUS | Berlin, Germany | Moderate |
| Kowalczyk (2023) | Narrative review | Review of prehospital thoracic/lung US in dyspnea | Poland | Moderate |
| Kowalcyzk (2023) | Prospective pilot study | Education of paramedics in lung US | Poland | Moderate |
| Kowalcyzk (2024) | Prospective pilot study | Combined cardiac and pulmonary US for prehospital diagnosis of pleural effusion | Poland | Moderate |
| Kreiser (2022) | Prospective observational study | Education of paramedics to assess for cardiac motion in OHCA | USA | Low |
| Kuttab (2021) | Case report | Diagnosis of right heart dilation, concerning for PE, in a patient with respiratory failure via prehospital echocardiogram | Madison, WI, USA | Very low |
| Lahham (2015) | Prospective educational intervention | Assessing paramedic scans for adequacy for clinical decision making | UC Irvine, Orange, California, USA | Moderate |
| Lapostolle (2022) | Narrative review | Prehospital US in cardiac emergencies | France | Moderate |
| Lauridsen (2024) | Retrospective cohort study | Process of care and outcome of patients with or without prehospital POCUS in ruptured abdominal aortic aneurysms | Denmark | Low |
| Lema (2018) | Prospective observational study | Paramedic use of US to identify esophageal intubation in cadavers | USA | Low |
| Lenz (2021) | Retrospective chart review | Chart review to determine which helicopter EMS population would benefit from POCUS | Milwaukee, WI, USA | Low |
| Lobo (2022) | Prospective cohort | Cardiac POCUS training in two remote locations, (SUB N and SUB S) | Portugal | Moderate |
| Martinet (2024) | Case report | Prehospital use of POCUS in SCUBA diving accidents | France | Very low |
| Mason (2019) | Prospective educational intervention | Education of flight nurses in identifying esophageal intubation and pneumothorax by POCUS | University of Washington, Seattle, WA, USA | Moderate |
| Mazur (2007) | Case report | Use of POCUS by a critical care retrieval service | Australia | Very low |
| Merlin (2020) | Case report | Initial case of transesophageal echocardiogram use in an out of hospital setting during CPR | N/A | Very low |
| Micheller (2019) | Educational curriculum design | Curriculum to assess and develop POCUS skills in prehospital providers | USA | Low |
| Michels (2023) | Consensus paper | Structured training recommendations/quality assurance for out of hospital POCUS in Germany | Germany | Low |
| Murali (2022) | Case report | Diagnosis of cardiac tamponade via POCUS during critical care transport | Toledo, OH, USA | Very low |
| Murray (2020) | Case report | Diagnosis of decreased cardiac contractility via POCUS in flight | Toledo, OH, USA | Very low |
| Myers, (2021) | Retrospective chart review | Review of military after action reviews to assess use of FAST and EFAST | Texas, USA | Low |
| Nadim (2021) | Educational intervention study | Prehospital POCUS and blood testing in COPD; prehospital treat-and-release program | Denmark | Low |
| Naeem (2023) | Prospective cohort study | Feasibility and impact of a hybrid POCUS educational program for London’s air ambulance | London, United Kingdom | Moderate |
| Naeem (2022) | Cross-sectional (survey) | National survey of use, governance, and perception of POCUS in prehospital care | United Kingdom | Low |
| Nelson (2008) | Narrative review | Summary of EMS use of POCUS | N/A | Moderate |
| Nelson (2016) | Narrative review | Description of POCUS use in prehospital, austere, and remote settings | N/A | Moderate |
| O’Connor (2023) | Prospective pre-post intervention study | evaluation of a teaching curriculum for EFAST, helicopter EMS providers | University of Massachusetts, Worcester, MA, USA | Moderate |
| Press (2013) | Prospective pre-post interventional study | Evaluation of a teaching curriculum for EFAST, aeromedical providers | University of Texas Health Science Center at Houston, Houston, TX, USA | Moderate |
| Prosen (2011) | Prospective study | Prehospital diagnosis of acute heart failure vs COPD/asthma using POCUS | Center for Emergency Medicine, Maribor, Slovenia | Moderate |
| Robinson (2023) | Case series | Prehospital diagnosis of evidence of massive PE by nonphysician providers using POCUS | Hennepin County Medical Center, Minneapolis, Minnesota, USA | Very low |
| Roline (2013) | Prospective pilot study | Performance of lung US in flight transport and evaluation of accuracy of interpretation | Hennepin County Medical Center, Minneapolis, MN, USA | Moderate |
| Ronaldson (2020) | Prospective observational study | Assessment of prehospital non-physician lung US image acquisition and interpretation | United Kingdom | Low |
| Rooney (2016) | Prospective educational intervention | Paramedics without POCUS experience were taught cardiac US | Orange County, Henry Ford, Detroit, MI | Moderate |
| Ross (2015) | Narrative review | Discussion of US exams relevant to the prehospital setting and options for continued training and skill maintenance | N/A | Moderate |
| Scharonow (2018) | Prospective observational cohort study | Evaluation of proportion, quality, and related changes in management of prehospital POCUS | Germany | Low |
| Schoeneck (2021) | Prospective observational pilot study | Assessment of feasibility and diagnostic quality of paramedic thoracic US | USA | Low |
| Sedlakova (2020) | Cross-sectional observational study (survey) | Survey of prehospital US use in aeromedical providers | Canada | Low |
| Shaw (2023) | Prospective observational study | Assessing whether early/prehospital EFAST would improve patient outcomes | Arizona, USA | Low |
| Siu (2023) | Prospective cohort study | Effect of telementoring in EFAST performed by critical care transport providers | Worcester, MA, USA | Moderate |
| Smith (2024) | Case series | Evaluation of effect of prehospital POCUS on critical care management | N/A | Low |
| Snaith (2011) | Feasibility study | Comparison of US exams in an ambulance vs simulated ED | UK | Moderate |
| Sotak (2023) | Case report | Role of POCUS in cardiac arrest, prehospital setting | Czech Republic | Very low |
| Stralec (2024) | Multicenter retrospective study | Evaluating for an association between a positive prehospital FAST and severe bleeding | France | Low |
| Strnad (2016) | Prospective observational pilot study | Assessment of prehospital lung US in monitoring patients on CPAP vs standard treatment | Slovenia | Low |
| Vianen (2023) | Prospective cohort study | Frequency of use of POCUS and changes in management in helicopter EMS settings | Netherlands | Moderate |
| Vicent (2024) | Narrative review | Discussion of current prehospital POCUS (benefits, limitations) and further opportunities | Germany | Moderate |
| Von Foerster (2024) | Narrative review | Prehospital US by EMS personnel, flight | University of Pittsburgh, PA | Moderate |
| Walcher (2002) | Prospective observational study | Assessment of use and benefit of FAST on scene | Germany | Low |
| Yates (2017) | Prospective observational study | Comparison of prehospital EFAST findings with those of ED trauma team and CT or operative reports | Southeast Virginia and Northwest North Carolina, USA | Low |

| **Table 1c. Resource-limited settings including low- and middle-income countries** | | | | |
| --- | --- | --- | --- | --- |
| **Author name (year)** | **Study design** | **Study topic** | **Study setting** | **Study Quality Assessment (GRADE certainty of evidence)** |
| Abbattista (2024) | Narrative review | POCUS in African hospitals and rural settings, training and needs | Africa | low |
| Abdo-Cuza (2019) | Narrative review | POCUS in critically ill patient in developing South American countries, strategies to expand adoption | Latin America, Chile, Argentina, Cuba | low |
| Abrokwa (2022) | Narrative review | Task shifting for POCUS in primary healthcare settings in LMIC. (obstetrics, gynecology, EM, infectious diseases, cardiac, abdominal, lung) | Institute of Tropical Medicine and International Health, Charité-Universitätsmedizin Berlin, Germany | moderate |
| Akanuwe (2023) | Narrative review | Qualitative study on community POCUS in the UK, barriers and facilitators to implementation | Lincoln, England, UK | high |
| Amoah (2016) | Prospective cohort | Mobile phone app and portable US to boost antenatal care and hospital OB deliveries in Ghana | Four rural communities in Central Ghana | moderate |
| Asfaw (2023) | Case report | POCUS use to diagnosis penile cellulitis | Addis Ababa, Ethiopia | Very low |
| Aspler (2022) | Cross-sectional | Establishing a self-sustaining POCUS curriculum in a teaching hospital in Ethiopia (FAST, IVC, lung identified by resident surveys) | Ethiopia | low |
| Baker (2021) | Retrospective review | POCUS by physicians in mobile Uganda clinics | Rural Uganda | moderate |
| Baloescu (2022) | Systematic review | POCUS in resource-limited settings | New Haven, CT | high |
| Barron (2018) | Prospective observational | POCUS use in short-term medical mission to rural Nicaragua | Sebaco, Nicaragua | moderate |
| Becker (2016) | Systematic review | Handheld US devices in LMIC, clinical applications, geographical areas, impact on pt management | St Joseph, Ann Arbor, MI | high |
| Belard (2024) | Narrative review | FASH protocol for HIV-positive pts with TB | Institute of Tropical Medicine, University of Tübingen, Tübingen, Germany | moderate |
| Belard (2016) | Narrative review | FASH and FASE (echinococcosis) protocols for tropical infectious diseases | Center of Tropical Medicine and Travel Medicine, Department of Infectious Diseases, Division of Internal Medicine, University of Amsterdam, The Netherlands | moderate |
| Bell (2016) | Prospective cohort | Pilot POCUS training program for 81 trainees in Kenya (EFAST, obstetric) | Nairobi, Kenya | moderate |
| Bentley (2015) | Prospective cohort | OB US curriculum for 31 midwives in Liberia | Liberia | moderate |
| Beye (2023) | Prospective observational | 12-month observation of serial lung US in 159 COVID-19 patients in Mali ICU | Point G Teaching Hospital, Bamako, Mali | high |
| Bidner (2023) | Cross-sectional | Exploratory needs-analysis survey of OB antenatal US in 114 rural/remote healthcare clinicians in Australian clinics | Adelaide, Australia | low |
| Bista (2022) | Case report | DVT US in 53M ED pt with left leg pain and swelling | Kathmandu, Nepal | Very low |
| Bittaye (2024) | Case report | Liver abscess US in 21F pt in RLS | Banjul, Gambia | Very low |
| Bitter (2018) | Cross-sectional | Survey of ED resources including POCUS in single Ugandan ED | District-level hospital, Uganda, rural sub-Saharan Africa | low |
| Blaivas (2005) | Prospective observational | POCUS effects on medical decision making of two physicians in the Amazon jungle (FAST, biliary, obstetric, renal, aorta) | Nomatzieguenga and Ashaninca tribal people, remote medical clinic in Amazon jungle | moderate |
| Bobbio (2019) | Cross-sectional | FASH exam in 100 HIV-positive pts being evaluated for TB in South Sudan | Yirol Hospital, South Sudan | low |
| Bouros (2024) | Prospective observational | EFAST exam implementation at Romanian hospital ED | "St. Spiridon" Emergency Clinical County Hospital, Iasi, Romania. | moderate |
| Bukhman (2019) | Narrative review | Acute heart failure diagnosis and management with cardiac US in Sub-Saharan Africa | Brigham and Women’s, Boston, MA; Kigali, Rwanda | low |
| Burleson (2020) | curriculum development | PURLS fellowship for novel POCUS curriculum in RLS | University of Alabama at Birmingham, AL | moderate |
| Burleson (2020) | retrospective | Handheld POCUS by 5 EM physicians in African ED | University of Alabama at Birmingham, AL; African ED | moderate |
| Chanler-Berat (2016) | Case report | 11M ED pt with typhoid intestinal perforation and +FAST on US | Nyakibale Hospital, Uganda | Very low |
| Chao (2015) | Case report | Liver abscess on US | Lohia Hospital, New Delhi, India | Very low |
| Chavez (2015) | Prospective cohort | Lung US to diagnosis childhood pneumonia in RLS; agreement between WHO algorithm | Peru, Nepal | high |
| Chen (2022) | RCT | POCUS use in 37 novice military or civilian EMS providers, with and without teleultrasound | Hospital Management, Meir Medical Center, Kfar Saba and Tel Aviv, Israel. | high |
| Crosby (2018) | Case report | POCUS diagnosis of mesenteric ischemia in 28M ED pt | Urban ED, Black Lion Hospital, Addis Ababa, Ethiopia | Very low |
| Crouch (2010) | Prospective cohort | FAST exam perceived confidence in healthcare providers pre/post-training workshop | Cusco, Peru | moderate |
| Denny (2018) | Prospective cohort | POCUS curriculum implementation assessment in four medical school classes in LMIC | Tandabui Institute of Health Sciences and Technology, Tanzania | high |
| Dieiev (2024) | Cross-sectional | Survey of 255 anesthesiologist/intensivists in POCUS course in Ukraine | Kiev, Ukraine | low |
| Doig (2019) | Scoping review | Antenatal OB US needs assessment in RLS | Africa, Asia, Central America and Australia | high |
| Doniger (2018) | Case report | 27F with abdominal pain and icterus, echinococcus cyst on US | Peru | Very low |
| Douglas-Vail (2023) | Case report | 14M with cerebral malaria, ONSD measurement with ocular US | Liberia | Very low |
| Dreyfuss (2020) | curriculum development | First ultrasound fellowship curriculum development in Peru, with teleultrasound | Lima, Peru | moderate |
| Edwards (2023) | Narrative review | Focused cardiac US in pregnant pts for rheumatic heart disease in LMIC | Heart Institute of the Caribbean, Kingston, Jamaica | low |
| Ekambaram (2023) | Case series | Focused cardiac US for acute left-sided cardiac valve emergencies in RLS | Emergency Department at Port Shepstone Regional Hospital, a rural, resource-limited hospital in KwaZulu-Natal, South Africa | low |
| Farahmand (2020) | Prospective cohort | Diagnostic accuracy of cardiac US for acute heart failure diagnosis in LMIC | Imam Khomeini hospital, Tehran, Iran | high |
| Fentress (2018) | Narrative review | Four case-based reviews: pediatric pneumonia; extrapulmonary TB; ectopic pregnancy; and tropical parasitic diseases | Contra Costa Regional Medical Center, Martinez, California | low |
| Fleshner (2022) | curriculum development | POCUS curriculum development for IM residents rotating in LMIC (most common: FASH, cardiac, lung US) | University of Colorado, Cleveland Clinic, Emory University, University of Pittsburgh | moderate |
| Gammeltoft (2007) | Qualitative | Qualitative interviews, Prenatal OB US screening, ethical dilemmas | Vietnam | high |
| Ganchi (2023) | Narrative review | Overview of spectrum and functionality of POCUS devices in LMIC and RLS | University of KwaZulu-Natal, Durban, South Africa | moderate |
| Garcia (2024) | Case report | 58F from Guyana with severe rheumatic mitral stenosis on cardiac US | Guyana | Very low |
| Gingrich (2013) | Case report | Young pt in Haiti with intussusception on bowel US | Haiti | Very low |
| Ginsburg (2021) | Prospective cohort | Comparison of lung US and chest xray in childhood pneumonia pts | Two district hospitals in Mozambique and Pakistan | moderate |
| Ginsburg (2023) | Cross-sectional | Survey of 241 healthcare providers using POCUS in LMIC | Seattle, WA; Ontario, Canada | low |
| Giordani (2013) | retrospective | US diagnosis of extrapulmonary TB in 243 HIV-positive pts | san Bortolo Hospital, Vicenza, Italy | moderate |
| Giordani (2018) | retrospective | US diagnosis of pneumocystis jirovecii pneumonia in 273 HIV-positive pts | san Bortolo Hospital, Vicenza, Italy | moderate |
| Godown (2015) | Prospective cohort | Handheld cardiac US vs auscultation for rheumatic heart disease diagnosis in Ugandan schoolchildren | Gulu, Uganda | high |
| Grenar (2024) | Curriculum development | Cardiac US training program for ED physicians in Czech Republic | University Hospital Hradec Králové, Czech Republic | moderate |
| Habibullah (2023) | Case report | MSK US to diagnose Achilles tendon rupture and calcaneal bone avulsion fracture in 55M ED pt | Karachi, Pakistan | Very low |
| Haider (2017) | Prospective cohort | Diagnostic accuracy of POCUS vs conventional US in Rwanda hospital | Centre Hospitalier Universitaire, Kigali, Rwanda, Africa | high |
| Hailemariam (2023) | Systematic review | Lung US for leishmaniasis diagnosis in RLS | Addis Ababa, Ethiopia; London, UK | high |
| Haldeman (2022) | Curriculum development | POCUS curriculum implementation assessment at Family Medicine program in Zambia | Lusaka, Zambia | moderate |
| Hall (2021) | Curriculum development | New antenatal US curriculum assessment for healthcare providers in Tanzania | Zanzibar, Tanzania | moderate |
| Heller (2017) | Cross-sectional | Needs assessment, survey of 100 Malawi pts (**FAST, cardiac, DVT**, liver, renal, gynecology) | Malawi, Sub-Saharan Africa | low |
| Heller (2024) | Prospective observational | PUSH protocol (liver US) for antiviral treatment initiation in chronic hepatitis B in LMIC | Lilongwe, Malawi | moderate |
| Henry (2020) | Case series | 5 pediatric pts with meningococcaemia, femoral central line insertion, ventricular septal defect (cardiac), leg abscess, rheumatic heart disease | Santo Espiritu, Vanuatu | Very low |
| Henwood (2014) | Curriculum development | Mix-methods focus group and surveys of five Columbian EM residency programs for POCUS curriculum implementation | Colombia, South America | high |
| Henwood (2016) | Prospective cohort | POCUS training program in 17 Rwandan physicians | Kigali, Rwanda | high |
| Henwood (2014) | Curriculum development | Overview of core factors to consider for development and implementation of POCUS program in RLS | Brigham and Women’s, Boston, MA | moderate |
| Hill (2021) | Case report | Lung US diagnosis of pulmonary hydatid cyst causing shock in pediatric ED pt | Cusco, Peru | Very low |
| Huson (2019) | Narrative review | CURLS protocol (cardiac US) for heart failure diagnosis in Sub-Saharan Africa | Lilongwe, Malawi | moderate |
| Hussen (2024) | Case series | POCUS to assess diaphragmatic paralysis from Guillain-barre syndrome in RLS | Harar, Ethiopia | Very low |
| Lenghong (2021) | Curriculum development | Learner surveys for teleultrasound and novel EM resident POCUS curriculum in RLS | Khon Kaen, Thailand | moderate |
| Lenghong (2023) | Curriculum development, retrospective | Survey of Laos EM residents’ experience with international elective rotation at Khon Kaen University | Khon Kaen, Thailand | moderate |
| Jhagru (2023) | Curriculum development, cross-sectional | POCUS curriculum assessment, EM residents and registrars in Guyana | Guyana, South America | high |
| Jones (2020) | Prospective cohort | Survey assessment of 3-day POCUS workshop for family medicine physicians in Kenya | Nairobi, Kenya | moderate |
| Kaminecki (2023) | Systematic review | POCUS for dehydration assessment in children, IVC/Ao ratio | Augusta, GA | high |
| Kaminstein (2019) | Narrative review | POCUS for diagnosis and staging of schistosomiasis and echinococcosis in LMIC | Lelongwe, Malawi; Verona, Italy | low |
| Khanyi (2021) | retrospective | Review of POCUS done in 987 pts at South African ED | General Justice Gizenga Mpanza Regional Hospital, KwaZulu-Natal, South Africa | moderate |
| Kizito (2023) | Prospective cohort | Diagnostic accuracy of cardiac/lung US vs chest xray in ED pts with hypoxia in Uganda | Mbarara Regional Referral Hospital (MRRH), Uganda | high |
| Klassen (2022) | Narrative review | POCUS for rheumatic heart failure in Sub-Saharan Africa | Rwinkwavu, Rwanda | low |
| Kobal (2004) | prospective | Feasibility and diagnostic accuracy of handheld cardiac US in 126 pts in cardiology clinic in rural Mexico | Rural Mexico | high |
| Kodaira (2021) | Prospective observational | Diagnosis using handheld smartphone-based POCUS in maternity referral hospital in Sierra Leone | Sierra Leone | high |
| Kovacevic (2019) | Prospective cohort | Case-based structured teleultrasound course implementation assessment in MICU in Bosnia and Herzegovina | Banja Luka, Bosnia and Herzegovina | high |
| Lamorte (2016) | Prospective observational | SLURP for ultrasound effectiveness in outpatient POCUS diagnosis | Holy Spirit Hospital, Makeni, Sierra Leone | high |
| Leopold (2018) | prospective | Lung US for malaria and ARDS/sepsis diagnosis | Bangladesh | moderate |
| Limani (2021) | Prospective observational | Weekly record review of POCUS done in medical inpatients | Queen Elizabeth Central Hospital, Malawi | moderate |
| Lu (2015) | Prospective cohort | Handheld cardiac US performed in five schools in Gulu, Uganda for heart valve abnormality detection | Gulu, Uganda | high |
| Luntsi (2022) | Scoping review | FASH in HIV-positive pts for TB diagnosis | Maiduguri, Nigeria | high |
| Martin (2022) | Case series | Teaching US nerve blocks with teleultrasound in Peru | Cusco, Peru | low |
| Martins (2021) | Prospective cohort | Accuracy and reliability of focused cardiac US in pts with Chagas disease | Belo Horizonte, Brazil | high |
| Maw (2019) | Qualitative interviews | Qualitative interviews on stakeholder perceptions of POCUS implementation in two hospitals in Haiti and Malawi | Haiti and Malawi | high |
| Mazmanyan (2020) | Prospective cohort | Lung US vs clinical respiratory diagnosis in neonatal pts in three NICUs in developing countries | Yerevan, Armenia; Paris, France | high |
| Mbanjumucyo (2016) | Case series | FASH exam for TB diagnosis in 3 HIV-positive patients | Rwanda | Very low |
| Modi (2016) | Prospective cohort | Accuracy of IVC US for dehydration in children with diarrhea in RLS | rehydration unit of the International Centre for Diarrhoeal Disease Research, Bangladesh | high |
| Moore (2015) | Prospective cohort | Accuracy of OB US gestational age in antenatal clinics | Shoklo Malaria Research Unit on the Thai-Myanmar border | high |
| Muhame (2021) | Prospective cohort | Remote case review of cardiac US for congenital and acquired heart disease diagnosis in 233 children in RLS | Médecins Sans Frontières, Toronto, Canada; Geneva, Switzerland | high |
| Mulye (2023) | Curriculum development | Hybrid in-person and virtual POCUS program on lung US for healthcare providers in Belize | Punta Gorda, Belize | moderate |
| Muriuki (2024) | prospective cohort | Quasi-experimental, CURLS protocol implementation in 45 graduate medical pre-interns who are novice POCUS users for cardiac US competency | Kenyatta National Hospital in Nairobi, Kenya | high |
| Musolino (2024) | Cross-sectional | Exploratory survey of current POCUS state in Italian pediatric settings | Italy | low |
| Nacarapa (2022) | prospective | In-hospital mortality due to extrapulmonary TB per clinical and POCUS features (FASH exam) | Carmelo Hospital of Chókwè district, Mozambique | high |
| Nadimpalli (2019) | prospective | Feasibility of training mid-level clinical officers to perform POCUS algorithm for pediatric lung US | Médecins Sans Frontières, Aweil, South Sudan | high |
| Ndege (2023) | RCT | FASH exam in HIV-positive pts for TB diagnosis vs routine care | Ifakara, United Republic of Tanzania | high |
| Neugebauer (2018) | Case series | Two cases of FASH exam and enterobiliary fistula for TB diagnosis in HIV-positive pts | Ifakara, United Republic of Tanzania | Very low |
| Ngome (2020) | Narrative review | POCUS use in trauma, TB, schistosomiasis, DVT, lung, and shock | Ifakara, United Republic of Tanzania | low |
| Nhat (2023) | Prospective cohort | Four user groups interpreting lung US, with or without AI | Ho Chi Minh City, Vietnam; London, UK; Bangkok, Thailand | high |
| Pellegrini (2018) | Cross-sectional | National survey on POCUS use in Brazilian ICUs | Brazil | low |
| Perks (2024) | Case report | US-guided pericardiocentesis from COVID-19 cardiac tamponade using central venous catheter | Rural Australia | Very low |
| Ploutz (2016) | prospective | Comparison of handheld cardiac US by two nurses in two Uganda schools vs reference standard for rheumatic heart disease diagnosis | Uganda Heart Institute, Kampala, Uganda | high |
| Polan (2014) | Prospective interventional | Assess effect of remote feedback using teleultrasound on POCUS skills of clinical providers in Uganda after training session | Nyakibale Hospital in rural Uganda | high |
| Raees (2024) | retrospective | ONSD on ocular US in pediatric cerebral malaria | Blantyre, Malawi | moderate |
| Rahulkumar (2019) | prospective | Diagnostic accuracy of RUSH in govt hospital in Central Gujarat | Central Gujarat, India | high |
| Raiten (2020) | Narrative review | Overview of current state of perioperative POCUS and TEE in Nepal and Bangladesh | Nepal and Bangladesh | moderate |
| Remppis (2020) | prospective | Protocol development of FASUS (renal US for schistosomiasis) in 118 pts with hematuria | Lambaréné, Gabon | high |
| Reynolds (2018) | Prospective cross-sectional | Impact of POCUS on clinical decision making at ED in Tanzania | Muhimbili National Hospital ED, Tanzania | high |
| Rimbaut (2022) | Cross-sectional | Survey of clinical providers on POCUS implementation and limitations in EDs in Belgium | Jan Palfijn Ziekenhuis Ghent, Brussels, Belgium | low |
| Rominger (2018) | Curriculum development | Assessment of POCUS curriculum implementation for local physicians in rural clinics in Chiapas, Mexico | Chiapas, Mexico | high |
| Sabatino (2020) | Curriculum development | Assessment of POCUS training program for community health officers on cardiac, lung, abdominal US, FAST | Lokomasama-a chiefdom of Sierra Leone | high |
| Salmon (2017) | Curriculum development | Overview and practical algorithmic approach to POCUS curriculum development and implementation in RLS | 8 African countries; Toronto, Canada | moderate |
| Schmidt (2022) | Curriculum development | Assessment of POCUS training program for lung and cardiac US in pediatric pts | Eastern Uganda | high |
| Sepulveda-Ortiz (2020) | Curriculum development | Assessment of POCUS training program for 13 pediatricians in Puerto Rican ED | Puerto Rico | high |
| Shaddock (2022) | Systematic review | Explore current state of POCUS use in rural Australia and other countries | New South Wales, Australia | high |
| Shah (2008) | Curriculum development | Review of POCUS training program implementation for local providers in two rural hospitals Rwanda | Rwanda | moderate |
| Shah (2016) | prospective | Assessment of focused cardiopulmonary US training program for healthcare ED providers in Haiti | Haiti | high |
| Shari (2018) | Case report | 3F diagnosis of purulent pericarditis with cardiac US | Muhimbili National Hospital in Dar es Salaam, Tanzania | Very low |
| Shokoohi (2019) | Cross-sectional | Survey of clinical educators trained in POCUS assessing the training program | Tanzania, Malawi, Uganda | low |
| Shorter (2012) | Retrospective observational | POCUS use in Haiti earthquake disaster in 2010, most common: RUSH, trauma, obstetrics, abdominal pain, DVT, cardiac, lung US, procedural | Gheskio Field clinic at Port-au-Prince, Haiti | moderate |
| Shrestha (2020) | prospective | Quasi-experimental, 2-day POCUS training workshop assessment for primary care providers | Nepal | high |
| Shumbusho (2020) | prospective | Accuracy of surgical resident-performed lung US vs chest xray in pneumothorax diagnosis | Rwanda | high |
| Smith (2010) | Prospective clinical study | Use of FAST exam in a rural ED, a referral center for 22 hospitals | KwaZulu-Natal (KZN), South Africa | Moderate |
| Spencer (2008) | retrospective | POCUS exams done at two primary care sites and two hospitals (MSK, obstetrics, breast, vascular, abdomen, renal) | Ghana Health Mission, Sekondi-Takoradi, Ghana | moderate |
| Stachura (2017) | Prospective observational | Impact of POCUS done in 118 patients in Ethiopian ED | Tikur Anbessa Specialized Hospital EC in Addis Ababa, Ethiopia | moderate |
| Stanley (2017) | Prospective observational | Impact of POCUS done in 55 inpatients in Tanzania hospital | Mwanza, Tanzania | moderate |
| Stolz (2015) | Prospective | POCUS curriculum impact for non-physician clinicians in Ugandan ED | Uganda | moderate |
| Stroffolini (2023) | Case report | POCUS in diagnosis of bacillary angiomatosis (FASH exam) | Verona, Italy | Very low |
| Sullivan (2024) | Case report | Cardiac US in 50M with HAPE and STEMI | Plaza de Mulas medical tent, Aconcagua, Argentina | Very low |
| Suttels (2023) | Qualitative study | Barriers and facilitators to lung US implementation in tertiary centre in Benin | Benin, Sub-Saharan Africa | high |
| Tafoya (2017) | prospective | Feasibility of implementing a POCUS program into EM resident curriculum in LMIC | Kumasi, Ghana | high |
| Terry (2019) | Prospective observational | POCUS incorporation into non-physician ED care providers training program in rural Uganda | Mbarara, Uganda | high |
| Thomas (2023) | qualitative | Mixed-methods health needs assessment, semi-structured interviews on POCUS access in rural populations in India | Chepauk, India | high |
| Toscano (2023) | prospective | Teleultrasound prenatal OB in Peru | San Miguel, Peru | high |
| Toscano (2021) | prospective | Diagnostic accuracy of three POCUS devices for gynecologic US for RLS | Rochester, NY | high |
| Tran (2021) | Narrative review | POCUS in anesthesiology in LMIC | University of Colorado | moderate |
| Trovato (2016) | Narrative review | Lung US in RLS | Ragusa, Italy | low |
| Umuhire (2019) | Prospective cohort | POCUS for pts with shortness of breath in ED | University Teaching Hospital of Kigali (UTH-K) in Rwanda | moderate |
| Van Hoving (2019) | Scoping review | FASH exam for HIV-positive pts and extrapulmonary TB diagnosis | Cape Town, South Africa | high |
| Vyas (2018) | Prospective | Feasibility of medical students using ROUTE (rural OB US) exam in Panama | Bocas del Toro, Panama | moderate |
| Wachira (2023) | prospective | Assessment of POCUS training program implementation in 514 rural healthcare providers in Kenya | Nairobi, Kenya | high |
| Wangmang (2020) | Case report | Cardiac US in 53M with dilated cardiomyopathy with shortness of breath diagnosing cardiac mass | Uganda ED, Sub-Saharan Africa | Very low |
| Wanjiku (2024) | qualitative | Mixed-methods study exploring the impact of POCUS use on referral decisions in healthcare providers at primary rural and peri-urban facilities | Nairobi, Kenya | high |
| Waweru-Siika (2022) | Narrative review | Overview of focused cardiac US by non-cardiologists in LMIC | Nairobi, Kenya; Oxford, UK | low |
| Weimer (2024) | Curriculum development | Effectiveness of POCUS training program for treatment of cancer pts | Gutenberg University, Mainz, Germany | moderate |
| Yao (2020) | Curriculum development | Effectiveness of longitudinal POCUS training program in rural hospital in Haiti | Alma Mater Hospital, Haiti | high |

| **Table 1d. Microgravity and outer space:** | | | | |
| --- | --- | --- | --- | --- |
| **Author name (year)** | **Study design** | **Study topic** | **Study setting** | **Study Quality Assessment (GRADE certainty of evidence)** |
| Asachi (2023) | Scoping review | US in microgravity: abdominal, lung, DVT, sinusitis, MSK, renal, ocular, decompression sickness | International Space Station (ISS) or parabolic flight | moderate |
| Fincke (2005) | Prospective cohort | Just-in-time training algorithm with remote guidance for MSK US of the shoulder | ISS, NASA, Houston, TX | moderate |
| Fischetti (2024) | Narrative review | Suggested POCUS topics and curriculum for astronauts in space | Boston, MA | low |
| Garcia (2018) | Prospective cohort | Just-in-time training of comprehensive spinal US before flight, in flight, and after flight | ISS | moderate |
| Hamilton (2004) | Cross-sectional | Microgravity simulation for parabolic flight of pneumothorax in pig models | Wyle Laboratories, Houston, TX | low |
| Johansen (2018) | Systematic review | Lung US | Outer space | high |
| Karlsson (2009) | Cross-sectional | Doppler US for venous gas emboli and decompression illness in hypobaric chamber simulation | Stockholm, Sweden | low |
| Kirkpatrick (2019) | Narrative review | EFAST and teleultrasound for space simulation | NASA and the Canadian Space Agency | low |
| Marshall (2019) | Prospective cohort | Internal jugular flow on US during and after spaceflight in seated, supine, and 15 degree head tilt | ISS, NASA | moderate |
| Marshburn (2014) | Prospective cohort | Just-in-time training and remote US guidance for spinal US in microgravity | ISS, NASA | moderate |
| Martin (2003) | Narrative review | Cardiac and spinal US, fluid shift in microgravity, decompression sickness | NASA, Houston, TX | moderate |

| **Table 1e. High altitude:** | | | | |
| --- | --- | --- | --- | --- |
| **Author name (year)** | **Study design** | **Study topic** | **Study setting** | **Study Quality Assessment (GRADE certainty of evidence)** |
| Fagenhold (2009) | Cross-sectional | Ocular US, increased optic nerve sheath diameter association with AMS diagnosis | Mountains in Pheriche, Nepal | low |
| Galdamez (2017) | Review and algorithm creation for pilots | POCUS in high altitude free fall and parachuting injuries | Houston, TX | Very low |
| Holtholf (2021) | Case report | POCUS to diagnosis pneumonia in patient with respiratory distress at high altitude | Khumbu valley of Nepal | Very low |
| Kirkpatrick (2001) | Case report | Lung US pneumothorax evaluation in skier with hemoptysis and shortness of breath after a fall | Blackcomb Mountain, British Columbia, Canada | Very low |
| Kranc (2019) | Case report | shoulder dislocation reduction | Wilderness hiking | Very low |
| Lahham (2023) | Prospective cohort | Lung US B line assessment for HAPE at high altitudes | Mammoth Mountain, CA, USA | moderate |
| Lochner (2015) | Narrative review | Ocular US, increased ONSD association with AMS | Merano, Italy | moderate |
| Nolting (2019) | Case report | Solar powered POCUS | Himalayan hiking, India | Very low |
| Paziana (2012) | Case series | Foreign body removal with soft tissue US | Wilderness, Thomas Jefferson, Philadelphia, PA | Very low |
| Subedi (2023) | Case series | HAPE, lung US | Mt Everest | Very low |
| Sullivan (2024) | Systematic review | Ocular US, increased ONSD association with AMS | Merano, Italy | high |
| Sutherland (2008) | Prospective cohort | Ocular US, increased ONSD association with AMS | Mt Everest | moderate |
| Tremblay (2018) | Prospective cohort | Brachial and superficial femoral arterial duplex US in lowlanders vs Sherpa guides at high altitudes | UBC-Nepal | moderate |
| Weber (2018) | Prospective cohort | Lung US for HAPE and ONSD diagnosis of AMS in lung transplant pts vs normal controls | Mt Kilamanjaro | moderate |
| Wippling (2021) | Case report | Lung US for HAPE and ONSD diagnosis of AMS | Khumbu valley of Nepal | Very low |

| **Table 1f. General point-of-care ultrasound use in multiple austere environments:** | | | | |
| --- | --- | --- | --- | --- |
| **Author name (year)** | **Study design** | **Study topic** | **Study setting** | **Study Quality Assessment (GRADE certainty of evidence)** |
| Buerger (2017) | Narrative review | Diagnostic and procedural POCUS use, education and training, comparison of POCUS to other imaging modalities | Emergency medicine, military, remote care | moderate |
| Canepa (2019) | Narrative review | pulmonary, ocular, vascular, trauma | Wilderness, post-disaster, trauma, global health | moderate |
| Germonpre (2014) | Prospective cohort, validation study | Frame-based venous gas embolism counting in decompression sickness | Diving and hyperbaric medicine, Europe | moderate |
| Gharahbaghian (2017) | Narrative review | EFAST, MSK, soft tissue, HAPE, pneumonia, volume status, procedures (nerve blocks, abscess, etc) | Military, flight, disaster, global health, outer space, high altitude | moderate |
| Haines (2023) | Cross-sectional study | Image quality pre/post- transmission | Roanoke, VA | low |
| Henwood (2014) | Review/guidelines | Needs assessment, POCUS equipment, training, | Global health, Boston, MA | low |
| Kaminecki (2023) | Systematic review | POCUS for dehydration in children, IVC/Aorta ratio | 2 USA, 3 remote sites | high |
| Kirkpatrick (2021) | Case report (proof of concept) | Remotely piloted aerial systems (RPAS), Drone delivery of teleultrasound device for lung US | University of Calgary and Alberta Institute of Technology, SwissDrones | Very low |
| Levine (2016) | RCT equivalence | RAPID study for regional anesthesia with or without ultrasound | Earthquake disasters, Brown, RI | high |
| Lipsitz (2022) | Scoping Review | All POCUS exams | Military, disaster, global health, outer space, high altitude | high |
| Marshburn (2004) | Prospective observational cohort | Diagnostic accuracy of long-bone MSK US after one-hour training session | Three academic teaching EDs (Boston and Cambridge, MA; Houston, TX; Detroit, MI) | moderate |
| Maw (2019) | Qualitative, semi-structured interviews, Framework | POCUS program implementation evaluation | Global health | high |
| McNeil (2009) | Prospective observational | Diagnostic accuracy of long-bone MSK US by EM physician | Brooke Army Medical Center, San Antonio, TX | moderate |
| Milton (2018) | Prospective, comparative study | Glaucomannan powder gel vs standard ultrasound gel | Army Medical Center, Tacoma, WA | moderate |
| Moser (2023) | Case series | Locoregional nerve blocks in caving accidents rescue | Switzerland | low |
| Nelson (2011) | Narrative review | Trauma, casualty triage, pneumothorax, acute mt sickness, ONSD, fracture, lung, soft tissue, MSK | Military, disaster, global health, outer space, high altitude | moderate |
| Nelson (2016) | Narrative review | Trauma, casualty triage, pneumothorax, acute mt sickness, ONSD, fracture, lung, soft tissue, MSK | Military, disaster, global health, outer space, high altitude | moderate |
| Paziana (2012) | Case series (x2) | Two reports of foreign body removal using wilderness medical field kits | Thomas Jefferson, Philadelphia, PA | Very low |
| Ramsingh (2019) | Case report | Smartphone video conference app for POCUS in resource-limited setting | Hôpital Adventiste d’Haiti | Very low |
| Roy (2019) | Prospective sed crossover study | Handheld POCUS exposed to cold with or without chemical foot warmers | McGill University, Canada | high |
| Russell (2013) | Narrative review | POCUS in extreme and austere environments (PTX, pericardial effusion, abdominal trauma, MSK, HAPE, ocular, obstetrics, AMS, stroke) | Space, swamp, jungle, mountain, desert; Las Vegas, NV | moderate |
| Shorter (2012) | Retrospective observational study | Shock, trauma, pregnancy, abdomen, DVT, lung, procedural | Earthquake disaster, Haiti | moderate |
| Stanley (2017) | Cross-sectional study | FAST, cardiac, lung, abdomen, DVT, soft tissue | Hospital inpatients, Tanzania | low |
| Todorovic (2024) | Cross-sectional survey | 3M Defib-pads vs standard ultrasound gel | Canada | Very low |
| Volpicelli (2012) | Expert consensus guidelines using delphi technique | Expert consensus on Lung US from three conferences, 73 guidelines | International Liaison on Lung US in Bologna, Pisa, and Rome, Italy | high |
| Whitfield (2012) | Case report | Ocular US for retinal detachment diagnosis from facial gunshot wound | Naval Medical Center, San Diego, CA | Very low |
| Woo (2014) | Narrative review | abdominal, fetal, cardiac, musculoskeletal and vascular. | Haiti earthquake, New Mexico natural disaster, Vancouver 2010 Olympics, Queensland Australia hospice care | low |
| Wray (2023) | Noninferiority study | 9 common liquids vs standard ultrasound gel | Dartmouth, NH | moderate |
| Wydo (2016) | Narrative focused review | POCUS use in mass casualty and disaster settings | Camden, NJ | moderate |
| Zadel (2015) | Prospective cohort | Diagnostic accuracy of POCUS for endotracheal tube position in prehospital intubation | Maribor, Slovenia | moderate |

**Key**: Point-of-care ultrasound (POCUS), EFAST (extended focused assessment with sonography in trauma), DVT (deep venous thrombosis), AMS, acute mountain sickness, TB (tuberculosis), FASH (focused assessment with sonography for HIV), HIV (human immunodeficiency virus). EM (Emergency Medicine), RCT (randomized control trial), SOLCUS (Special Operator Level Clinical Ultrasound), GRADE (Grading of Recommendations, Assessment, Development, and Evaluations), US (ultrasound), QA (quality assurance), EMS (Emergency Medical Services), ED (Emergency Department), MCI (mass casualty incident), CPR (cardiopulmonary resuscitation), ROSC (return of spontaneous circulation), OHCA (out of hospital cardiac arrest), ETT (endotracheal tube), PE (pulmonary embolism), SCUBA (self-contained underwater breathing apparatus), COPD (chronic obstructive pulmonary disease), CPAP (continuous positive airway pressure)
