# Supplementary material for: Point-of-care ultrasound use in austere environments: A scoping review
Source: PLoS One. 2024 Dec 5;19(12):e0312017. doi: 10.1371/journal.pone.0312017 (PMC11620461; doi:10.1371/journal.pone.0312017)
Supplement: S2 Table — (DOCX) [file pone.0312017.s004.docx]

| **Supplemental Table 2. Austere ultrasound in prehospital and resource-limited settings summaries** | | |
| --- | --- | --- |
| **Prehospital studies summary:** | **Article study design** | **Author name (date) or point-of-care ultrasound examination type** |
|  | 3 RCT | Brun (2014), Chen (2022), Khalil (2021) |
|  | 2 systematic reviews | Botker (2018), Jorgensen (2010) |
|  | 12 narrative reviews | CADTH (2015), Amaral (2020), Foster (2021), Knott (2024), Kowalczyk (2023), Lapostolle (2022), Nelson (2008, 2016), Ross (2015), Vicent (2024), Von Foerster (2024) |
|  | 38 prospective observational/cohort, 7 cross-sectional | -Lung (pneumothorax, B lines, effusion)  -Cardiac (pericardial effusion, cardiac standstill)  -procedures (gastric tube, intubation)  -EFAST, abdominal aorta  -portable US feasibility and availability  -US protocols (PAUSE, EMS, flight)  -Education and curriculum development |
|  | 13 case reports | Lung (pneumothorax), Cardiac (effusion, pacing), cardiac arrest (carotid US pulse), prehospital EMS, air flight, SCUBA |
|  | 2 case series | Right heart dilation, critical care management |
|  | 10 retrospective | -POCUS archival by flight medics  -POCUS in MCI earthquake  -Feasibility of POCUS in cardiac arrest by EMS  -Lung, cardiac, and FAST, and aorta POCUS education |
|  | 6 feasibility pilot studies | Engelson (2024), Hafner (2024), Hermann (2022), Holscher (2008), Hoyer (2010), Snaith (2011) |
|  | 5 educational curriculum or simulation | Dewar (2022), Heiner (2010), Joyce (2020), Micheller (2019), Nadim (2021) |
|  | 1 consensus paper | Michels (2023), structured training recommendations |

| **RLS including LMIC studies summary:** | **Article study design** | **Author name (date) or point-of-care ultrasound examination type** |
| --- | --- | --- |
|  | 5 systematic reviews | Baloescu (2022), Becker (2016), Hailemariam (2023), Kaminecki (2023), Shaddock (2022) |
|  | 3 scoping review | Doig (2019), Luntsi (2022), Van Hoving (2019) |
|  | 18 narrative reviews | -antenatal care,  -POCUS training and needs assessment (Africa, Argentina, Germany, UK)  -FASH protocol for TB diagnosis in HIV patients (Germany, Netherlands)  -Cardiac US for heart failure and disease (Rwanda, Kenya, Jamaica, Sub-Saharan Africa, Nepal, Bangladesh)  -Lung US (Italy)  -Spectrum of POCUS devices (South Africa)  -Echinococcosis (Italy) |
|  | 52 prospective observational/cohort | -Mobile phone app for antenatal care (Ghana, Sierra Leone, Thai-Myanmar border)  -Mission trip to Nicaragua; Nepal  -Pilot POCUS training program in Kenya, Peru (EFAST, obstetric); Liberia, Panama (Obstetric); Romania, Peru, South Africa (EFAST)  -Medical decision-making in Amazon jungle  -Lung US for childhood pneumonia diagnosis (Armenia, France, Peru, South Sudan); pediatric cardiac US (Uganda)  -Cardiac/lung US (Uganda, Mexico, Brazil, Bangladesh, Kenya, Switzerland, Vietnam, Rwanda)  -Chronic hepatitis (PUSH protocol)  -Dehydration/diarrhea (IVC)  -RUSH protocol (Gujarat, India)  -FASH exam (extrapulmonary TB in Mozambique)  -FASUS protocol (urinary schistosomiasis in Gabon)  -SLURP protocol for outpatient POCUS diagnosis  -General POCUS (Haiti, Ethiopia, Malawi, Rwanda, Tanzania)  -POCUS curriculum implementations (Kenya, Liberia, Rwanda, Peru, Bosnia and Herzegovina, Ghana, Uganda, Nepal) |
|  | 13 cross-sectional | -Antenatal US needs (Australian clinics)  -ED POCUS resources (Uganda, Tanzania, Belgium)  -FASH exam in HIV-positive patients for TB evaluation (South Sudan)  -Anesthesia/intensivist POCUS course (Ukraine)  -POCUS survey (Canada; Seattle, WA; Malawi; Italy; Brazil)  -POCUS curriculum (Guyana, Tanzania, Malawi, Uganda) |
|  | 17 case reports, 6 case series | -cerebral malaria ONSD  -bacillary angiomatosis (FASH exam)  -deep venous thrombosis  -Intussusception  -liver abscess  -mesenteric ischemia  -intestinal perforation with positive FAST exam  -echinococcus (Peru)  -penile cellulitis  -pediatric case series (Vanuatu)  -rheumatic heart disease; cardiac mass  -Achilles tendon rupture  -ruptured pulmonary hydatid cyst (Peru)  -pulmonary aspergillosis (Uganda)  -US nerve block (Peru)  -purulent pericarditis (Muhumbili)  -diaphragm paralysis from Guillain-Barre syndrome |
|  | 1 prospective cohort, 1 cross-sectional, 2 narrative reviews, 2 scoping review, 1 curriculum development, 3 case reports, 1 RCT | 11 TB/FASH/HIV studies |
|  | 3 prospective cohort, 3 cross-sectional | 6 Lung/COVID-19 studies |
|  | 1 prospective cohort, 3 narrative reviews | 4 acute heart failure/cardiac studies |
|  | 1 retrospective, 5 prospective, 1 systematic review | 7 handheld African ED and 5 handheld cardiac studies |
|  | 25 prospective | 25 POCUS curriculum evaluation studies |

**Key**: Point-of-care ultrasound (POCUS), EFAST (extended focused assessment with sonography in trauma), DVT (deep venous thrombosis), AMS, acute mountain sickness, TB (tuberculosis), FASH (focused assessment with sonography for HIV), HIV (human immunodeficiency virus). EM (Emergency Medicine), RCT (randomized control trial), SOLCUS (Special Operator Level Clinical Ultrasound), GRADE (Grading of Recommendations, Assessment, Development, and Evaluations), US (ultrasound), QA (quality assurance), EMS (Emergency Medical Services), ED (Emergency Department), MCI (mass casualty incident), CPR (cardiopulmonary resuscitation), ROSC (return of spontaneous circulation), OHCA (out of hospital cardiac arrest), ETT (endotracheal tube), PE (pulmonary embolism), SCUBA (self-contained underwater breathing apparatus), COPD (chronic obstructive pulmonary disease), CPAP (continuous positive airway pressure)
