# Supplementary material for: Point-of-care ultrasound use in austere environments: A scoping review
Source: PLoS One. 2024 Dec 5;19(12):e0312017. doi: 10.1371/journal.pone.0312017 (PMC11620461; doi:10.1371/journal.pone.0312017)
Supplement: S3 Table — (DOCX) [file pone.0312017.s005.docx]

**Supplementary Table 3**

**Austere ultrasound scoping review: included studies**

1. (2015). CADTH Rapid Response Reports. Portable Ultrasound Devices in the Pre-Hospital Setting: A Review of Clinical and Cost-Effectiveness and Guidelines. Ottawa (ON), Canadian Agency for Drugs and Technologies in Health
2. Abbattista, T., et al. (2024). "The urgent need to extend the appropriate use of ultrasound in Africa and worldwide. Overview, experiences and perspectives." Front Public Health 12: 1363134.
3. Abdo-Cuza, A. and P. Blanco (2019). "[Point-of-care ultrasound in critically ill patients in developing countries of Latin America]." Medwave 19(9): e7709.
4. Abrokwa, S. K., et al. (2022). "Task shifting for point of care ultrasound in primary healthcare in low- and middle-income countries-a systematic review." EClinicalMedicine 45: 101333.
5. Akanuwe, J. N. A., et al. (2023). "Practitioners' views on community implementation of point-of-care ultrasound (POCUS) in the UK: a qualitative interview study." BMC Health Serv Res 23(1): 84.
6. Alonso, J. V., et al. (2017). "Swimming-induced pulmonary oedema an uncommon condition diagnosed with POCUS ultrasound." Am J Emerg Med 35(12): 1986.e1983-1986.e1984.
7. Amaral, C. B., et al. (2020). "Prehospital point-of-care ultrasound: A transformative technology." SAGE Open Med 8: 2050312120932706.
8. Amoah, B., et al. (2016). "Boosting antenatal care attendance and number of hospital deliveries among pregnant women in rural communities: a community initiative in Ghana based on mobile phones applications and portable ultrasound scans." BMC Pregnancy Childbirth 16(1): 141.
9. Asachi, P., et al. (2023). "Utility of ultrasound in managing acute medical conditions in space: a scoping review." Ultrasound J 15(1): 47.
10. Asfaw, Y. A., et al. (2023). "Diagnosing Cellulitis of the Penis with Point-of-Care Ultrasonography in a Resource-Limited Setting." Case Rep Emerg Med 2023: 1626736.
11. Aspler, A., et al. (2022). "Establishing a Self-sustaining Emergency Medicine Point-of-Care Ultrasound Curriculum in an Academic Teaching Hospital in Ethiopia." Ethiop J Health Sci 32(3): 533-538.
12. Aziz, S., et al. (2024). "Implementation of a point-of-care ultrasound archiving system and governance framework in a UK physician-paramedic staffed helicopter emergency medical service." Scand J Trauma Resusc Emerg Med 32(1): 49.
13. Backlund, B. H., et al. (2010). "Pilot study to determine the feasibility of training Army National Guard medics to perform focused cardiac ultrasonography." Prehosp Emerg Care 14(1): 118-123.
14. Baker, D. E., et al. (2021). "Impact of point-of-care ultrasound on the diagnosis and treatment of patients in rural Uganda." Trop Doct 51(3): 291-296.
15. Balasoupramanien, K., et al. (2022). "Ultrasonography Performed by Military Nurses in Combat Operations: A Perspective for the Future?" J Spec Oper Med 22(3): 65-69.
16. Baloescu, C., et al. (2022). "Effect of Point-of-Care Ultrasound on Clinical Outcomes in Low-Resource Settings: A Systematic Review." Ultrasound Med Biol 48(9): 1711-1719.
17. Baribeau, V., et al. (2023). "Motion Analysis: An Objective Assessment of Special Operations Forces and Tactical Medics Performing Point-of-Care Ultrasound." J Spec Oper Med 23(1): 67-73.
18. Barron, K. R., et al. (2018). "Point-of-Care Ultrasound as Part of a Short-Term Medical Mission to Rural Nicaragua." South Med J 111(7): 434-438.
19. Becker, D. M., et al. (2016). "The use of portable ultrasound devices in low- and middle-income countries: a systematic review of the literature." Trop Med Int Health 21(3): 294-311.
20. Belard, S., et al. (2024). "Point-of-care ultrasound for tuberculosis and HIV-revisiting the focused assessment with sonography for HIV-associated tuberculosis (FASH) protocol and its differential diagnoses." Clin Microbiol Infect 30(3): 320-327.
21. Bélard, S., et al. (2016). "Point-of-Care Ultrasound Assessment of Tropical Infectious Diseases--A Review of Applications and Perspectives." Am J Trop Med Hyg 94(1): 8-21.
22. Bell, G., et al. (2016). "A pilot training program for point-of-care ultrasound in Kenya." Afr J Emerg Med 6(3): 132-137.
23. Bentley, S., et al. (2015). "Evaluation of an Obstetric Ultrasound Curriculum for Midwives in Liberia." J Ultrasound Med 34(9): 1563-1568.
24. Betcher, J., et al. (2018). "Military trainees can accurately measure optic nerve sheath diameter after a brief training session." Mil Med Res 5(1): 42.
25. Beye, S. A., et al. (2023). "Assessment of lung injury severity using ultrasound in critically ill COVID-19 patients in resource limited settings." Ann Intensive Care 13(1): 33.
26. Bhat, S. R., et al. (2015). "Prehospital Evaluation of Effusion, Pneumothorax, and Standstill (PEEPS): Point-of-care Ultrasound in Emergency Medical Services." West J Emerg Med 16(4): 503-509.
27. Bidner, A., et al. (2023). "Antenatal ultrasound needs-analysis survey of Australian rural/remote healthcare clinicians: recommendations for improved service quality and access." BMC Public Health 23(1): 2268.
28. Bista, S., et al. (2022). "Knowledge of point-of-care ultrasound and management of deep vein thrombosis patient in resource limited setup: A case report." Ann Med Surg (Lond) 77: 103702.
29. Bittaye, S. O., et al. (2024). "The Use of Point-of-Care Ultrasound in the Diagnosis and Percutaneous Aspiration of Liver Abscess in a Resource-Limited Country: A Case Report." Cureus 16(7): e63905.
30. Bitter, C. C., et al. (2018). "What resources are used in emergency departments in rural sub-Saharan Africa? A retrospective analysis of patient care in a district-level hospital in Uganda." BMJ Open 8(2): e019024.
31. Blaivas, M. (2010). "Inadequate needle thoracostomy rate in the prehospital setting for presumed pneumothorax: an ultrasound study." J Ultrasound Med 29(9): 1285-1289.
32. Blaivas, M., et al. (2005). "Change in differential diagnosis and patient management with the use of portable ultrasound in a remote setting." Wilderness Environ Med 16(1): 38-41.
33. Blenkinsop, G., et al. (2023). "Remote ultrasound diagnostics disrupting traditional military frontline healthcare delivery." BMJ Mil Health 169(5): 456-458.
34. Bobbia, X., et al. (2018). "Changes in the availability of bedside ultrasound practice in emergency rooms and prehospital settings in France." Anaesth Crit Care Pain Med 37(3): 201-205.
35. Bobbia, X., et al. (2014). "Availability and practice of bedside ultrasonography in emergency rooms
36. Bobbia, X., et al. (2015). "Does physician experience influence the interpretability of focused echocardiography images performed by a pocket device?" Scand J Trauma Resusc Emerg Med 23: 52.
37. Bobbio, F., et al. (2019). "Focused ultrasound to diagnose HIV-associated tuberculosis (FASH) in the extremely resource-limited setting of South Sudan: a cross-sectional study." BMJ Open 9(4): e027179.
38. Bornemann, P. and G. Bornemann (2014). "Military family physicians' perceptions of a pocket point-of-care ultrasound device in clinical practice." Mil Med 179(12): 1474-1477.
39. Bøtker, M. T., et al. (2018). "The role of point of care ultrasound in prehospital critical care: a systematic review." Scand J Trauma Resusc Emerg Med 26(1): 51.
40. Bouros, G. C., et al. (2024). "A Modern Diagnostic Procedure-The Introduction of Point-of-Care Ultrasound in Romanian Emergency Physicians' Daily Routine." Clin Pract 14(3): 1137-1148.
41. Brun, P. M., et al. (2014). "Stay and play eFAST or scoop and run eFAST? That is the question!" Am J Emerg Med 32(2): 166-170.
42. Brun, P. M., et al. (2014). "Prehospital ultrasound thoracic examination to improve decision making, triage, and care in blunt trauma." Am J Emerg Med 32(7): 817.e811-812.
43. Brun, P. M., et al. (2013). "The value of prehospital echocardiography in shock management." Am J Emerg Med 31(2): 442.e445-447.
44. Brun, P. M., et al. (2014). "2-point ultrasonography to confirm correct position of the gastric tube in prehospital setting." Mil Med 179(9): 959-963.
45. Buerger, A. M. and K. R. Clark (2017). "Point-of-Care Ultrasound: A Trend in Health Care." Radiol Technol 89(2): 127-138.
46. Bukhman, A. K., et al. (2019). "Diagnosis and Management of Acute Heart Failure in Sub-Saharan Africa." Curr Cardiol Rep 21(10): 120.
47. Burleson, S. L., et al. (2020). "Point-of-care ultrasound in resource-limited settings: the PURLS fellowship." Ultrasound J 12(1): 14.
48. Burleson, S. L., et al. (2020). "Evaluation of a novel handheld point-of-care ultrasound device in an African emergency department." Ultrasound J 12(1): 53.
49. Cais, S. and J. Winchester (2022). "Point of care ultrasound within the Defence Medical Services." Anaesthesia 77: 22.
50. Canepa, C. A. and N. S. Harris (2019). "Ultrasound in Austere Environments." High Alt Med Biol 20(2): 103-111.
51. Carter, N. J. and D. Gay (2018). "FAST in the deployed military setting." J R Army Med Corps 164(5): 332-334.
52. Chanler-Berat, J., et al. (2016). "Typhoid intestinal perforation: Point-of-care ultrasound as a diagnostic tool in a rural Ugandan Hospital." Afr J Emerg Med 6(1): 44-46.
53. Charron, C., et al. (2015). "Difficulties encountered by physicians in interpreting focused echocardiography using a pocket ultrasound machine in prehospital emergencies." Eur J Emerg Med 22(1): 17-22.
54. Chavez, M. A., et al. (2015). "Agreement Between the World Health Organization Algorithm and Lung Consolidation Identified Using Point-of-Care Ultrasound for the Diagnosis of Childhood Pneumonia by General Practitioners." Lung 193(4): 531-538.
55. Chen, J., et al. (2022). "A randomized, controlled, blinded evaluation of augmenting point-of-care ultrasound and remote telementored ultrasound in inexperienced operators." Isr Med Assoc J 24(9): 596-601.
56. Chenaitia, H., et al. (2012). "Ultrasound to confirm gastric tube placement in prehospital management." Resuscitation 83(4): 447-451.
57. Chin, E. J., et al. (2013). "A pilot study examining the viability of a Prehospital Assessment with UltraSound for Emergencies (PAUSE) protocol." J Emerg Med 44(1): 142-149.
58. Cover, M., et al. (2019). "Creation of a Flight Nurse Critical Care Ultrasound Program." Air Med J 38(4): 266-272.
59. Crosby, J., et al. (2018). "Acute mesenteric ischaemia: A case of expedited diagnosis and management using point-of-care ultrasound." Afr J Emerg Med 8(4): 164-166.
60. Crouch, A. K., et al. (2010). "Perceived confidence in the FAST exam before and after an educational intervention in a developing country." Int J Emerg Med 3(1): 49-52.
61. Dan, D., et al. (2010). "Ultrasonographic applications after mass casualty incident caused by Wenchuan earthquake." J Trauma 68(6): 1417-1420.
62. Dana, E., et al. (2023). "Point-of-Care Ultrasound (PoCUS) and Its Potential to Advance Patient Care in Low-Resource Settings and Conflict Zones." Disaster Med Public Health Prep 17: e417.
63. Darocha, T., et al. (2014). "Point-of-care ultrasonography during rescue operations on board a Polish Medical Air Rescue helicopter." J Ultrason 14(59): 414-420.
64. Denny, S. P., et al. (2018). "Ultrasound curriculum taught by first-year medical students: A four-year experience in Tanzania." World J Emerg Med 9(1): 33-40.
65. Dewar, Z. E., et al. (2022). "Prehospital portable ultrasound for safe and accurate prehospital needle thoracostomy: a pilot educational study." Ultrasound J 14(1): 23.
66. Dieiev, V., et al. (2024). "Point-of-care ultrasonography in Ukraine: a survey of anesthesiologists-intensivists participating in ultrasonography courses." Can J Anaesth.
67. Doig, M., et al. (2019). "Exploring the availability and impact of antenatal point-of-care ultrasound services in rural and remote communities: A scoping review." Australas J Ultrasound Med 22(3): 174-185.
68. Doniger, S. J. and A. Wang (2018). "Icterus and abdominal pain: an unexpected, rare sonographic finding in a Peruvian Emergency Department." Crit Ultrasound J 10(1): 14.
69. Douglas-Vail, M., et al. (2023). "Optic Disc Elevation Secondary to Cerebral Malaria Resolves Completely With Mannitol Administration and Corresponds to Clinical Improvement." Cureus 15(6): e40639.
70. Dreyfuss, A., et al. (2020). "A Novel Multimodal Approach to Point-of-Care Ultrasound Education in Low-Resource Settings." West J Emerg Med 21(4): 1017-1021.
71. Dubecq, C., et al. (2021). "Point-of-care ultrasound for treatment and triage in austere military environments." J Trauma Acute Care Surg 91(2S Suppl 2): S124-s129.
72. Dulchavsky, S. A., et al. (2002). "Advanced ultrasonic diagnosis of extremity trauma: the FASTER examination." J Trauma 53(1): 28-32.
73. Duncan, P. G. A. and J. Mackey (2020). "Point-of-care ultrasound at Role 1: is it time for a rethink?" BMJ Mil Health 166(6): 406-410.
74. Edwards, P., et al. (2023). "Focused cardiac ultrasound in pregnancy." J Investig Med 71(2): 81-91.
75. Eimer, C., et al. (2024). "[Ultrasound diagnostics in prehospital emergency care-do we need a standardized educational approach?]." Med Klin Intensivmed Notfmed 119(4): 309-315.
76. Ekambaram, K. and K. Hassan (2023). "Establishing a Novel Diagnostic Framework Using Handheld Point-of-Care Focused-Echocardiography (HoPE) for Acute Left-Sided Cardiac Valve Emergencies: A Bayesian Approach for Emergency Physicians in Resource-Limited Settings." Diagnostics (Basel) 13(15).
77. Engelsen, P. C., et al. (2024). "Ascending with ultrasound: telementored eFAST in flight-a feasibility study." Emerg Radiol 31(1): 25-31.
78. Fagenholz, P. J., et al. (2009). "Optic nerve sheath diameter correlates with the presence and severity of acute mountain sickness: evidence for increased intracranial pressure." J Appl Physiol (1985) 106(4): 1207-1211.
79. Farahmand, S., et al. (2020). "Point-of-care ultrasound modalities in terms of diagnosing acute decompensated heart failure in emergency department; a diagnostic accuracy study." Intern Emerg Med 15(3): 491-499.
80. Fentress, M., et al. (2018). "Point-of-Care Ultrasound in Resource-Limited Settings: Common Applications." South Med J 111(7): 424-433.
81. Fincke, E. M., et al. (2005). "Evaluation of shoulder integrity in space: first report of musculoskeletal US on the International Space Station." Radiology 234(2): 319-322.
82. Fischetti, C., et al. (2024). "Space Ultrasound: A Proposal for Competency-based Ultrasound Training for In-flight Space Medicine." West J Emerg Med 25(2): 275-281.
83. Fitzgerald, E., et al. (2024). "Thoracic ultrasound may improve paramedic diagnostic and management accuracy in undifferentiated respiratory distress." J Am Coll Emerg Physicians Open 5(2): e13164.
84. Fitzgibbon, J. B., et al. (2019). "Feasibility of Out-of-Hospital Cardiac Arrest Ultrasound by EMS Physicians." Prehosp Emerg Care 23(3): 297-303.
85. Fleshner, M., et al. (2022). "Impact of Point-of-Care Ultrasound in Medical Decision Making: Informing the Development of an Internal Medicine Global Health POCUS Curriculum." Pocus j 7(1): 144-153.
86. Foale, C. M., et al. (2005). "Diagnostic instrumentation aboard ISS: just-in-time training for non-
87. Foster, B., et al. (2021). "Use of Point-of-Care Ultrasound in the Pediatric and Neonatal Emergency Transport Realm." Pediatr Ann 50(10): e432-e436.
88. Galdamez, L. A., et al. (2017). "Point-of-Care Ultrasound Utility and Potential for High Altitude Crew Recovery Missions." Aerosp Med Hum Perform 88(2): 128-136.
89. Gammeltoft, T. and H. T. Nguyen (2007). "Fetal conditions and fatal decisions: ethical dilemmas in ultrasound screening in Vietnam." Soc Sci Med 64(11): 2248-2259.
90. Garcia, K. M., et al. (2018). "Real-time Ultrasound Assessment of Astronaut Spinal Anatomy and Disorders on the International Space Station." J Ultrasound Med 37(4): 987-999.
91. Garcia Reinoso, L. and S. John (2024). "A Rare Case of Severe Mitral Stenosis Presenting As Cardiogenic Shock." Cureus 16(4): e57627.
92. Germonpré, P., et al. (2014). "The use of portable 2D echocardiography and 'frame-based' bubble counting as a tool to evaluate diving decompression stress." Diving Hyperb Med 44(1): 5-13.
93. Gharahbaghian, L., et al. (2017). "Point-of-Care Ultrasound in Austere Environments: A Complete Review of Its Utilization, Pitfalls, and Technique for Common Applications in Austere Settings." Emerg Med Clin North Am 35(2): 409-441.
94. Gingrich, A. S., et al. (2013). "Point-of-care ultrasound in a resource-limited setting: diagnosing intussusception." J Emerg Med 45(3): e67-70.
95. Ginsburg, A. S., et al. (2021). "Performance of lung ultrasound in the diagnosis of pediatric pneumonia in Mozambique and Pakistan." Pediatr Pulmonol 56(2): 551-560.
96. Ginsburg, A. S., et al. (2023). "A survey of barriers and facilitators to ultrasound use in low- and middle-income countries." Sci Rep 13(1): 3322.
97. Giordani, M. T., et al. (2013). "Extrapulmonary mycobacterial infections in a cohort of HIV-positive patients: ultrasound experience from Vicenza, Italy." Infection 41(2): 409-414.
98. Giordani, M. T., et al. (2018). "Point-of-care lung ultrasound for diagnosis of Pneumocystis jirovecii pneumonia: notes from the field." Crit Ultrasound J 10(1): 8.
99. Godown, J., et al. (2015). "Handheld echocardiography versus auscultation for detection of rheumatic heart disease." Pediatrics 135(4): e939-944.
100. Grenar, P., et al. (2024). "Point-of-Care Cardiac Ultrasound Training Programme: Experience from the University Hospital Hradec Králové." Emerg Med Int 2024: 9974284.
101. Griffiths, E. (2021). "Helicopter emergency medical services use of thoracic point of care ultrasound for pneumothorax: a systematic review and meta-analysis." Scand J Trauma Resusc Emerg Med 29(1): 163.
102. Gundersen, E. A., et al. (2023). "PrehospitaL Ultrasound in Undifferentiated DyspnEa (PreLUDE): a prospective, clinical, observational study." Scand J Trauma Resusc Emerg Med 31(1): 6.
103. Guy, A., et al. (2019). "A Blended Prehospital Ultrasound Curriculum for Critical Care Paramedics." Air Med J 38(6): 426-430.
104. Habibullah, N., et al. (2023). "Use of point-of-care ultrasound in a low-resource setting to diagnose Achilles tendon rupture and avulsion fracture of the calcaneal bone." Int J Emerg Med 16(1): 66.
105. Hafner, C., et al. (2024). "Live stream of prehospital point-of-care ultrasound during cardiopulmonary resuscitation - A feasibility trial." Resuscitation 194: 110089.
106. Haider, S. J. A., et al. (2017). "Prospective Comparison of Diagnostic Accuracy Between Point-of-Care and Conventional Ultrasound in a General Diagnostic Department: Implications for Resource-Limited Settings." J Ultrasound Med 36(7): 1453-1460.
107. Haines, C. M., et al. (2023). "Point-of-Care Ultrasound Transmission for Remote Interpretation in Austere Environments." Wilderness Environ Med 34(4): 420-426.
108. Haldeman, M. S., et al. (2022). "Resident perception on the impact of point-of-care ultrasound in clinical care at a family medicine training program in Zambia." Ultrasound J 14(1): 18.
109. Hall, B. T. and T. McArthur (2010). "Ultrasound diagnosis of a patellar tendon rupture." Mil Med 175(12): 1037-1038.
110. Hall, E. A., et al. (2021). "Analysis of an obstetrics point-of-care ultrasound training program for healthcare practitioners in Zanzibar, Tanzania." Ultrasound J 13(1): 18.
111. Hamilton, D. R., et al. (2004). "Sonographic detection of pneumothorax and hemothorax in microgravity." Aviat Space Environ Med 75(3): 272-277.
112. Hampton, K. K., et al. (2016). "SOLCUS: Update On Point-of-Care Ultrasound In Special Operations Medicine." J Spec Oper Med 16(1): 58-61.
113. Harjola, P., et al. (2020). "Pre-hospital management protocols and perceived difficulty in diagnosing acute heart failure." ESC Heart Fail 7(1): 289-296.
114. Heegaard, W., et al. (2010). "Prehospital ultrasound by paramedics: results of field trial." Acad Emerg Med 17(6): 624-630.
115. Heiner, J. D., et al. (2010). "The ultrasound detection of simulated long bone fractures by U.S. Army Special Forces Medics." J Spec Oper Med 10(2): 7-10.
116. Heiner, J. D. and T. J. McArthur (2010). "The ultrasound identification of simulated long bone fractures by prehospital providers." Wilderness Environ Med 21(2): 137-140.
117. Heller, T., et al. (2017). "Ultrasound for patients in a high HIV/tuberculosis prevalence setting: a needs assessment and review of focused applications for Sub-Saharan Africa." Int J Infect Dis 56: 229-236.
118. Heller, T., et al. (2024). "Point-of-care ultrasound to inform antiviral treatment initiation in chronic hepatitis B virus infection in low-resource settings - the PUSH protocol." Ultrasound J 16(1): 18.
119. Henry, J. (2020). "Paediatric point-of-care ultrasound in a resource-limited Melanesian setting: A case series." Australas J Ultrasound Med 23(1): 66-73.
120. Henwood, P. C., et al. (2014). "Characterizing the limited use of point-of-care ultrasound in Colombian emergency medicine residencies." Int J Emerg Med 7(1): 7.
121. Henwood, P. C., et al. (2016). "Intensive point-of-care ultrasound training with long-term follow-up in a cohort of Rwandan physicians." Trop Med Int Health 21(12): 1531-1538.
122. Henwood, P. C., et al. (2014). "A practical guide to self-sustaining point-of-care ultrasound education programs in resource-limited settings." Ann Emerg Med 64(3): 277-285.e272.
123. Hermann, M., et al. (2022). "Remote real-time supervision of prehospital point-of-care ultrasound: a feasibility study." Scand J Trauma Resusc Emerg Med 30(1): 23.
124. Hile, D. C., et al. (2012). "Is point-of-care ultrasound accurate and useful in the hands of military medical technicians? A review of the literature." Mil Med 177(8): 983-987.
125. Hill, A., et al. (2021). "Point-of-care Ultrasound Diagnosis of Pulmonary Hydatid Cyst Disease Causing Shock: A Case Report." Clin Pract Cases Emerg Med 5(4): 403-406.
126. Hill, M. A., et al. (2024). "Prehospital Cardiac Ultrasound to Confirm Mechanical Capture in Emergency Transcutaneous Pacing: A Case Report." Air Med J 43(4): 357-359.
127. Holscher, T., et al. (2008). "Transcranial ultrasound from diagnosis to early stroke treatment. 1. Feasibility of prehospital cerebrovascular assessment." Cerebrovasc Dis 26(6): 659-663.
128. Holthof, N., et al. (2021). "Point-of-Care Ultrasound Diagnosis of Community-Acquired Pneumonia in a High-Altitude, Resource-Poor Setting." Prehosp Emerg Care 25(6): 839-843.
129. Hoyer, H. X., et al. (2010). "Prehospital ultrasound in emergency medicine: incidence, feasibility, indications and diagnoses." Eur J Emerg Med 17(5): 254-259.
130. Hubler, D. A., et al. (2010). "Fracture detection in a combat theater: four cases comparing ultrasound to conventional radiography." J Spec Oper Med 10(2): 11-15.
131. Humphries, A. L., et al. (2023). "Paramedic-Performed Carotid Artery Ultrasound Heralds Return of Spontaneous Circulation in Out-of-Hospital Cardiac Arrest: A Case Report." Prehosp Emerg Care 27(1): 107-111.
132. Huson, M. A. M., et al. (2019). "Cardiac ultrasound in resource-limited settings (CURLS): towards a wider use of basic echo applications in Africa." Ultrasound J 11(1): 34.
133. Hussen, A., et al. (2024). "Point-of-Care Ultrasound to Assess Diaphragmatic Paralysis in Resource-Limited Setting: A Case Series." Int Med Case Rep J 17: 433-437.
134. Ienghong, K., et al. (2021). "Development and Remodeling of Point-of-Care Ultrasound Education for Emergency Medicine Residents in Resource Limited Countries during the COVID-19 Pandemic." Tomography 7(4): 721-733.
135. Ienghong, K., et al. (2022). "The Utilization of Handheld Ultrasound Devices in a Prehospital Setting." Prehosp Disaster Med 37(3): 355-359.
136. Ienghong, K., et al. (2023). "The Impact of Prehospital Point of Care Ultrasounds on Emergency Patients Length of Stay in Thailand." J Multidiscip Healthc 16: 219-226.
137. Ienghong, K., et al. (2023). "An in-Depth Examination of the Characteristics of Pre-Hospital Point-of-Care Ultrasound Training Among Emergency Medicine Residents in Laos as Part of an Overseas Elective Rotation." Adv Med Educ Pract 14: 1221-1229.
138. Javaudin, F., et al. (2019). "Early point-of-care focused echocardiographic asystole as a predictive factor for absence of return of spontaneous circulatory in out-of-hospital cardiac arrests: a study protocol for a prospective, multicentre observational study." BMJ Open 9(8): e027448.
139. Jhagru, R., et al. (2023). "Evaluation of an emergency medicine point-of-care ultrasound curriculum adapted for a resource-limited setting in Guyana." Int J Emerg Med 16(1): 57.
140. Johansen, B. D., et al. (2018). "Point-of-Care Ultrasound for Pulmonary Concerns in Remote Spaceflight Triage Environments." Aerosp Med Hum Perform 89(2): 122-129.
141. Jonck, C., et al. (2024). "Development and evaluation of a point-of-care ultrasound curriculum for paramedics in Germany - a prospective observational study and comparison." BMC Med Educ 24(1): 811.
142. Jones, J. D., et al. (2024). "Prehospital Ultrasound Use to Guide Emergent Pericardiocentesis: A Case Report." Air Med J 43(4): 360-362.
143. Jones, L., et al. (2020). "Expanding Point-of-Care Ultrasound Training in a Low- and Middle-Income Country: Experiences From a Collaborative Short-Training Workshop in Kenya." Fam Med 52(1): 38-42.
144. Jørgensen, H., et al. (2010). "Does prehospital ultrasound improve treatment of the trauma patient? A systematic review." Eur J Emerg Med 17(5): 249-253.
145. Joyce, M., et al. (2020). "Ability of Critical Care Medics to Confirm Endotracheal Tube Placement by Ultrasound." Prehosp Disaster Med 35(6): 629-631.
146. Kaminecki, I., et al. (2023). "Point-of-Care Ultrasonography for the Assessment of Dehydration in Children: A Systematic Review." Pediatr Emerg Care 39(10): 786-796.
147. Kaminstein, D., et al. (2019). "Sound Around the World: Ultrasound for Tropical Diseases." Infect Dis Clin North Am 33(1): 169-195.
148. Karlsson, L. L., et al. (2009). "Venous gas emboli and exhaled nitric oxide with simulated and actual extravehicular activity." Respir Physiol Neurobiol 169 Suppl 1: S59-62.
149. Ketelaars, R., et al. (2013). "Prehospital chest ultrasound by a dutch helicopter emergency medical service." J Emerg Med 44(4): 811-817.
150. Ketelaars, R., et al. (2018). "ABCDE of prehospital ultrasonography: a narrative review." Crit Ultrasound J 10(1): 17.
151. Khalil, P. A., et al. (2021). "Randomized Controlled Trial of Point-of-Care Ultrasound Education for the Recognition of Tension Pneumothorax by Paramedics in Prehospital Simulation." Prehosp Disaster Med 36(1): 74-78.
152. Khanyi, H. B. and B. Naicker (2021). "The use of point-of-care ultrasound in a regional emergency department in KwaZulu-Natal, South Africa." S Afr Fam Pract (2004) 63(1): e1-e6.
153. Kirkpatrick, A. W., et al. (2001). "Hand-held portable sonography for the on-mountain exclusion of a pneumothorax." Wilderness Environ Med 12(4): 270-272.
154. Kirkpatrick, A. W., et al. (2021). "Pioneering Remotely Piloted Aerial Systems (Drone) Delivery of a Remotely Telementored Ultrasound Capability for Self Diagnosis and Assessment of Vulnerable Populations-the Sky Is the Limit." J Digit Imaging 34(4): 841-845.
155. Kizito, P. M., et al. (2023). "Diagnostic Performance of Point of Care Ultrasound Compared to Chest X-Ray in Patients with Hypoxia at a Teaching Hospital Emergency Department in Uganda." Afr J Emerg Med 13(2): 61-67.
156. Klassen, S. L., et al. (2022). "Using Point-of-Care Ultrasound in Heart Failure Diagnosis and Management in Rural and Resource-Limited Settings." CASE (Phila) 6(6): 259-262.
157. Knott, D., et al. (2024). "[Sonography in Pre-clinical Care]." Dtsch Med Wochenschr 149(15): 912-924.
158. Kobal, S. L., et al. (2004). "Hand-carried cardiac ultrasound enhances healthcare delivery in developing countries." Am J Cardiol 94(4): 539-541.
159. Kodaira, Y., et al. (2021). "Reliability of ultrasound findings acquired with handheld apparatuses to inform urgent obstetric diagnosis in a high-volume resource-limited setting." Int J Gynaecol Obstet 153(2): 280-286.
160. Kovacevic, P., et al. (2019). "Impact of weekly case-based tele-education on quality of care in a limited resource medical intensive care unit." Crit Care 23(1): 220.
161. Kowalczyk, D., et al. (2023). "Concise, Practical Review on Transthoracic Lung Ultrasound in Prehospital Diagnosis of Dyspnea in Adults." Medicina (Kaunas) 59(2).
162. Kowalczyk, D., et al. (2023). "Ultrasound on the Frontlines: Empowering Paramedics with Lung Ultrasound for Dyspnea Diagnosis in Adults-A Pilot Study." Diagnostics (Basel) 13(22).
163. Kowalczyk, D., et al. (2024). "Unlocking Diagnostic Precision: FATE Protocol Integration with BLUE and eFAST Protocols for Enhanced Pre-Hospital Differential Diagnosis of Pleural Effusion Manifested as Dyspnea in Adults-A Pilot Study." J Clin Med 13(6).
164. Kranc, D. A., et al. (2019). "Use of Ultrasound for Joint Dislocation Reduction in an Austere Wilderness Setting: A Case Report." Prehosp Emerg Care 23(4): 584-589.
165. Kreiser, M. A., et al. (2022). "Point-of-Care Ultrasound Use by EMS Providers in Out-of-Hospital Cardiac Arrest." Prehosp Disaster Med 37(1): 39-44.
166. Kuttab, H. I., et al. (2021). "Prehospital Echocardiogram Use in Identifying Massive Pulmonary Embolism in Unidentified Respiratory Failure." Air Med J 40(1): 73-75.
167. LaDuke, M., et al. (2017). "Ultrasound Detection of Soft Tissue Abscesses Performed by Non-Physician U.S. Army Medical Providers Naïve to Diagnostic Sonography." Mil Med 182(3): e1825-e1830.
168. Lahham, S., et al. (2023). "Application of Point-of-care Ultrasound for Screening Climbers at High Altitude for Pulmonary B-lines." West J Emerg Med 24(2): 359-362.
169. Lahham, S., et al. (2015). "Prehospital assessment with ultrasound in emergencies-pause II." Academic Emergency Medicine 22(5): S227.
170. Lamorte, A., et al. (2016). "The Sierra Leone Ultrasound Rainbow4Africa Project (SLURP): an observational study of ultrasound effectiveness in developing countries." Crit Ultrasound J 8(1): 14.
171. Lapostolle, F. and T. Petrovic (2022). "[Prehospital ultrasound and cardiological emergencies]." Ann Cardiol Angeiol (Paris) 71(6): 345-349.
172. Lauridsen, S. V., et al. (2024). "Prehospital point-of-care ultrasound in ruptured abdominal aortic aneurysms-a retrospective cohort study." Acta Anaesthesiol Scand 68(5): 693-701.
173. Lema, P. C., et al. (2018). "Avoid the Goose! Paramedic Identification of Esophageal Intubation by Ultrasound." Prehosp Disaster Med 33(4): 406-410.
174. Lenz, T. J., et al. (2021). "Determining a Need for Point-of-Care Ultrasound in Helicopter Emergency Medical Services Transport." Air Med J 40(3): 175-178.
175. Leopold, S. J., et al. (2018). "Point-of-care lung ultrasound for the detection of pulmonary manifestations of malaria and sepsis: An observational study." PLoS One 13(12): e0204832.
176. Levine, A. C., et al. (2016). "Regional Anesthesia for Painful Injuries after Disasters (RAPID): study protocol for a randomized controlled trial." Trials 17(1): 542.
177. Limani, F., et al. (2021). "Diagnostic point-of-care ultrasound in medical inpatients at Queen Elizabeth Central Hospital, Malawi: an observational study of practice and evaluation of implementation." Trans R Soc Trop Med Hyg 115(8): 863-869.
178. Lipsitz, M., et al. (2022). "The State of Point-of-Care Teleultrasound Use for Educational Purposes: A Scoping Review." J Ultrasound Med 41(8): 1889-1906.
179. Lobo, M., et al. (2022). "Point of care prehospital ultrasound in Basic Emergency Services in Portugal." Health Sci Rep 5(5): e847.
180. Lochner, P., et al. (2015). "Ultrasonography of the Optic Nerve Sheath Diameter for Diagnosis and Monitoring of Acute Mountain Sickness: A Systematic Review." High Alt Med Biol 16(3): 195-203.
181. Luntsi, G., et al. (2022). "Routine Ultrasonography for Intensified Tuberculosis Case Finding in High Human Immunodeficiency Virus (HIV) and Tuberculosis (TB) Burdened Countries: A Proposed Frame Work." J Med Ultrasound 30(4): 245-250.
182. Marshall-Goebel, K., et al. (2019). "Assessment of Jugular Venous Blood Flow Stasis and Thrombosis During Spaceflight." JAMA Netw Open 2(11): e1915011.
183. Marshburn, T. H., et al. (2014). "New heights in ultrasound: first report of spinal ultrasound from the international space station." J Emerg Med 46(1): 61-70.
184. Marshburn, T. H., et al. (2004). "Goal-directed ultrasound in the detection of long-bone fractures." J Trauma 57(2): 329-332.
185. Martin, D. A., et al. (2022). "Role of Tele-ultrasound for Teaching Ultrasound-guided Nerve Blocks in the Emergency Department: A Case Series from Peru." Clin Pract Cases Emerg Med 6(3): 204-207.
186. Martin, D. S., et al. (2003). "Ultrasound in space." Ultrasound Med Biol 29(1): 1-12.
187. Martinet, C., et al. (2024). "Usefulness of an Ultrasound-guided Prehospital Care for SCUBA Diving Accidents." Mil Med.
188. Martins Barros, I. M., et al. (2021). "Accuracy and reliability of focused echocardiography in patients with Chagas disease from endemic areas: SaMi-Trop cohort study." PLoS One 16(11): e0258767.
189. Mason, R., et al. (2019). "Teaching Flight Nurses Ultrasonographic Evaluation of Esophageal Intubation and Pneumothorax." Air Med J 38(3): 195-197.
190. Maw, A. M., et al. (2019). "Stakeholder Perceptions of Point-of-Care Ultrasound Implementation in Resource-Limited Settings." Diagnostics (Basel) 9(4).
191. Mazmanyan, P., et al. (2020). "Introduction of point-of-care neonatal lung ultrasound in a developing country." Eur J Pediatr 179(7): 1131-1137.
192. Mazur, S. M., et al. (2007). "Use of point-of-care ultrasound by a critical care retrieval team." Emerg Med Australas 19(6): 547-552.
193. Mbanjumucyo, G. and P. C. Henwood (2016). "Focused assessment with sonography for HIV-associated tuberculosis (FASH) case series from a Rwandan district hospital." Afr J Emerg Med 6(4): 198-201.
194. McNeil, C. R., et al. (2009). "The accuracy of portable ultrasonography to diagnose fractures in an austere environment." Prehosp Emerg Care 13(1): 50-52.
195. Merlin, M. A., et al. (2020). "Out-of-Hospital Transesophageal Echocardiogram for Cardiac Arrest Resuscitation: The Initial Case." Prehosp Emerg Care 24(1): 90-93.
196. Micheller, D., et al. (2019). "Defining a Theory-Driven Ultrasound Curriculum for Prehospital Providers." Air Med J 38(4): 285-288.
197. Michels, G., et al. (2023). "[Recommendations for Education in Sonography in Prehospital Emergency Medicine (pPOCUS): Consensus paper of DGINA, DGAI, BAND, BV-ÄLRD, DGU, DIVI and DGIIN]." Anaesthesiologie 72(9): 654-661.
198. Modi, P., et al. (2016). "Accuracy of Inferior Vena Cava Ultrasound for Predicting Dehydration in Children with Acute Diarrhea in Resource-Limited Settings." PLoS One 11(1): e0146859.
199. Monti, J. D. and M. D. Perreault (2020). "Impact of a 4-hour Introductory eFAST Training Intervention Among Ultrasound-Naïve U.S. Military Medics." Mil Med 185(5-6): e601-e608.
200. Moore, K. A., et al. (2015). "Estimating Gestational Age in Late Presenters to Antenatal Care in a Resource-Limited Setting on the Thai-Myanmar Border." PLoS One 10(6): e0131025.
201. Morgan, A. R., et al. (2010). "Special operator level clinical ultrasound: an experience in application and training." J Spec Oper Med 10(2): 16-24.
202. Moser, A., et al. (2023). "From the Operating Room to the Cave: Ultrasound-Guided Locoregional Anesthesia in the Setting of Cave Rescue-A Description of 2 Cases." Wilderness Environ Med 34(4): 553-557.
203. Muhame, R. M., et al. (2021). "Cardiac point of care ultrasound in resource limited settings to manage children with congenital and acquired heart disease." Cardiol Young 31(10): 1651-1657.
204. Mulye, A., et al. (2023). "Virtual immediate feedback with POCUS in Belize." Front Digit Health 5: 1268905.
205. Murali, S., et al. (2022). "Helicopter Emergency Medical Services Ultrasound Use in the Diagnosis of Pericardial Effusion Due to Aortic Dissection." Air Med J 41(6): 566-569.
206. Muriuki, D., et al. (2024). "Focused cardiac ultrasound: Competency among pre-internship medical officers in diagnosing cardiac causes of dyspnea." J Clin Ultrasound 52(4): 426-434.
207. Murray, A., et al. (2020). "The Use of Point-of-Care Ultrasound to Accurately Measure Cardiac Output in Flight." Air Med J 39(3): 218-220.
208. Musolino, A. M., et al. (2024). "Pediatric ultrasound practice in Italy: an exploratory survey." Ital J Pediatr 50(1): 114.
209. Myers, M. A., et al. (2021). "Ultrasound at the Role 1: An Analysis of After-Action Reviews from the Prehospital Trauma Registry." Med J (Ft Sam Houst Tex)(Pb 8-21-07/08/09): 20-24.
210. Nacarapa, E., et al. (2022). "Extrapulmonary tuberculosis mortality according to clinical and point of care ultrasound features in Mozambique." Sci Rep 12(1): 16675.
211. Nadim, G., et al. (2021). "Prehospital emergency medical technicians can perform ultrasonography and blood analysis in prehospital evaluation of patients with chronic obstructive pulmonary disease: a feasibility study." BMC Health Serv Res 21(1): 290.
212. Nadimpalli, A., et al. (2019). "Feasibility of Training Clinical Officers in Point-of-Care Ultrasound for Pediatric Respiratory Diseases in Aweil, South Sudan." Am J Trop Med Hyg 101(3): 689-695.
213. Naeem, S., et al. (2023). "Feasibility and impact of a bespoke pre-hospital point of care ultrasound teaching and training programme at London's air ambulance service." Ultrasound 31(3): 230-235.
214. Naeem, S., et al. (2022). "A National Survey of Prehospital Care Services of United Kingdom for Use, Governance and Perception of Prehospital Point of Care Ultrasound." Pocus j 7(2): 232-238.
215. Nations, J. A. and R. F. Browning (2011). "Battlefield applications for handheld ultrasound." Ultrasound Q 27(3): 171-176.
216. Ndege, R., et al. (2023). "Ultrasononography in Managing Extrapulmonary Tuberculosis: A Randomized, Controlled, Parallel, Superiority, Open-Label Trial." Clin Infect Dis 76(6): 1013-1021.
217. Nelson, B. P. and K. Chason (2008). "Use of ultrasound by emergency medical services: a review." Int J Emerg Med 1(4): 253-259.
218. Nelson, B. P., et al. (2011). "Portable ultrasound for remote environments, part II: current indications." J Emerg Med 40(3): 313-321.
219. Nelson, B. P. and A. Sanghvi (2016). "Out of hospital point of care ultrasound: current use models and future directions." Eur J Trauma Emerg Surg 42(2): 139-150.
220. Neugebauer, F., et al. (2018). "[Point-of-Care Ultrasound: Teaching and Learning in Ifakara, Tanzania]." Praxis (Bern 1994) 107(23): 1279-1282.
221. Ngome, O. and M. Rohacek (2020). "Point-of-Care Ultrasound: A Useful Diagnostic Tool in Africa." Praxis (Bern 1994) 109(8): 608-614.
222. Nhat, P. T. H., et al. (2023). "Clinical benefit of AI-assisted lung ultrasound in a resource-limited intensive care unit." Crit Care 27(1): 257.
223. Nolting, L., et al. (2019). "Solar-Powered Point-of-Care Sonography: Our Himalayan Experience." J Ultrasound Med 38(9): 2477-2484.
224. O'Connor, L., et al. (2023). "A Novel Point-of-care Ultrasound Curriculum for Air Critical Care Personnel." West J Emerg Med 24(1): 30-37.
225. Paziana, K., et al. (2012). "Soft tissue foreign body removal technique using portable ultrasonography." Wilderness Environ Med 23(4): 343-348.
226. Pellegrini, J. A. S., et al. (2018). "Point-of-care ultrasonography in Brazilian intensive care units: a national survey." Ann Intensive Care 8(1): 50.
227. Perks, A. and P. Brendt (2024). "Pericardiocentesis for COVID-19 Associated Cardiac Tamponade Using a Central Venous Catheter in Rural Australia: A Case Report." Air Med J 43(1): 63-65.
228. Perrier, P., et al. (2020). "Usefulness of point-of-care ultrasound in military medical emergencies performed by young military medicine residents." BMJ Mil Health 166(4): 236-239.
229. Ploutz, M., et al. (2016). "Handheld echocardiographic screening for rheumatic heart disease by non-experts." Heart 102(1): 35-39.
230. Polan, D. L., et al. (2014). "Rapid internet-based review of point-of-care ultrasound studies at a remote hospital in Uganda." Academic Emergency Medicine 21(5): S324-S325.
231. Press, G. M., et al. (2013). "Evaluation of a training curriculum for prehospital trauma ultrasound." J Emerg Med 45(6): 856-864.
232. Prosen, G., et al. (2011). "Combination of lung ultrasound (a comet-tail sign) and N-terminal pro-brain natriuretic peptide in differentiating acute heart failure from chronic obstructive pulmonary disease and asthma as cause of acute dyspnea in prehospital emergency setting." Crit Care 15(2): R114.
233. Qi, X., et al. (2019). "Focused Assessment with Sonography in Trauma for Assessment of Injury in Military Settings: A Meta-analysis." Balkan Med J 37(1): 3-8.
234. Raees, M. Q., et al. (2024). "Optic nerve sheath diameter and its association with brain swelling in pediatric cerebral malaria: a retrospective study." Front Pediatr 12: 1295254.
235. Rahulkumar, H. H., et al. (2019). "Utility of Point-of-Care Ultrasound in Differentiating Causes of Shock in Resource-Limited Setup." J Emerg Trauma Shock 12(1): 10-17.
236. Raiten, J., et al. (2020). "Perioperative Point-of-Care Ultrasound and Transesophageal Echocardiography in Resource- Limited Settings-A Focus on Nepal and Bangladesh." J Cardiothorac Vasc Anesth 34(10): 2604-2610.
237. Remppis, J., et al. (2020). "Focused Assessment with Sonography for Urinary Schistosomiasis (FASUS)-pilot evaluation of a simple point-of-care ultrasound protocol and short training program for detecting urinary tract morbidity in highly endemic settings." Trans R Soc Trop Med Hyg 114(1): 38-48.
238. Renard, A., et al. (2019). "Is E-FAST possible and useful on the battlefield? A feasibility study during medical courses in hostile environment (MEDICHOS): preliminary results." J R Army Med Corps 165(5): 338-341.
239. Reynolds, T. A., et al. (2018). "Impact of point-of-care ultrasound on clinical decision-making at an urban emergency department in Tanzania." PLoS One 13(4): e0194774.
240. Rimbaut, E., et al. (2022). "Overview of current implementation and limitations of point-of-care ultrasound in the emergency department: a nationwide survey in Belgium." Acta Clin Belg 77(3): 631-639.
241. Rippey, J. C. and A. G. Royse (2009). "Ultrasound in trauma." Best Pract Res Clin Anaesthesiol 23(3): 343-362.
242. Robinson, A. E., et al. (2023). "Prehospital Ultrasound Diagnosis of Massive Pulmonary Embolism by Non-Physicians: A Case Series." Prehosp Emerg Care 27(6): 826-831.
243. Roline, C. E., et al. (2013). "Feasibility of bedside thoracic ultrasound in the helicopter emergency medical services setting." Air Med J 32(3): 153-157.
244. Rominger, A. H., et al. (2018). "The implementation of a longitudinal POCUS curriculum for physicians working at rural outpatient clinics in Chiapas, Mexico." Crit Ultrasound J 10(1): 19.
245. Ronaldson, J., et al. (2020). "Can non-physician advanced retrieval practitioners (ARP) acquire and interpret diagnostic views of the lungs with sufficient quality to aid in the diagnosis of pneumothorax in the pre-hospital and retrieval environment?" Scand J Trauma Resusc Emerg Med 28(1): 102.
246. Rooney, K. P., et al. (2016). "Pre-hospital assessment with ultrasound in emergencies: implementation in the field." World J Emerg Med 7(2): 117-123.
247. Ross, E. M., et al. (2015). "Operational Point-of-Care Ultrasound Review: Low-Cost Simulators and Resources for Advanced Prehospital Providers." J Spec Oper Med 15(1): 71-78.
248. Roy, S., et al. (2019). "Chemical Heat Packs as an Intervention to Prolong Ultrasound Battery Runtime." Wilderness Environ Med 30(2): 186-190.
249. Rozanski, T. A., et al. (2005). "Ultrasonography in a forward-deployed military hospital." Mil Med 170(2): 99-102.
250. Rozycki, G. S. and P. G. Newman (1999). "Surgeon-performed ultrasound for the assessment of abdominal injuries." Adv Surg 33: 243-259.
251. Rupp, J., et al. (2018). "Systematic Sonography for Detection of Occult Wounds in Trauma." J Ultrasound Med 37(6): 1539-1542.
252. Russell, T. C. and P. F. Crawford (2013). "Ultrasound in the austere environment: a review of the history, indications, and specifications." Mil Med 178(1): 21-28.
253. Sabatino, V., et al. (2020). "Point-of-care ultrasound (POCUS) in a remote area of Sierra Leone: impact on patient management and training program for community health officers." J Ultrasound 23(4): 521-527.
254. Salmon, M., et al. (2017). "Getting It Right the First Time: Defining Regionally Relevant Training Curricula and Provider Core Competencies for Point-of-Care Ultrasound Education on the African Continent." Ann Emerg Med 69(2): 218-226.
255. Savell, S. C., et al. (2021). "Military Use of Point of Care Ultrasound (POCUS)." J Spec Oper Med 21(2): 35-42.
256. Scharonow, M. and C. Weilbach (2018). "Prehospital point-of-care emergency ultrasound: a cohort study." Scand J Trauma Resusc Emerg Med 26(1): 49.
257. Schmidt, J., et al. (2022). "Training for Pediatric Cardiac and Pulmonary Point of Care Ultrasound in Eastern Uganda." Ultrasound Med Biol 48(12): 2461-2467.
258. Schoeneck, J. H., et al. (2021). "Paramedic-performed Prehospital Point-of-care Ultrasound for Patients with Undifferentiated Dyspnea: A Pilot Study." West J Emerg Med 22(3): 750-755.
259. Sedlakova, A., et al. (2020). "Prehospital ultrasound use among Canadian aeromedical service providers - A cross-sectional survey." Cjem 22(3): 338-341.
260. Sellon, E., et al. (2021). "Assessing the utility of ultrasound in the role 2 hospital setting." BMJ Mil Health 167(5): 323-326.
261. Sepulveda-Ortiz, V., et al. (2020). "The effectiveness of a longitudinal ultrasound curriculum for general pediatricians working in a Puerto Rican emergency department: a pilot study." Ultrasound J 12(1): 20.
262. Shaddock, L. and T. Smith (2022). "Potential for Use of Portable Ultrasound Devices in Rural and Remote Settings in Australia and Other Developed Countries: A Systematic Review." J Multidiscip Healthc 15: 605-625.
263. Shah, S., et al. (2008). "Development of an ultrasound training curriculum in a limited resource international setting: successes and challenges of ultrasound training in rural Rwanda." Int J Emerg Med 1(3): 193-196.
264. Shah, S. P., et al. (2016). "Focused cardiopulmonary ultrasound for assessment of dyspnea in a resource-limited setting." Crit Ultrasound J 8(1): 7.
265. Shari, C. R., et al. (2018). "Emergency centre diagnosis and treatment of purulent pericarditis: A case report from Tanzania." Afr J Emerg Med 8(1): 37-39.
266. Shaw, M. R., et al. (2023). "Guiding Emergency Treatment With Extended Focused Assessment With Sonography in Trauma by Emergency Responders (GET eFASTER)." Air Med J 42(1): 42-47.
267. Shokoohi, H., et al. (2018). "The utility of point-of-care ultrasound in targeted automobile ramming mass casualty (TARMAC) attacks." Am J Emerg Med 36(8): 1467-1471.
268. Shokoohi, H., et al. (2019). "Assessment of Point-of-Care Ultrasound Training for Clinical Educators in Malawi, Tanzania and Uganda." Ultrasound Med Biol 45(6): 1351-1357.
269. Shorter, M. and D. J. Macias (2012). "Portable handheld ultrasound in austere environments: use in the Haiti disaster." Prehosp Disaster Med 27(2): 172-177.
270. Shrestha, R., et al. (2020). "Evaluation of Interdisciplinary Emergency Ultrasound Workshop for Primary Care Physicians in Nepal." Open Access Emerg Med 12: 99-109.
271. Shumbusho, J. P., et al. (2020). "Accuracy of Resident-Performed Point-of-Care Lung Ultrasound Examinations Versus Chest Radiography in Pneumothorax Follow-up After Tube Thoracostomy in Rwanda." J Ultrasound Med 39(3): 499-506.
272. Siu, M., et al. (2023). "Impact of Telemedicine on Extended Focused Assessment With Sonography for Trauma Performance and Workload by Critical Care Transport Personnel." Air Med J 42(2): 105-109.
273. Smith, B., et al. (2024). "Prehospital Extended FAST Exams Improve Clinical Decision Making by Helicopter EMS Crews: A Retrospective Case Series." Prehosp Emerg Care 28(5): 727-734.
274. Smith, Z. A., et al. (2010). "FAST scanning in the developing world emergency department." S Afr Med J 100(2): 105-108.
275. Snaith, B., et al. (2011). "Emergency ultrasound in the prehospital setting: the impact of environment on examination outcomes." Emerg Med J 28(12): 1063-1065.
276. Snyder, R. and D. B. Brillhart (2023). "Pain Control and Point-of-Care Ultrasound: An Approach to Rib Fractures for the Austere Provider." J Spec Oper Med 23(3): 70-73.
277. Soták, M., et al. (2023). "The Role of Ultrasound Examination in the Differential Diagnosis of Cardiac Arrest in Prehospital Care: A Case Report." Prehosp Emerg Care 27(2): 275-277.
278. Spencer, J. K. and R. S. Adler (2008). "Utility of portable ultrasound in a community in Ghana." J Ultrasound Med 27(12): 1735-1743.
279. Stachura, M., et al. (2017). "Evaluation of a point-of-care ultrasound scan list in a resource-limited emergency centre in Addis Ababa Ethiopia." Afr J Emerg Med 7(3): 118-123.
280. Stanley, A., et al. (2017). "The Impact of Systematic Point-of-Care Ultrasound on Management of Patients in a Resource-Limited Setting." Am J Trop Med Hyg 96(2): 488-492.
281. Stephens, M. B., et al. (2000). "The maternal perspective on prenatal ultrasound." J Fam Pract 49(7): 601-604.
282. Stolz, L. A., et al. (2015). "Point-of-care ultrasound education for non-physician clinicians in a resource-limited emergency department." Trop Med Int Health 20(8): 1067-1072.
283. Stomeo, N., et al. (2023). "Role of emergency teleradiology in a mass motorcycle event: the experience of the 2021 International Six Days of Enduro (ISDE)." Emerg Radiol 30(6): 725-731.
284. Stralec, G., et al. (2024). "Is a Positive Prehospital FAST Associated with Severe Bleeding? A Multicenter Retrospective Study." Prehosp Emerg Care 28(4): 572-579.
285. Strnad, M., et al. (2016). "Bedside lung ultrasound for monitoring the effectiveness of prehospital treatment with continuous positive airway pressure in acute decompensated heart failure." Eur J Emerg Med 23(1): 50-55.
286. Stroffolini, G., et al. (2023). "Case Report: Role of Point-of-Care Ultrasound in the Diagnosis of Bacillary Angiomatosis." Am J Trop Med Hyg 109(1): 38-41.
287. Subedi, S., et al. (2023). "Three rare presentations of high-altitude pulmonary edema at a high-altitude clinic in the Everest region (4371 m): A case series." Clin Case Rep 11(4): e7236.
288. Sullivan, C., et al. (2024). "Acute Coronary Syndrome at Altitude: Diagnostic Dilemma on Aconcagua Using Point-of-Care Ultrasound." Wilderness Environ Med: 10806032241249128.
289. Sullivan, J. F., et al. (2021). "Utility of Point of Care Ultrasound in Humanitarian Assistance Missions." Mil Med 186(Suppl 1): 789-794.
290. Sutherland, A. I., et al. (2008). "Optic nerve sheath diameter, intracranial pressure and acute mountain sickness on Mount Everest: a longitudinal cohort study." Br J Sports Med 42(3): 183-188.
291. Suttels, V., et al. (2023). "Barriers and facilitators to implementation of point-of-care lung ultrasonography in a tertiary centre in Benin: a qualitative study among general physicians and pneumologists." BMJ Open 13(6): e070765.
292. Tafoya, C. A., et al. (2017). "Sustainable Resuscitation Ultrasound Education in a Low-Resource Environment: The Kumasi Experience." J Emerg Med 52(5): 723-730.
293. Terry, B., et al. (2019). "Rapid, remote education for point-of-care ultrasound among non-physician emergency care providers in a resource limited setting." Afr J Emerg Med 9(3): 140-144.
294. Thomas, O., et al. (2023). "Point-Of-Care Ultra-Sound (POCUS) for suspected surgical abdominal conditions in rural India-a mixed-method health needs assessment." PLOS Glob Public Health 3(1): e0001233.
295. Todorovich, S. M., et al. (2024). "3M™ Defib-Pads as a Reusable Alternative to Commercial Ultrasound Gel in Resource-Limited Settings." Wilderness Environ Med: 10806032241259938.
296. Toscano, M., et al. (2021). "Testing telediagnostic obstetric ultrasound in Peru: a new horizon in expanding access to prenatal ultrasound." BMC Pregnancy Childbirth 21(1): 328.
297. Toscano, M., et al. (2020). "Evaluating sensitivity and specificity of handheld point-of-care ultrasound testing for gynecologic pathology: a pilot study for use in low resource settings." BMC Med Imaging 20(1): 121.
298. Tran, T. T., et al. (2021). "Point-of-Care Ultrasound: Applications in Low- and Middle-Income Countries." Curr Anesthesiol Rep 11(1): 69-75.
299. Trovato, F. M., et al. (2016). "Thoracic ultrasound: An adjunctive and valuable imaging tool in emergency, resource-limited settings and for a sustainable monitoring of patients." World J Radiol 8(9): 775-784.
300. Umuhire, O. F., et al. (2019). "Impact of ultrasound on management for dyspnea presentations in a Rwandan emergency department." Ultrasound J 11(1): 18.
301. Van Hoving, D. J., et al. (2019). "Abdominal ultrasound for diagnosing abdominal tuberculosis or disseminated tuberculosis with abdominal involvement in HIV-positive individuals." Cochrane Database Syst Rev 9(9): Cd012777.
302. Vianen, N. J., et al. (2023). "Impact of Point-of-Care Ultrasound on Prehospital Decision Making by HEMS Physicians in Critically Ill and Injured Patients: A Prospective Cohort Study." Prehosp Disaster Med 38(4): 444-449.
303. Vicent, O., et al. (2024). "[Prehospital ultrasound in emergency medicine]." Anaesthesiologie 73(8): 502-510.
304. Volpicelli, G., et al. (2012). "International evidence-based recommendations for point-of-care lung ultrasound." Intensive Care Med 38(4): 577-591.
305. von Foerster, N., et al. (2024). "Prehospital Ultrasound: A Narrative Review." Prehosp Emerg Care 28(1): 1-13.
306. Vyas, A., et al. (2018). "Feasibility study of minimally trained medical students using the Rural Obstetrical Ultrasound Triage Exam (ROUTE) in rural Panama." World J Emerg Med 9(3): 216-222.
307. Wachira, J., et al. (2023). "A training program for obstetrics point-of-care ultrasound to 514 rural healthcare providers in Kenya." BMC Med Educ 23(1): 922.
308. Walcher, F., et al. (2002). "[Optimized management of polytraumatized patients by prehospital ultrasound]." Unfallchirurg 105(11): 986-994.
309. Wangmang, F. and R. Joseph (2020). "Left ventricular mass in a patient with severe heart failure." Afr J Emerg Med 10(4): 269-273.
310. Wanjiku, G. W., et al. (2024). "Impact of point-of-care ultrasound use on patient referral decisions in rural Kenya: a mixed methods study." BMC Health Serv Res 24(1): 212.
311. Waweru-Siika, W., et al. (2022). "Focused Cardiac Ultrasound Training for Non-cardiologists: An Overview and Recommendations for a Lower Middle-Income Country." Crit Care Clin 38(4): 827-837.
312. Weber, U., et al. (2018). "High altitude trekking after lung transplantation: a prospective study using lung ultrasound to detect comets tails for interstitial pulmonary edema in lung transplant recipients and healthy volunteers." Transpl Int 31(11): 1245-1253.
313. Weimer, J. M., et al. (2024). "Effectiveness of an ultrasound basic cancer training program through on-site training and virtual case discussions in rural Tanzania: a proof-of-concept study." Ecancermedicalscience 18: 1722.
314. Whitfield, D. A. and S. J. Portouw (2012). "Retinal detachment due to facial gunshot wound: the utility of ultrasonography in a medically austere environment." J Emerg Med 42(6): 678-681.
315. Wipplinger, F., et al. (2021). "Point-of-Care Ultrasound Diagnosis of Acute High Altitude Illness: A Case Report." Wilderness Environ Med 32(2): 204-209.
316. Woo, J. S., et al. (2014). "A futuristic vision of pocket ultrasound machines: watch this space." Australas J Ultrasound Med 17(3): 110-112.
317. Wray, J. N., et al. (2023). "Comparison of Commonly Carried Liquids Against Commercial Ultrasound Gel for Use in the Backcountry Setting." Wilderness Environ Med 34(2): 135-142.
318. Wydo, S. M., et al. (2016). "Portable ultrasound in disaster triage: a focused review." Eur J Trauma Emerg Surg 42(2): 151-159.
319. Yao, M., et al. (2020). "Demonstration of a Longitudinal Medical Education Model (LMEM) Model to Teach Point-of-Care Ultrasound in Resource-Limited Settings." Pocus j 5(1): 20-25.
320. Yates, J. G. and D. Baylous (2017). "Aeromedical Ultrasound: The Evaluation of Point-of-care Ultrasound During Helicopter Transport." Air Med J 36(3): 110-115.
321. Yates, J. G. and D. Baylous (2022). "Air Medical Ultrasound: Looking Back to See What We Have Learned for the Future." Air Med J 41(6): 536-541.
322. Zadel, S., et al. (2015). "Point of care ultrasound for orotracheal tube placement assessment in out-of hospital setting." Resuscitation 87: 1-6.
323. Zanatta, M., et al. (2020). "Ultrasound-Guided Chest Compressions in Out-of-Hospital Cardiac Arrests." J Emerg Med 59(6): e225-e233.
324. Zhou, M., et al. (2023). "Integrated microdroplet array platform with temperature controller and micro-stirring for ultra-fast SARS-CoV-2 detection." Biosens Bioelectron 220: 114903.
