# Supplementary material for: Point-of-care ultrasound use in austere environments: A scoping review
Source: PLoS One. 2024 Dec 5;19(12):e0312017. doi: 10.1371/journal.pone.0312017 (PMC11620461; doi:10.1371/journal.pone.0312017)
Supplement: S4 Table — (DOCX) [file pone.0312017.s006.docx]

| **Supplementary table 4: Austere ultrasound excluded studies** | | |
| --- | --- | --- |
| **Number** | **Excluded studies** | **Reason for exclusion** |
| 1. | Ultrasound Guidelines: Emergency, Point-of-Care and Clinical Ultrasound Guidelines in Medicine. Ann Emerg Med, 2017. 69(5): p. e27-e54. | No abstract |
| 2. | Global burden of chronic respiratory diseases and risk factors, 1990-2019: an update from the Global Burden of Disease Study 2019. EClinicalMedicine, 2023. 59: p. 101936. | Not point-of-care ultrasound (POCUS) |
| 3. | Abraham, S.V., et al., Indigenously Developed Ultrasound Phantom Model versus a Commercially Available Training Model: Randomized Double-blinded Study to Assess Its Utility to Teach Ultrasound Guided Vascular Access in a Controlled Setting. J Med Ultrasound, 2022. 30(1): p. 11-19. | Not involving healthcare workers |
| 4. | Abrams, E.R., et al., Point-of-Care Ultrasound in the Evaluation of COVID-19. J Emerg Med, 2020. 59(3): p. 403-408. | Not in austere environment |
| 5. | Abuhamad, A., et al., Standardized Six-Step Approach to the Performance of the Focused Basic Obstetric Ultrasound Examination. Am J Perinatol, 2016. 33(1): p. 90-8. | Curriculum development |
| 6. | Acar, Y., et al., 12th WINFOCUS world congress on ultrasound in emergency and critical care. Crit Ultrasound J, 2016. 8(Suppl 1): p. 12. | Curriculum development |
| 7. | Addepalli, A., et al., Point-of-Care Ultrasound Diagnosis of Tetralogy of Fallot Causing Cyanosis: A Case Report. Clin Pract Cases Emerg Med, 2022. 6(4): p. 280-283. | Not in austere environment |
| 8. | Adenekan, A.T., et al., Feasibility study for a randomized clinical trial of bupivacaine, lidocaine with adrenaline, or placebo wound infiltration to reduce postoperative pain after laparoscopic cholecystectomy. BJS Open, 2019. 3(4): p. 453-460. | Not POCUS |
| 9. | Al Deeb, M., et al., Point-of-care ultrasonography for the diagnosis of acute cardiogenic pulmonary edema in patients presenting with acute dyspnea: a systematic review and meta-analysis. Acad Emerg Med, 2014. 21(8): p. 843-52. | Not in austere environment |
| 10. | Alafeef, M., et al., RNA-extraction-free nano-amplified colorimetric test for point-of-care clinical diagnosis of COVID-19. Nat Protoc, 2021. 16(6): p. 3141-3162. | Not POCUS |
| 11. | Alarhayem, A.Q., et al., Impact of time to repair on outcomes in patients with lower extremity arterial injuries. J Vasc Surg, 2019. 69(5): p. 1519-1523. | Not POCUS |
| 12. | Alenazi, A. and A. Alshibani, Confirmatory methods for endotracheal tube placement in out-of-hospital settings: A systematic review of the literature. Heliyon, 2024. 10(7): p. e28479. | Not POCUS |
| 13. | Allen, B., et al., Scoping review of literature on B-mode cranial ultrasonography to detect intracranial hemorrhage. Critical Care Medicine, 2022. 50(1 SUPPL): p. 409. | Not in austere environment |
| 14. | Alqhtani, N.R., et al., Temporomandibular Joint Space Dimensions among Saudi Patients with Temporomandibular Disorders: MRI-Based Retrospective Study. Int J Clin Pract, 2022. 2022: p. 5846255. | Not POCUS |
| 15. | Alqurashi, N., et al., Head Injury Evaluation and Ambulance Diagnosis (HOME) Study protocol: a feasibility study assessing the implementation of the Canadian CT Head Rule in the prehospital setting. BMJ Open, 2024. 14(6): p. e077191. | Not POCUS |
| 16. | Alshahrani, A.M., et al., Point of Care Clinical Risk Score to Improve the Negative Diagnostic Utility of an Agatston Score of Zero: Averting the Need for Coronary Computed Tomography Angiography. Circ Cardiovasc Imaging, 2019. 12(9): p. e008737. | Not POCUS |
| 17. | Altaf, A., et al., Applications, limitations and advancements of ultra-low-field magnetic resonance imaging: A scoping review. Surg Neurol Int, 2024. 15: p. 218. | Not POCUS |
| 18. | An, R., et al., Emerging point-of-care technologies for anemia detection. Lab Chip, 2021. 21(10): p. 1843-1865. | Not POCUS |
| 19. | Anderson, K.L., et al., Point-of-care ultrasound diagnoses acute decompensated heart failure in the ED regardless of examination findings. Am J Emerg Med, 2014. 32(4): p. 385-8. | Not in austere environment |
| 20. | Anholm, J.D., et al., Radiographic evidence of interstitial pulmonary edema after exercise at altitude. J Appl Physiol (1985), 1999. 86(2): p. 503-9. | Not POCUS |
| 21. | Annigeri, S., et al., Utility of anthropometric measures to identify small for gestational age newborns: A study from Eastern India. J Family Med Prim Care, 2022. 11(6): p. 3125-3132. | Not POCUS |
| 22. | Arnold, T.C., et al., Low-field MRI: Clinical promise and challenges. J Magn Reson Imaging, 2023. 57(1): p. 25-44. | Not POCUS |
| 23. | Astner-Rohracher, A., et al., Development and Validation of the 5-SENSE Score to Predict Focality of the Seizure-Onset Zone as Assessed by Stereoelectroencephalography. JAMA Neurol, 2022. 79(1): p. 70-79. | Not POCUS |
| 24. | Ataş, E., et al., Evaluation of children with lympadenopathy. Turk Pediatri Ars, 2014. 49(1): p. 30-5. | Not POCUS |
| 25. | Auffret, V., et al., Efficacy of pre-hospital use of glycoprotein IIb/IIIa inhibitors in ST-segment elevation myocardial infarction before mechanical reperfusion in a rapid-transfer network (from the Acute Myocardial Infarction Registry of Brittany). Am J Cardiol, 2014. 114(2): p. 214-23. | Not POCUS |
| 26. | Augustine, R., et al., Loop-Mediated Isothermal Amplification (LAMP): A Rapid, Sensitive, Specific, and Cost-Effective Point-of-Care Test for Coronaviruses in the Context of COVID-19 Pandemic. Biology (Basel), 2020. 9(8). | Not POCUS |
| 27. | Aujla, S., et al., Classification of lung pathologies in neonates using dual-tree complex wavelet transform. Biomed Eng Online, 2023. 22(1): p. 115. | Not POCUS |
| 28. | Austad, G.T. and J.B. Higgs, Occult knee effusions in the intensive care environment identified by ultrasound: a new third space? J Clin Rheumatol, 2009. 15(8): p. 408-10. | Not in austere enrivonment |
| 29. | Babin, B.M., et al., Chemiluminescent Protease Probe for Rapid, Sensitive, and Inexpensive Detection of Live Mycobacterium tuberculosis. ACS Cent Sci, 2021. 7(5): p. 803-814. | Not POCUS |
| 30. | Baday, M., et al., Integrating Cell Phone Imaging with Magnetic Levitation (i-LEV) for Label-Free Blood Analysis at the Point-of-Living. Small, 2016. 12(9): p. 1222-1229. | Not POCUS |
| 31. | Bagayoko, C.O., et al., Medical and economic benefits of telehealth in low- and middle-income countries: results of a study in four district hospitals in Mali. BMC Health Serv Res, 2014. 14 Suppl 1(Suppl 1): p. S9. | Not POCUS |
| 32. | Bagheri-Hariri, S., et al., The impact of using RUSH protocol for diagnosing the type of unknown shock in the emergency department. Emerg Radiol, 2015. 22(5): p. 517-20. | Not in austere environment |
| 33. | Bakeer, N., et al., Haemophilic arthropathy: Diagnosis, management, and aging patient considerations. Haemophilia, 2024. 30 Suppl 3: p. 120-127. | Not POCUS |
| 34. | Barron, M.R., et al., Mobile forward-looking infrared technology allows rapid assessment of resuscitative endovascular balloon occlusion of the aorta in hemorrhage and blackout conditions. J Trauma Acute Care Surg, 2018. 85(1): p. 25-32. | Not POCUS |
| 35. | Barros, L.M., et al., Pragmatic Recommendations for Identification and Triage of Patients with COVID-19 in Low- and Middle-Income Countries. Am J Trop Med Hyg, 2021. 104(3_Suppl): p. 3-11. | Not POCUS |
| 36. | Bateman, R.M., et al., 36th International Symposium on Intensive Care and Emergency Medicine : Brussels, Belgium. 15-18 March 2016. Crit Care, 2016. 20(Suppl 2): p. 94. | Not involving healthcare workers |
| 37. | Bazarian, J.J., et al., Effects of Physical Exertion on Early Changes in Blood-Based Brain Biomarkers: Implications for the Acute Point of Care Diagnosis of Concussion. J Neurotrauma, 2023. 40(7-8): p. 693-705. | Not POCUS |
| 38. | Beam, G., et al., Point-of-Care Ultrasound Findings in a Case of Orbital Cellulitis: A Case Report. J Emerg Med, 2021. 61(2): p. 157-160. | Not in austere environment |
| 39. | Bedard, N., et al., Emerging roles for multimodal optical imaging in early cancer detection: a global challenge. Technol Cancer Res Treat, 2010. 9(2): p. 211-7. | Not POCUS |
| 40. | Benov, A., et al., Military medical research in the IDF: an array of fields and interests. Isr Med Assoc J, 2022. 24(9): p. 557-558. | Not POCUS |
| 41. | Benzon, H.T., et al., Use of corticosteroids for adult chronic pain interventions: sympathetic and peripheral nerve blocks, trigger point injections - guidelines from the American Society of Regional Anesthesia and Pain Medicine, the American Academy of Pain Medicine, the American Society of Interventional Pain Physicians, the International Pain and Spine Intervention Society, and the North American Spine Society. Reg Anesth Pain Med, 2024. | Not POCUS |
| 42. | Beygui, F., Pre-hospital care: a critical phase of the acute cardiovascular care management process. Eur Heart J, 2014. 35(30): p. 1984-5. | Not POCUS |
| 43. | Bhat, S.S., et al., Low-Field MRI of Stroke: Challenges and Opportunities. J Magn Reson Imaging, 2021. 54(2): p. 372-390. | Not POCUS |
| 44. | Blair, P.W., et al., Do worsening lung ultrasound scans identify severe COVID-19 trajectories? Front Med (Lausanne), 2022. 9: p. 1021929. | Not in austere environment |
| 45. | Blair, P.W., et al., Point-of-Care Lung Ultrasound Predicts Severe Disease and Death Due to COVID-19: A Prospective Cohort Study. Crit Care Explor, 2022. 4(8): p. e0732. | Not in austere environment |
| 46. | Blazic, I., et al., The use of lung ultrasound in COVID-19. ERJ Open Res, 2023. 9(1). | Not in austere environment |
| 47. | Bloom, A.S., J. Auten, and J.M. Schofer, Point-of-care Ultrasound for the Diagnosis of a "Ping Pong" Skull Fracture. Clin Pract Cases Emerg Med, 2018. 2(1): p. 99-100. | Not in austere environment |
| 48. | Bombah, F.M., et al., Focus on interlocking intramedullary nailing without fluoroscopy in resource-limited settings: strategies, outcomes, and outlook. Int Orthop, 2022. 46(1): p. 115-124. | Not POCUS |
| 49. | Bonnevialle, P., et al., [Traumatic knee dislocation with popliteal vascular disruption: retrospective study of 14 cases]. Rev Chir Orthop Reparatrice Appar Mot, 2006. 92(8): p. 768-77. | Not POCUS |
| 50. | Boppart, S.A. and R. Richards-Kortum, Point-of-care and point-of-procedure optical imaging technologies for primary care and global health. Sci Transl Med, 2014. 6(253): p. 253rv2. | Not POCUS |
| 51. | Bøtker, M.T., et al., Implementing point-of-care ultrasonography of the heart and lungs in an anesthesia department. Acta Anaesthesiol Scand, 2017. 61(2): p. 156-165. | Not austere environment |
| 52. | Bowra, J., et al., Sounding out the future of ultrasound education. Ultrasound, 2015. 23(1): p. 48-52. | Curriculum development |
| 53. | Boyko, E.V. and D.S. Mal'tsev, [En face' optical coherence tomography guided focal navigated laser photocoagulation]. Vestn Oftalmol, 2016. 132(3): p. 56-60. | Not POCUS |
| 54. | Brattain, L.J., et al., AI-Enabled, Ultrasound-Guided Handheld Robotic Device for Femoral Vascular Access. Biosensors (Basel), 2021. 11(12). | Not POCUS |
| 55. | Brenes, D., et al., Multi-task network for automated analysis of high-resolution endomicroscopy images to detect cervical precancer and cancer. Comput Med Imaging Graph, 2022. 97: p. 102052. | Not POCUS |
| 56. | Brzezinski, R.Y., et al., Automated processing of thermal imaging to detect COVID-19. Sci Rep, 2021. 11(1): p. 17489. | Not POCUS |
| 57. | Budhathoki-Uprety, J., et al., Synthetic molecular recognition nanosensor paint for microalbuminuria. Nat Commun, 2019. 10(1): p. 3605. | Not POCUS |
| 58. | Buendia, R., et al., Bioimpedance technology for detection of thoracic injury. Physiol Meas, 2017. 38(11): p. 2000-2014. | Not POCUS |
| 59. | Bunt, C.W., et al., Point-of-Care Estimated Radiation Exposure and Imaging Guidelines Can Reduce Pediatric Radiation Burden. J Am Board Fam Med, 2015. 28(3): p. 343-50. | Not POCUS |
| 60. | Busche, C., H.J. Busch, and G. Michels, [Point-of-Care Sonography in Emergency and Intensive Care Medicine]. Dtsch Med Wochenschr, 2018. 143(3): p. 161-164. | Not in austere environment |
| 61. | Campbell, K., et al., Damage control resuscitation and surgery for indigenous combat casualties: a prospective observational study. BMJ Mil Health, 2021. 167(1): p. 18-22. | Not POCUS |
| 62. | Canty, D., et al., Point-of-care ultrasound for deep venous thrombosis of the lower limb. Australas J Ultrasound Med, 2020. 23(2): p. 111-120. | Not in austere environment |
| 63. | Caroselli, C., et al., A Modified Corona Score Using Lung Ultrasound to Identify COVID-19 Patients. Diagnostics (Basel), 2023. 14(1). | Not in austere environment |
| 64. | Chang, M., et al., Optimal Image Gain Intensity of Point-of-care Ultrasound when Screening for Ocular Abnormalities in the Emergency Department. West J Emerg Med, 2023. 24(3): p. 622-628. | Not austere environment |
| 65. | Chao, J.H., et al., Circular right upper quadrant mass, not intussusception. Pediatr Emerg Care, 2015. 31(5): p. 384-7. | Not in austere environment |
| 66. | Chen, J., et al., Lateral Ventricular Volume Asymmetry Predicts Poor Outcome After Spontaneous Intracerebral Hemorrhage. World Neurosurg, 2018. 110: p. e958-e964. | Not POCUS |
| 67. | Chiavaras, M.M., et al., IMpact of Platelet Rich plasma OVer alternative therapies in patients with lateral Epicondylitis (IMPROVE): protocol for a multicenter randomized controlled study: a multicenter, randomized trial comparing autologous platelet-rich plasma, autologous whole blood, dry needle tendon fenestration, and physical therapy exercises alone on pain and quality of life in patients with lateral epicondylitis. Acad Radiol, 2014. 21(9): p. 1144-55. | Not POCUS |
| 68. | Christensen, H.M., et al., Patients' perspectives on point-of-care diagnostics and treatment by emergency medical technicians in acute COPD exacerbations: A qualitative study. Scand J Trauma Resusc Emerg Med, 2022. 30(1): p. 11. | Not POCUS |
| 69. | Chughtai, T., et al., Trauma intensive care unit (TICU) at Hamad General Hospital. Qatar Med J, 2019. 2019(2): p. 5. | Not POCUS |
| 70. | Chun, T.H., Multicenter pediatric emergency medicine research and Rhode Island. R I Med J (2013), 2014. 97(1): p. 35-9. | Not in austere environment |
| 71. | Cisco, G., et al., Cost-effectiveness analysis of procalcitonin and lung ultrasonography guided antibiotic prescriptions in primary care. Eur J Health Econ, 2024. | Not in austere environment |
| 72. | Coccolini, F., et al., Duodeno-pancreatic and extrahepatic biliary tree trauma: WSES-AAST guidelines. World J Emerg Surg, 2019. 14: p. 56. | Not POCUS |
| 73. | Cohen, S.P., et al., Fluoroscopically Guided vs Landmark-Guided Sacroiliac Joint Injections: A Randomized Controlled Study. Mayo Clin Proc, 2019. 94(4): p. 628-642. | Not POCUS |
| 74. | Cook, D.L., et al., Point-of-Care Ultrasound Use in Nephrology: A Survey of Nephrology Program Directors, Fellows, and Fellowship Graduates. Kidney Med, 2023. 5(4): p. 100601. | Not in austere environment |
| 75. | Corbett, M., et al., Point-of-care creatinine tests to assess kidney function for outpatients requiring contrast-enhanced CT imaging: systematic reviews and economic evaluation. Health Technol Assess, 2020. 24(39): p. 1-248. | Not POCUS |
| 76. | Coulibaly, M., et al., First Malian series of surgery for rheumatic valve disease: opening of the centre, clinical features and peri-operative realities. Cardiovasc J Afr, 2022. 33(2): p. 79-83. | Not POCUS |
| 77. | Crisp, J.D., Portable ultrasound empowers Special Forces medics. J Spec Oper Med, 2010. 10(1): p. 59-62. | No abstract |
| 78. | D'Costa, C., et al., Differential sensitivity to hypoxia enables shape-based classification of sickle cell disease and trait blood samples at point of care. Bioeng Transl Med, 2024. 9(4): p. e10643. | Not POCUS |
| 79. | Dagain, A., et al., Management of War-Related Ballistic Craniocerebral Injuries in a French Role 3 Hospital During the Afghan Campaign. World Neurosurg, 2017. 102: p. 6-12. | Not POCUS |
| 80. | Dahl, B.P., et al., Is portable ultrasonography accurate in the evaluation of Schanz pin placement during extremity fracture fixation in austere environments? Am J Disaster Med, 2013. 8(2): p. 91-6. | Not involving healthcare workers |
| 81. | Dainton, C., N. Shah, and C.H. Chu, Prevalence of Portable Point of Care Tests Used on Medical Service Trips in Latin America and the Caribbean. Ann Glob Health, 2018. 84(4): p. 736-742. | Not POCUS |
| 82. | Damjanovic, D., et al., An easy-to-build, low-budget point-of-care ultrasound simulator: from Linux to a web-based solution. Crit Ultrasound J, 2017. 9(1): p. 4. | Not involving healthcare workers |
| 83. | Dang, H., et al., Statistical reconstruction for cone-beam CT with a post-artifact-correction noise model: application to high-quality head imaging. Phys Med Biol, 2015. 60(16): p. 6153-75. | Not POCUS |
| 84. | de Assis, V., et al., Resuscitation of traumatic maternal cardiac arrest: A case report and summary of recommendations from Obstetric Life Support™. Trauma Case Rep, 2023. 44: p. 100800. | Not POCUS |
| 85. | Deng, C., et al., Small lung lesions invisible under fluoroscopy are located accurately by three-dimensional localization technique on chest wall surface and performed bronchoscopy procedures to increase diagnostic yields. BMC Pulm Med, 2016. 16(1): p. 166. | Not POCUS |
| 86. | Deng, R., et al., Smartphone-based microplate reader for high-throughput quantitation of disease markers in serum. Analyst, 2023. 148(4): p. 735-741. | Not POCUS |
| 87. | Devoto, C., et al., Plasma phosphorylated tau181 as a biomarker of mild traumatic brain injury: findings from THINC and NCAA-DoD CARE Consortium prospective cohorts. Front Neurol, 2023. 14: p. 1202967. | Not POCUS |
| 88. | Doll, D., et al., [Blunt trauma with bullet-proof vests. Skin lesions are no reliable predictor of injury severity]. Chirurg, 2009. 80(4): p. 348-51. | Not POCUS |
| 89. | Donaldson, R.I., et al., Development of a Novel Epidural Hemorrhage Model in Swine. Mil Med, 2023. 188(1-2): p. 20-26. | Not POCUS |
| 90. | Ebinger, M., et al., Association Between Dispatch of Mobile Stroke Units and Functional Outcomes Among Patients With Acute Ischemic Stroke in Berlin. Jama, 2021. 325(5): p. 454-466. | Not POCUS |
| 91. | Einvik, S., A.J. Kruger, and S.E. Gisvold, Pediatric hypothermic submersion incident - should we do chest compressions on a beating heart? Scand J Trauma Resusc Emerg Med, 2020. 28(1): p. 85. | Not POCUS |
| 92. | El Maghraoui, A., et al., Vertebral fractures and abdominal aortic calcification in postmenopausal women. A cohort study. Bone, 2013. 56(1): p. 213-9. | Not POCUS |
| 93. | El Saghir, N.S., Responding to the challenges of breast cancer in egypt and other arab countries. J Egypt Natl Canc Inst, 2008. 20(4): p. 309-12. | Not POCUS |
| 94. | Elliott, B.P., et al., Confidence and Utilization are Poorly Associated with Point-of-Care Ultrasound Competency among Internal Medicine Trainees. Mil Med, 2023. 188(Suppl 6): p. 316-321. | Not in austere environment |
| 95. | Ericsson, A.L., [Scholarship report of a 1982 study trip in western and middle Turkey]. Jordemodern, 1982. 95(10): p. 337-40. | Not POCUS |
| 96. | Espinoza, A.V., et al., PreSSUB II: The prehospital stroke study at the Universitair Ziekenhuis Brussel II. J Transl Int Med, 2015. 3(2): p. 57-63. | Not POCUS |
| 97. | Farkash, U., et al., Preliminary experience with postmortem computed tomography in military penetrating trauma. J Trauma, 2000. 48(2): p. 303-8; discussion 308-9. | Not POCUS |
| 98. | Fassbender, K., et al., Mobile stroke units for prehospital thrombolysis, triage, and beyond: benefits and challenges. Lancet Neurol, 2017. 16(3): p. 227-237. | Not POCUS |
| 99. | Fedor, P.J., et al., Major Trauma Outside a Trauma Center: Prehospital, Emergency Department, and Retrieval Considerations. Emerg Med Clin North Am, 2018. 36(1): p. 203-218. | Not POCUS |
| 100. | Foss, K.T., et al., Developing an emergency ultrasound app - a collaborative project between clinicians from different universities. Scand J Trauma Resusc Emerg Med, 2015. 23: p. 47. | Not in austere environment |
| 101. | Fox, C.J., B. Patel, and W.D. Clouse, Update on wartime vascular injury. Perspect Vasc Surg Endovasc Ther, 2011. 23(1): p. 13-25. | Not POCUS |
| 102. | Fredricks, T.R. and J.S. Benseler, Aortic Root Aneurism Found in a 42-Year-Old Epitomizes the Importance of Auscultation in Routine Exams. Aerosp Med Hum Perform, 2016. 87(5): p. 487-92. | Not POCUS |
| 103. | Frija, G., et al., A paradigm shift in point-of-care imaging in low-income and middle-income countries. EClinicalMedicine, 2023. 62: p. 102114. | Not POCUS |
| 104. | Frudd, K., et al., Diagnostic circulating biomarkers to detect vision-threatening diabetic retinopathy: Potential screening tool of the future? Acta Ophthalmol, 2022. 100(3): p. e648-e668. | Not POCUS |
| 105. | Fukushima, Y., et al., A Review and Proposed Rationale for the use of Ultrasonography as a Diagnostic Modality in the Identification of Bone Stress Injuries. J Ultrasound Med, 2018. 37(10): p. 2297-2307. | Not in austere environment |
| 106. | Gaarder, C., A. Holtan, and P.A. Naess, Prehospital point-of-care monitoring and goal-directed therapy: Does it make a difference? J Trauma Acute Care Surg, 2015. 78(6 Suppl 1): p. S60-4. | Not POCUS |
| 107. | Galarza, L., A. Wong, and M. Malbrain, The state of critical care ultrasound training in Europe: A survey of trainers and a comparison of available accreditation programmes. Anaesthesiol Intensive Ther, 2017. 49(5): p. 382-386. | Curriculum development |
| 108. | Gammie, J.S., et al., Safety and performance of a novel transventricular beating heart mitral valve repair system: 1-year outcomes. Eur J Cardiothorac Surg, 2021. 59(1): p. 199-206. | Not POCUS |
| 109. | Ganchi, F.A. and T.C. Hardcastle, Role of Point-of-Care Diagnostics in Lower- and Middle-Income Countries and Austere Environments. Diagnostics (Basel), 2023. 13(11). | Not POCUS |
| 110. | Gandhi, D., et al., Current role of imaging in COVID-19 infection with recent recommendations of point of care ultrasound in the contagion: a narrative review. Ann Transl Med, 2020. 8(17): p. 1094. | Not in austere environment |
| 111. | Geeraedts, L.M., Jr., et al., Exsanguination in trauma: A review of diagnostics and treatment options. Injury, 2009. 40(1): p. 11-20. | Not POCUS |
| 112. | Geis, R.N., et al., Novel Internal Medicine Residency Ultrasound Curriculum Led by Critical Care and Emergency Medicine Staff. Mil Med, 2023. 188(5-6): p. e936-e941. | Curriculum development |
| 113. | Geisler, F., et al., Prospective collection of blood plasma samples to identify potential biomarkers for the prehospital stroke diagnosis (ProGrEss-Bio): study protocol for a multicenter prospective observational study. Front Neurol, 2023. 14: p. 1201130. | Not POCUS |
| 114. | Ghosh, A., J. Mukherjee, and N. Chakravorty, A Low-Cost Test for Anemia Using an Artificial Neural Network. Comput Methods Programs Biomed, 2023. 229: p. 107251. | Not POCUS |
| 115. | Glaser, J.J., et al., Bridging the gap: Hybrid cardiac echo in the critically ill. J Trauma Acute Care Surg, 2016. 81(5 Suppl 2 Proceedings of the 2015 Military Health System Research Symposium): p. S157-s161. | Not in austere environment |
| 116. | Gleeson, T. and D. Blehar, Point-of-Care Ultrasound in Trauma. Semin Ultrasound CT MR, 2018. 39(4): p. 374-383. | Not in austere environment |
| 117. | Glezeva, N., et al., Heart failure in sub-Saharan Africa: review of the aetiology of heart failure and the role of point-of-care biomarker diagnostics. Trop Med Int Health, 2015. 20(5): p. 581-588. | Not POCUS |
| 118. | Goldstein, J., et al., Determinants for scalable adoption of autonomous AI in the detection of diabetic eye disease in diverse practice types: key best practices learned through collection of real-world data. Front Digit Health, 2023. 5: p. 1004130. | Not POCUS |
| 119. | Golightly, Y.M., et al., Association of Traumatic Knee Injury With Radiographic Evidence of Knee Osteoarthritis in Military Officers. Arthritis Care Res (Hoboken), 2023. 75(8): p. 1744-1751. | Not POCUS |
| 120. | Gottlieb, M., B. Long, and A. Koyfman, Evaluation and Management of Aortic Stenosis for the Emergency Clinician: An Evidence-Based Review of the Literature. J Emerg Med, 2018. 55(1): p. 34-41. | Not POCUS |
| 121. | Grant, B.D., et al., High-resolution microendoscopy: a point-of-care diagnostic for cervical dysplasia in low-resource settings. Eur J Cancer Prev, 2017. 26(1): p. 63-70. | Not POCUS |
| 122. | Gunther, R.S., K.P. Banks, and N.E. McWhorter, Universal Fasting Glucose Screening Before Gastric Emptying Scintigraphy and the High Prevalence of Undiagnosed Diabetes and Prediabetes. J Nucl Med Technol, 2024. 52(1): p. 52-54. | Not POCUS |
| 123. | Gurkan, U.A., et al., Miniaturized lensless imaging systems for cell and microorganism visualization in point-of-care testing. Biotechnol J, 2011. 6(2): p. 138-49. | Not POCUS |
| 124. | Guttikonda, S.N.R. and K. Vadapalli, Approach to undifferentiated dyspnea in emergency department: aids in rapid clinical decision-making. Int J Emerg Med, 2018. 11(1): p. 21. | Not in austere environment |
| 125. | Habib, P., et al., Point-of-Care Ultrasound to Detect Acute Large Vessel Occlusions in Stroke Patients: A Proof-of-Concept Study. Can J Neurol Sci, 2023. 50(5): p. 656-661. | Not in austere environment |
| 126. | Hai, P.D., et al., A case report of primary aortoduodenal fistula: A forgotten cause of gastrointestinal bleeding. Radiol Case Rep, 2023. 18(4): p. 1556-1559. | Not POCUS |
| 127. | Hailemariam, T., et al., Utility of chest imaging in the diagnosis and management of patients with visceral leishmaniasis: A systematic review. SAGE Open Med, 2023. 11: p. 20503121231177812. | Not POCUS |
| 128. | Hailong, Y., et al., Computer analysis of the safety of using three different pedicular screw insertion points in the lumbar spine in the Chinese population. Eur Spine J, 2007. 16(5): p. 619-23. | Not POCUS |
| 129. | Hall, J.W.W., et al., Point-of-Care Ultrasound in Family Medicine Residencies 5-Year Update: A CERA Study. Fam Med, 2020. 52(7): p. 505-511. | Not in austere environment |
| 130. | Hamdy, F.C., et al., Active monitoring, radical prostatectomy and radical radiotherapy in PSA-detected clinically localised prostate cancer: the ProtecT three-arm RCT. Health Technol Assess, 2020. 24(37): p. 1-176. | Not POCUS |
| 131. | Han, D., et al., Association of Plaque Location and Vessel Geometry Determined by Coronary Computed Tomographic Angiography With Future Acute Coronary Syndrome-Causing Culprit Lesions. JAMA Cardiol, 2022. 7(3): p. 309-319. | Not POCUS |
| 132. | Harris, N.S., Response to Lipman et al. re: "Ultrasound in Austere Environments" (DOI: 10.1089/ham.2019.0102). High Alt Med Biol, 2019. 20(4): p. 441. | No abstract |
| 133. | Hayat, A., et al., Diaphragmatic Excursion: Does it Predict Successful Weaning from Mechanical Ventilation? J Coll Physicians Surg Pak, 2017. 27(12): p. 743-746. | Not in austere environment |
| 134. | He, Y., et al., Use of 2.1 MHz MRI scanner for brain imaging and its preliminary results in stroke. J Magn Reson, 2020. 319: p. 106829. | Not POCUS |
| 135. | Heiner, J.D. and T.J. McArthur, The ultrasound identification of simulated long bone fractures by prehospital providers. Wilderness Environ Med, 2010. 21(2): p. 137-40. | Not in austere environment |
| 136. | Helwig, S.A., et al., Prehospital Stroke Management Optimized by Use of Clinical Scoring vs Mobile Stroke Unit for Triage of Patients With Stroke: A Randomized Clinical Trial. JAMA Neurol, 2019. 76(12): p. 1484-1492. | Not POCUS |
| 137. | Hernandez Torres, S.I., et al., Deep learning models for interpretation of point of care ultrasound in military working dogs. Front Vet Sci, 2024. 11: p. 1374890. | Not in humans |
| 138. | Hernandez Torres, S.I., et al., Evaluation of Deep Learning Model Architectures for Point-of-Care Ultrasound Diagnostics. Bioengineering (Basel), 2024. 11(4). | Not in austere environment |
| 139. | Hernández-Neuta, I., et al., Smartphone-based clinical diagnostics: towards democratization of evidence-based health care. J Intern Med, 2019. 285(1): p. 19-39. | Not POCUS |
| 140. | Herrera, P.M., et al., Implementation of an International Severe Infection Point-of-Care Ultrasound Research Network. Mil Med, 2024. 189(5-6): p. e1246-e1252. | Not in austere environment |
| 141. | Herzberg, M., et al., Prehospital stroke diagnostics based on neurological examination and transcranial ultrasound. Crit Ultrasound J, 2014. 6(1): p. 3. | Not in austere environment |
| 142. | Hinrichs-Krapels, S., et al., Barriers and facilitators for the provision of radiology services in Zimbabwe: A qualitative study based on staff experiences and observations. PLOS Glob Public Health, 2023. 3(4): p. e0001796. | Not POCUS |
| 143. | Hoffmann, B., et al., Bedside ultrasound of the neck confirms endotracheal tube position in emergency intubations. Ultraschall Med, 2014. 35(5): p. 451-8. | Not in austere environment |
| 144. | Holmström, O., et al., Point-of-Care Digital Cytology With Artificial Intelligence for Cervical Cancer Screening in a Resource-Limited Setting. JAMA Netw Open, 2021. 4(3): p. e211740. | Not POCUS |
| 145. | Holmström, O., et al., Detection of breast cancer lymph node metastases in frozen sections with a point-of-care low-cost microscope scanner. PLoS One, 2019. 14(3): p. e0208366. | Not POCUS |
| 146. | Hölscher, T., et al., Prehospital stroke diagnosis and treatment in ambulances and helicopters-a concept paper. Am J Emerg Med, 2013. 31(4): p. 743-7. | Not POCUS |
| 147. | Hoppmann, R.A., et al., International consensus conference recommendations on ultrasound education for undergraduate medical students. Ultrasound J, 2022. 14(1): p. 31. | Curriculum development |
| 148. | Hov, M.R., et al., Interpretation of Brain CT Scans in the Field by Critical Care Physicians in a Mobile Stroke Unit. J Neuroimaging, 2018. 28(1): p. 106-111. | Not POCUS |
| 149. | Hu, J., et al., Portable microfluidic and smartphone-based devices for monitoring of cardiovascular diseases at the point of care. Biotechnol Adv, 2016. 34(3): p. 305-20. | Not POCUS |
| 150. | Hu, J., et al., Dynamic Observation on Opening of the Blood-Brain Barrier in the Primary Stage of Severely Scalded Rabbits, a Multimodal Study. J Burn Care Res, 2016. 37(3): p. e279-86. | Not in humans |
| 151. | Huang, H., et al., Dynamic monitoring of cardiac foreign body by perioperative echocardiography: A case study. Echocardiography, 2021. 38(4): p. 676-680. | Not in austere environment |
| 152. | Huang, W., et al., Applications of smartphone-based near-infrared (NIR) imaging, measurement, and spectroscopy technologies to point-of-care (POC) diagnostics. J Zhejiang Univ Sci B, 2021. 22(3): p. 171-189. | Not POCUS |
| 153. | Hughey, S., et al., Point-of-Care Ultrasound in Austere Environments. Wilderness Environ Med, 2023. 34(2): p. 260-262. | No abstract |
| 154. | Hussain, A., et al., Multi-organ point-of-care ultrasound for COVID-19 (PoCUS4COVID): international expert consensus. Crit Care, 2020. 24(1): p. 702. | Not in austere environment |
| 155. | Hussein, L., et al., Transoesophageal echocardiography in cardiac arrest: A systematic review. Resuscitation, 2021. 168: p. 167-175. | Not in austere environment |
| 156. | Hussein, L., et al., Bedside ultrasound in cardiac standstill: a clinical review. Ultrasound J, 2019. 11(1): p. 35. | Not in austere environment |
| 157. | Hwang, S.H., et al., Rapid visual identification of PCR amplified nucleic acids by centrifugal gel separation: Potential use for molecular point-of-care tests. Biosens Bioelectron, 2016. 79: p. 829-34. | Not POCUS |
| 158. | Inan, H., et al., Isolation, Detection, and Quantification of Cancer Biomarkers in HPV-Associated Malignancies. Sci Rep, 2017. 7(1): p. 3322. | Not POCUS |
| 159. | Inscoe, C.R., et al., Point-of-Care Tomosynthesis Imaging of the Wrist. Mil Med, 2021. 186(Suppl 1): p. 745-750. | Not POCUS |
| 160. | Issadore, D., et al., Miniature magnetic resonance system for point-of-care diagnostics. Lab Chip, 2011. 11(13): p. 2282-7. | Not POCUS |
| 161. | Iyengar, N.M., et al., Impact of COVID-19 on patients with metastatic breast cancer: REthink Access to Care and Treatment survey results. Future Oncol, 2024: p. 1-13. | Not POCUS |
| 162. | Jacobsen, L., et al., Feasibility of prehospital identification of non-ST-elevation myocardial infarction by ECG, troponin and echocardiography. Emerg Med J, 2022. 39(9): p. 679-684. | Not in austere environment |
| 163. | Jacobson, N., et al., Mortality in the Emergency Department and the Effectiveness of Conventional Safety Event Reporting. Cureus, 2023. 15(9): p. e45472. | Not POCUS |
| 164. | Jeffers, K.L., et al., Point of Care Ultrasounds Obtained by Novice Physician Assistant Residents (POCUS ON PAR). Mil Med, 2024. | Not in physician healthcare workers |
| 165. | Jiang, C., et al., Changes in the cellular immune system and circulating inflammatory markers of stroke patients. Oncotarget, 2017. 8(2): p. 3553-3567. | Not POCUS |
| 166. | Jirawison, C., et al., Telemedicine screening for cytomegalovirus retinitis at the point of care for human immunodeficiency virus infection. JAMA Ophthalmol, 2015. 133(2): p. 198-205. | Not POCUS |
| 167. | Joh, D.Y., et al., Cellphone enabled point-of-care assessment of breast tumor cytology and molecular HER2 expression from fine-needle aspirates. NPJ Breast Cancer, 2021. 7(1): p. 85. | Not POCUS |
| 168. | John, S., et al., Brain Imaging Using Mobile CT: Current Status and Future Prospects. J Neuroimaging, 2016. 26(1): p. 5-15. | Not POCUS |
| 169. | Johnson, B., et al., Team-focused Cardiopulmonary Resuscitation: Prehospital Principles Adapted for Emergency Department Cardiac Arrest Resuscitation. J Emerg Med, 2018. 54(1): p. 54-63. | Not in austere environment |
| 170. | Jonas, E., et al., Treatment of hepatocellular carcinoma in sub-Saharan Africa: challenges and solutions. Lancet Gastroenterol Hepatol, 2022. 7(11): p. 1049-1060. | Not POCUS |
| 171. | Joyce, L., et al., Young female with abdominal pain and intra-abdominal free fluid: The risk of confirmation bias associated with point-of-care ultrasound. Australas J Ultrasound Med, 2022. 25(4): p. 207-209. | Not in austere environment |
| 172. | Junge, K., et al., Education in Focused Assessment With Sonography for Trauma Using Immersive Virtual Reality: A Prospective, Interventional Cohort Study and Non-inferiority Analysis With a Historical Control. Ultrasound Med Biol, 2024. 50(2): p. 277-284. | Not in austere environment |
| 173. | Kaffes, M., et al., Optimization of sensitivity and specificity of a biomarker-based blood test (LVOCheck-Opti): A protocol for a multicenter prospective observational study of patients suspected of having a stroke. Front Neurol, 2023. 14: p. 1327348. | Not POCUS |
| 174. | Kagima, J., et al., Diagnostic accuracy of combined thoracic and cardiac sonography for the diagnosis of pulmonary embolism: A systematic review and meta-analysis. PLoS One, 2020. 15(9): p. e0235940. | Not in austere environment |
| 175. | Kalkwarf, K.J., et al., Prehospital ABC Score Accurately Forecasts Patients Who Will Require Immediate Resource Utilization. South Med J, 2021. 114(4): p. 193-198. | Not POCUS |
| 176. | Kang, B.H., et al., Ultrafast Plasmonic Nucleic Acid Amplification and Real-Time Quantification for Decentralized Molecular Diagnostics. ACS Nano, 2023. 17(7): p. 6507-6518. | Not POCUS |
| 177. | Kanneganti, V., et al., Clinical and Laboratory Markers of Brain Abscess in Tetralogy of Fallot ('BA-TOF' Score): Results of a Case-Control Study and Implications for Community Surveillance. J Neurosci Rural Pract, 2021. 12(2): p. 302-307. | Not POCUS |
| 178. | Karfunkle, B., et al., COVID-19 Acute Respiratory Distress Syndrome and Pulmonary Embolism: A Case Report of Nebulized Nitroglycerin and Systemic Thrombolysis For Right Ventricular Failure. J Emerg Med, 2021. 61(5): p. e103-e107. | Not in austere environment |
| 179. | Karsteter, P.A. and C. Yunker, Recognition and management of an orbital blowout fracture in an amateur boxer. J Orthop Sports Phys Ther, 2006. 36(8): p. 611-8. | Not POCUS |
| 180. | Kassahun, E.A., et al., Development and validation of a simplified risk prediction model for preterm birth: a prospective cohort study in rural Ethiopia. Sci Rep, 2024. 14(1): p. 4845. | Not POCUS |
| 181. | Kassutto, S.M., C. Baston, and C. Clancy, Virtual, Augmented, and Alternate Reality in Medical Education: Socially Distanced but Fully Immersed. ATS Sch, 2021. 2(4): p. 651-664. | Not POCUS |
| 182. | Kaur, J., R. Srivastava, and V. Borse, Recent advances in point-of-care diagnostics for oral cancer. Biosens Bioelectron, 2021. 178: p. 112995. | Not POCUS |
| 183. | Kaur, N., J.S. Michael, and B.J. Toley, A modular paper-and-plastic device for tuberculosis nucleic acid amplification testing in limited-resource settings. Sci Rep, 2019. 9(1): p. 15367. | Not POCUS |
| 184. | Kemp, J.F., et al., Zinc Supplementation Initiated Prior to or During Pregnancy Modestly Impacted Maternal Status and High Prevalence of Hypozincemia in Pregnancy and Lactation: The Women First Preconception Maternal Nutrition Trial. J Nutr, 2024. 154(6): p. 1917-1926. | Not POCUS |
| 185. | Kettner, M., et al., Prehospital Computed Tomography Angiography in Acute Stroke Management. Cerebrovasc Dis, 2017. 44(5-6): p. 338-343. | Not POCUS |
| 186. | Keuski, B.M., Updates in diving medicine: evidence published in 2017-2018. Undersea Hyperb Med, 2018. 45: p. 511-520. | Not POCUS |
| 187. | Kilgore, A.E., et al., Can Emergency Physicians Diagnose Cirrhosis by Ultrasound: A Prospective Single-Arm Educational Intervention. Cureus, 2023. 15(4): p. e38012. | Not in austere environment |
| 188. | Kilic, M., et al., A mobile battery-powered brain perfusion ultrasound (BPU) device designed for prehospital stroke diagnosis: correlation to perfusion MRI in healthy volunteers. Neurol Res Pract, 2022. 4(1): p. 13. | Not POCUS |
| 189. | Kilic, M., et al., Acute Middle Cerebral Artery Occlusion Detection Using Mobile Non-Imaging Brain Perfusion Ultrasound-First Case. J Clin Med, 2022. 11(12). | Not in austere environment |
| 190. | Kim, Y., et al., A Portable Smartphone-Based Laryngoscope System for High-Speed Vocal Cord Imaging of Patients With Throat Disorders: Instrument Validation Study. JMIR Mhealth Uhealth, 2021. 9(6): p. e25816. | Not POCUS |
| 191. | Kirkpatrick, A.W., Point-of-care resuscitation research: From extreme to mainstream: Trauma Association of Canada Fraser Gurd Lecture 2019. J Trauma Acute Care Surg, 2019. 87(3): p. 571-581. | Not in austere environment |
| 192. | Kirkpatrick, A.W., et al., Hand-held portable sonography for the on-mountain exclusion of a pneumothorax. Wilderness Environ Med, 2001. 12(4): p. 270-2. | Not involving healthcare workers |
| 193. | Kirkpatrick, A.W., et al., Patient Self-Performed Point-of-Care Ultrasound: Using Communication Technologies to Empower Patient Self-Care. Diagnostics (Basel), 2022. 12(11). | Not in austere environment |
| 194. | Kissoon, D.V., et al., Observational descriptive study of ultrasound use and its impact on clinical decisions in the accident and emergency department at Georgetown public hospital corporation. PLoS One, 2020. 15(5): p. e0233379. | Not in austere environment |
| 195. | Kocarnik, J.M., et al., Cancer Incidence, Mortality, Years of Life Lost, Years Lived With Disability, and Disability-Adjusted Life Years for 29 Cancer Groups From 2010 to 2019: A Systematic Analysis for the Global Burden of Disease Study 2019. JAMA Oncol, 2022. 8(3): p. 420-444. | Not POCUS |
| 196. | Kostopoulos, P., et al., Mobile stroke unit for diagnosis-based triage of persons with suspected stroke. Neurology, 2012. 78(23): p. 1849-52. | Not POCUS |
| 197. | Kotagal, M., et al., Impact of point-of-care ultrasound training on surgical residents' confidence. J Surg Educ, 2015. 72(4): p. e82-7. | Not in austere environment |
| 198. | Kwizera, R., et al., Misdiagnosis of chronic pulmonary aspergillosis as pulmonary tuberculosis at a tertiary care center in Uganda: a case series. J Med Case Rep, 2021. 15(1): p. 140. | Not POCUS |
| 199. | Kwon, A.S., S. Lahham, and J.C. Fox, Can an 8(th) grade student learn point of care ultrasound? World J Emerg Med, 2019. 10(2): p. 109-113. | Curriculum development |
| 200. | Lai, J., et al., Prehospital Ultrasound Use to Guide Resuscitative Thoracotomy in Blunt Traumatic Cardiac Arrest. Air Med J, 2022. 41(5): p. 494-497. | Not in physician healthcare workers |
| 201. | Lam, S.H.F., et al., The P2Network-Advancing Pediatric Emergency Care With Point-of-Care Ultrasound. Pediatr Emerg Care, 2022. 38(2): p. e1014-e1018. | Curriculum development |
| 202. | Lam, V. and C.H. Hsu, Updates in Cardiac Arrest Resuscitation. Emerg Med Clin North Am, 2020. 38(4): p. 755-769. | Not in austere environment |
| 203. | Lane, A.B., et al., Clinical Importance of Echogenic Swirling Pleural Effusions. J Ultrasound Med, 2016. 35(4): p. 843-7. | Not in austere environment |
| 204. | Langlois Sle, P., Focused ultrasound training for clinicians. Crit Care Med, 2007. 35(5 Suppl): p. S138-43. | Curriculum development |
| 205. | Le, M.T., et al., Comparison of four handheld point-of-care ultrasound devices by expert users. Ultrasound J, 2022. 14(1): p. 27. | Not in austere environment |
| 206. | Le, N.N. and M. Riscinti, Using Real-Time Deep Learning Algorithms to Assist Novice Learners Acquire Cardiothoracic Ultrasound Images. Academic Emergency Medicine, 2023. 30: p. 394. | Not in austere environment |
| 207. | Leggett, C.B., et al., Incorporating personal-device-based point-of-care ultrasound into obstetric care: a validation study. Am J Obstet Gynecol, 2022. 226(4): p. 552.e1-552.e6. | Not in austere environment |
| 208. | Lenz, A., et al., The next nine minutes: Lessons learned from the large-scale active shooter training prior to the STEM school shooting. Am J Disaster Med, 2020. 15(4): p. 241-249. | Not POCUS |
| 209. | Levitt, C.V., et al., Application of Technology in Cardiopulmonary Resuscitation, a Narrative Review. J Clin Med, 2023. 12(23). | Not POCUS |
| 210. | Levy, P.D., T. Wielinski, and A. Greszler, Micropower impulse radar: a novel technology for rapid, real-time detection of pneumothorax. Emerg Med Int, 2011. 2011: p. 279508. | Not POCUS |
| 211. | Li, C., et al., The correlation between lipoprotein(a) and coronary atherosclerotic lesion is stronger than LDL-C, when LDL-C is less than 104 mg/dL. BMC Cardiovasc Disord, 2021. 21(1): p. 41. | Not POCUS |
| 212. | Li, R., et al., The predictive value of four traumatic hemorrhage scores for early massive blood transfusion in trauma patients in the pre-hospital setting. Eur J Trauma Emerg Surg, 2024. 50(3): p. 967-973. | Not POCUS |
| 213. | Li, Y., et al., A low-cost, automated parasite diagnostic system via a portable, robotic microscope and deep learning. J Biophotonics, 2019. 12(9): p. e201800410. | Not POCUS |
| 214. | Lifson, M.A., et al., Advances in biosensing strategies for HIV-1 detection, diagnosis, and therapeutic monitoring. Adv Drug Deliv Rev, 2016. 103: p. 90-104. | Not POCUS |
| 215. | Lightfoot, A. and J. Chan, Unexpected severe intra-abdominal injuries resulting from a ground-level fall in an elderly patient with a large staghorn calculus. BMJ Case Rep, 2021. 14(3). | Not in austere environment |
| 216. | Lim, T.R., et al., Low-Field (64mT) Portable MRI for Rapid Point-of-Care Diagnosis of DIS in Patients Presenting with Optic Neuritis. AJNR Am J Neuroradiol, 2024. | Not POCUS |
| 217. | Liotta, E.M., et al., Magnesium and Hematoma Expansion in Intracerebral Hemorrhage: A FAST-MAG Randomized Trial Analysis. Stroke, 2024. 55(2): p. 463-466. | Not POCUS |
| 218. | Lipman, G.S., J.T. Marvel, and P. Burns, Re: "Ultrasound in Austere Environments" by Canepa and Harris (High Alt Med Biol 2019;20:103-111). High Alt Med Biol, 2019. 20(4): p. 440. | No abstract |
| 219. | Liu, J., et al., Diagnosis of neonatal transient tachypnea and its differentiation from respiratory distress syndrome using lung ultrasound. Medicine (Baltimore), 2014. 93(27): p. e197. | Not in austere environment |
| 220. | Liu, J., et al., Extended Stent Coverage Decreases Distal Aortic Segmental Enlargement After the Endovascular Repair of Acute Complicated Type B Aortic Dissection: A Multi-Center Retrospective Study of 814 Patients. J Endovasc Ther, 2022. 29(1): p. 96-108. | Not POCUS |
| 221. | Liu, L., et al., Interventional therapy in sarcoidosis-associated pulmonary arterial stenosis and pulmonary hypertension. Clin Respir J, 2017. 11(6): p. 906-914. | Not POCUS |
| 222. | Liu, P.Y., et al., Prevalence and characteristics of mitral valve prolapse in military young adults in Taiwan of the CHIEF Heart Study. Sci Rep, 2021. 11(1): p. 2719. | Not POCUS |
| 223. | Liu, X., et al., Multiplex digital microfluidics using serial controls and its applications in glucose sensing. SLAS Technol, 2024. 29(2): p. 100105. | Not POCUS |
| 224. | Liu, Y., et al., A low-cost and shielding-free ultra-low-field brain MRI scanner. Nat Commun, 2021. 12(1): p. 7238. | Not POCUS |
| 225. | Lochhead, M.J., et al., Rapid multiplexed immunoassay for simultaneous serodiagnosis of HIV-1 and coinfections. J Clin Microbiol, 2011. 49(10): p. 3584-90. | Not POCUS |
| 226. | Long, B., et al., Echocardiography in cardiac arrest: An emergency medicine review. Am J Emerg Med, 2018. 36(3): p. 488-493. | Not in austere environment |
| 227. | Lorenz, M.W., A. Lauer, and C. Foerch, Quantifying the Benefit of Prehospital Rapid Treatment in Acute Stroke: Benchmark for Future Innovative Clinical Trials. Stroke, 2015. 46(11): p. 3168-76. | Not POCUS |
| 228. | Lu, F.L., et al., Transcatheter tricuspid valve replacement in patients with severe tricuspid regurgitation. Heart, 2021. 107(20): p. 1664-1670. | Not POCUS |
| 229. | Lu, J.C., et al., Simplified rheumatic heart disease screening criteria for handheld echocardiography. J Am Soc Echocardiogr, 2015. 28(4): p. 463-9. | Not POCUS |
| 230. | Luebke, M.C., et al., Developing a urinary incontinence primary care pathway: a mixed methods study. Fam Pract, 2024. | Not POCUS |
| 231. | Luger, S., et al., Diagnostic Accuracy of Glial Fibrillary Acidic Protein and Ubiquitin Carboxy-Terminal Hydrolase-L1 Serum Concentrations for Differentiating Acute Intracerebral Hemorrhage from Ischemic Stroke. Neurocrit Care, 2020. 33(1): p. 39-48. | Not POCUS |
| 232. | Luttrell, K., M. Beltran, and C.A. Collinge, Preoperative decision making in the treatment of high-angle "vertical" femoral neck fractures in young adult patients. An expert opinion survey of the Orthopaedic Trauma Association's (OTA) membership. J Orthop Trauma, 2014. 28(9): p. e221-5. | Not POCUS |
| 233. | MacDonald, R.D. and S. Alqattan, Articles That May Change Your Practice: Prehospital Ultrasound. Air Med J, 2017. 36(1): p. 16-18. | No abstract |
| 234. | Maddry, J.K., et al., En Route Resuscitation - Utilization of CCATT to Transport and Stabilize Critically Injured and Unstable Casualties. Mil Med, 2019. 184(5-6): p. e172-e176. | Not POCUS |
| 235. | Magon, F., et al., Point-of-Care Ultrasound (POCUS) in Adult Cardiac Arrest: Clinical Review. Diagnostics (Basel), 2024. 14(4). | Not in austere environment |
| 236. | Malicek, D., et al., Proteomics-Based Approach to Identify Novel Blood Biomarker Candidates for Differentiating Intracerebral Hemorrhage From Ischemic Stroke-A Pilot Study. Front Neurol, 2021. 12: p. 713124. | Not POCUS |
| 237. | Mancusi, C., M.V. Carlino, and A. Sforza, Point-of-care ultrasound with pocket-size devices in emergency department. Echocardiography, 2019. 36(9): p. 1755-1764. |  |
| 238. | Mani, V., et al., Emerging technologies for monitoring drug-resistant tuberculosis at the point-of-care. Adv Drug Deliv Rev, 2014. 78: p. 105-17. | Not POCUS |
| 239. | Manley, J.D., et al., A Modern Case Series of Resuscitative Endovascular Balloon Occlusion of the Aorta (REBOA) in an Out-of-Hospital, Combat Casualty Care Setting. J Spec Oper Med, 2017. 17(1): p. 1-8. | Not POCUS |
| 240. | Marshburn, T.H., et al., Goal-directed ultrasound in the detection of long-bone fractures. J Trauma, 2004. 57(2): p. 329-32. | Not in austere environment |
| 241. | Martin, R., et al., The Rising Tide of Point-of-Care Ultrasound (POCUS) in Medical Education: An Essential Skillset for Undergraduate and Graduate Medical Education. Curr Probl Diagn Radiol, 2023. 52(6): p. 482-484. | Curriculum development |
| 242. | Mati, B. and M.A. Silver, Diagnosing and Treating an Acute Anterior Shoulder Dislocation Using Point-of-Care Ultrasound in an Urgent Care Setting. Perm J, 2022. 26(3): p. 135-138. | Not in austere environment |
| 243. | Mauermann, E., et al., Rapid, Single-View Speckle-Tracking-Based Method for Examining Left Ventricular Systolic and Diastolic Function in Point of Care Ultrasound. J Ultrasound Med, 2020. 39(11): p. 2151-2164. | Not in austere environment |
| 244. | Maximous, S., et al., Pragmatic Recommendations for the Management of COVID-19 Patients with Shock in Low- and Middle-Income Countries. Am J Trop Med Hyg, 2020. 104(3_Suppl): p. 72-86. | Not POCUS |
| 245. | McCrea, M.A., et al., Opportunities for Prevention of Concussion and Repetitive Head Impact Exposure in College Football Players: A Concussion Assessment, Research, and Education (CARE) Consortium Study. JAMA Neurol, 2021. 78(3): p. 346-350. | Not POCUS |
| 246. | McFall, S.M., et al., C-THAN: A new research center for the development of point-of-care technology for HIV/AIDS. Glob Health Innov, 2019. 2(2): p. 1-5. | Not POCUS |
| 247. | McManus, J.G., et al., Use of ultrasound to assess acute fracture reduction in emergency care settings. Am J Disaster Med, 2008. 3(4): p. 241-7. | Not in austere environment |
| 248. | Mellor, T.E., et al., Not Just Hocus POCUS: Implementation of a Point of Care Ultrasound Curriculum for Internal Medicine Trainees at a Large Residency Program. Mil Med, 2019. 184(11-12): p. 901-906. | Curriculum development |
| 249. | Merrill, D. and M. Myers, A Case Report Utilizing Ultrasound for the Identification of Traumatic Pulmonary Contusion. Med J (Ft Sam Houst Tex), 2021(Pb 8-21-04/05/06): p. 98-99. | Not in austere environment |
| 250. | Miarka, M., et al., Liver volume: a point of no return in liver transplantation? Pol Arch Intern Med, 2020. 130(7-8): p. 622-628. | Not in austere environment |
| 251. | Mielnicki, W., et al., Utility of tissue Doppler imaging of systolic function to diagnose diastolic dysfunction in critically ill patients. Anaesthesiol Intensive Ther, 2019. 51(4): p. 268-272. | Not POCUS |
| 252. | Milton, S.C., A.J. Cronin, and J.D. Monti, Evaluation of Glucomannan Powder as an Ultrasound Transmission Gel Alternative for Resource-Constrained Environments: A Prospective, Comparative Study. Wilderness Environ Med, 2018. 29(4): p. 446-452. | Not POCUS |
| 253. | Mitra, M., et al., Multiwavelength laser diode based portable photoacoustic and ultrasound imaging system for point of care applications. J Biophotonics, 2024. 17(7): p. e202400058. | Not POCUS |
| 254. | Monti, J.D., A Novel Ultrasound Transmission Gel for Resource-Constrained Environments. J Spec Oper Med, 2017. 17(1): p. 22-25. | No abstract |
| 255. | Montorfano, M.A., et al., The FAST D protocol: a simple method to rule out traumatic vascular injuries of the lower extremities. Crit Ultrasound J, 2017. 9(1): p. 8. | Not in austere environment |
| 256. | Montoya, J., et al., From FAST to E-FAST: an overview of the evolution of ultrasound-based traumatic injury assessment. Eur J Trauma Emerg Surg, 2016. 42(2): p. 119-26. | No abstract |
| 257. | Mor, Z., et al., Chest radiography validity in screening pulmonary tuberculosis in immigrants from a high-burden country. Respir Care, 2012. 57(7): p. 1137-44. | Not POCUS |
| 258. | Mori, T., et al., Pediatric Case of Successful Point-of-Care Ultrasound-Guided Nasogastric Tube Placement. J Emerg Med, 2020. 59(2): p. e57-e60. | Not in austere environment |
| 259. | Mostafa, M.G., et al., Unguided percutaneous transthoracic fine needle aspiration cytology in the diagnosis of the peripheral lung lesions. Bangladesh Med Res Counc Bull, 1999. 25(1): p. 1-5. | Not POCUS |
| 260. | Mount, C., et al., Intravenous Fluid Bag as a Substitute for Gel Standoff Pad in Musculoskeletal Point-of-care Ultrasound. Mil Med, 2023. 188(5-6): p. e949-e952. | Not involving healthcare workers |
| 261. | Müller, V., et al., Identification of pathogenic bacteria in complex samples using a smartphone based fluorescence microscope. RSC Adv, 2018. 8(64): p. 36493-36502. | Not POCUS |
| 262. | Murthy, S.B., et al., A Pooled Analysis of Diffusion-Weighted Imaging Lesions in Patients With Acute Intracerebral Hemorrhage. JAMA Neurol, 2020. 77(11): p. 1390-1397. | Not POCUS |
| 263. | Mwanza, Z.V., et al., Proteinuric kidney disease in children at Queen Elizabeth Central Hospital, Malawi. BMC Nephrol, 2018. 19(1): p. 21. | Not POCUS |
| 264. | Nakwan, N. and P. Chaiwiriyawong, An international survey on persistent pulmonary hypertension of the newborn: A need for an evidence-based management. J Neonatal Perinatal Med, 2016. 9(3): p. 243-50. | Not POCUS |
| 265. | Nathanson, R., et al., Development of a Point-of-Care Ultrasound Track for Internal Medicine Residents. J Gen Intern Med, 2022. 37(9): p. 2308-2313. | Curriculum development |
| 266. | Naylor, J.F., et al., Non-battle Emergency Department Utilization of the First Modular Army Field Hospital Prototype in Support of Operation INHERENT RESOLVE. Mil Med, 2019. 184(5-6): p. e168-e171. | Not POCUS |
| 267. | Nelson, B.P. and A. Sanghvi, Point-of-Care Cardiac Ultrasound: Feasibility of Performance by Noncardiologists. Glob Heart, 2013. 8(4): p. 293-7. | Not in austere environment |
| 268. | Nolte, C.H. and H.J. Audebert, [Prehospital care for stroke patients]. Med Klin Intensivmed Notfmed, 2017. 112(8): p. 668-673. | Not POCUS |
| 269. | Noor, M.O. and U.J. Krull, Camera-based ratiometric fluorescence transduction of nucleic acid hybridization with reagentless signal amplification on a paper-based platform using immobilized quantum dots as donors. Anal Chem, 2014. 86(20): p. 10331-9. | Not POCUS |
| 270. | Nourse, P., et al., ISPD guidelines for peritoneal dialysis in acute kidney injury: 2020 Update (paediatrics). Perit Dial Int, 2021. 41(2): p. 139-157. | Not POCUS |
| 271. | O'Connor, F.G., et al., A pilot study of clinical agreement in cardiovascular preparticipation examinations: how good is the standard of care? Clin J Sport Med, 2005. 15(3): p. 177-9. | Not POCUS |
| 272. | Ochodo, E.A., et al., Point-of-care tests detecting HIV nucleic acids for diagnosis of HIV-1 or HIV-2 infection in infants and children aged 18 months or less. Cochrane Database Syst Rev, 2021. 8(8): p. Cd013207. | Not POCUS |
| 273. | Olatunji, R.B., et al., ROLE OF TRANSCRANIAL COLOUR-CODED DUPLEX SONOGRAPHY IN STROKE MANAGEMENT - REVIEW ARTICLE. West Afr J Ultrasound, 2015. 16(1): p. 33-42. | Not POCUS |
| 274. | Omori, K., et al., The Utility of a Portable X-ray System. Air Med J, 2019. 38(3): p. 212-214. | Not POCUS |
| 275. | Ong, J., et al., An international perspective of out-of-hospital cardiac arrest and cardiopulmonary resuscitation during the COVID-19 pandemic. Am J Emerg Med, 2021. 47: p. 192-197. | Not POCUS |
| 276. | Oostema, J.A., et al., Emergency Medical Services Compliance With Prehospital Stroke Quality Metrics Is Associated With Faster Stroke Evaluation and Treatment. Stroke, 2024. 55(1): p. 101-109. | Not POCUS |
| 277. | Owens, B.D., et al., Pathoanatomy of first-time, traumatic, anterior glenohumeral subluxation events. J Bone Joint Surg Am, 2010. 92(7): p. 1605-11. | Not POCUS |
| 278. | Pak, L.M., et al., Non-clinical Drivers of Variation in Preoperative MRI Utilization for Breast Cancer. Ann Surg Oncol, 2020. 27(9): p. 3414-3423. | Not POCUS |
| 279. | Palanca Arias, D., et al., Point-of-care ultrasound after attempted suicidal hanging. Arch Pediatr, 2021. 28(4): p. 342-344. | Not in austere environment |
| 280. | Palma, J. and E. Schott, Acute, simultaneous, bilateral rhegmatogenous retinal detachment diagnosed with bedside emergency ultrasound. Am J Emerg Med, 2013. 31(2): p. 466.e3-5. | Not in austere environment |
| 281. | Palma, J.K., Successful strategies for integrating bedside ultrasound into undergraduate medical education. Mil Med, 2015. 180(4 Suppl): p. 153-7. | Curriculum development |
| 282. | Palmer, B.L., J.D. Heiner, and E.J. Chin, Images in emergency medicine. Young man with fever and shortness of breath. Pneumonia confirmed by bedside ultrasonography. Ann Emerg Med, 2013. 61(4): p. 499, 505. | Not in austere environment |
| 283. | Papa, L., Potential Blood-based Biomarkers for Concussion. Sports Med Arthrosc Rev, 2016. 24(3): p. 108-15. | Not POCUS |
| 284. | Park, C.K.S., et al., Cost-effective, portable, patient-dedicated three-dimensional automated breast ultrasound for point-of-care breast cancer screening. Sci Rep, 2023. 13(1): p. 14390. | Not POCUS |
| 285. | Parra, S., et al., Development of Low-Cost Point-of-Care Technologies for Cervical Cancer Prevention Based on a Single-Board Computer. IEEE J Transl Eng Health Med, 2020. 8: p. 4300210. | Not POCUS |
| 286. | Parrish, S.C., et al., A 76-Year-Old Man With a 75 Pack-Year History of Smoking and a Pulmonary Nodule. Chest, 2017. 151(5): p. e99-e102. | Not POCUS |
| 287. | Patel, A., et al., Value of chest radiography in predicting treatment response in children aged 3-59 months with severe pneumonia. Int J Tuberc Lung Dis, 2008. 12(11): p. 1320-6. | Not POCUS |
| 288. | Pathania, D., et al., Holographic Assessment of Lymphoma Tissue (HALT) for Global Oncology Field Applications. Theranostics, 2016. 6(10): p. 1603-10. | Not POCUS |
| 289. | Pearlman, P.C., et al., The National Institutes of Health Affordable Cancer Technologies Program: Improving Access to Resource-Appropriate Technologies for Cancer Detection, Diagnosis, Monitoring, and Treatment in Low- and Middle-Income Countries. IEEE J Transl Eng Health Med, 2016. 4: p. 2800708. | Not POCUS |
| 290. | Pearlman, S.I., et al., Controlling Droplet Marangoni Flows to Improve Microscopy-Based TB Diagnosis. Diagnostics (Basel), 2021. 11(11). | Not POCUS |
| 291. | Perel, P., et al., Predicting early death in patients with traumatic bleeding: development and validation of prognostic model. Bmj, 2012. 345: p. e5166. | Not POCUS |
| 292. | Perry, L.A., et al., Glial fibrillary acidic protein for the early diagnosis of intracerebral hemorrhage: Systematic review and meta-analysis of diagnostic test accuracy. Int J Stroke, 2019. 14(4): p. 390-399. | Not POCUS |
| 293. | Pezy, P., et al., Fixed-Distance Model for Balloon Placement During Fluoroscopy-Free Resuscitative Endovascular Balloon Occlusion of the Aorta in a Civilian Population. JAMA Surg, 2017. 152(4): p. 351-358. | Not POCUS |
| 294. | Pierce, M.C., et al., Optical systems for point-of-care diagnostic instrumentation: analysis of imaging performance and cost. Ann Biomed Eng, 2014. 42(1): p. 231-40. | Not POCUS |
| 295. | Pikman Gavriely, R., et al., Manual Pressure Points Technique for Massive Hemorrhage Control-A Prospective Human Volunteer Study. Prehosp Emerg Care, 2023. 27(5): p. 586-591. | Not POCUS |
| 296. | Pinto, J., et al., Ultrasonography in Gastroenterology: The Need for Training. GE Port J Gastroenterol, 2018. 25(6): p. 308-316. | Not in austere environment |
| 297. | Pittayapat, P., et al., Image quality assessment and medical physics evaluation of different portable dental X-ray units. Forensic Sci Int, 2010. 201(1-3): p. 112-7. | Not POCUS |
| 298. | Polcz, J.E., et al., Temporary intravascular shunt use improves early limb salvage after extremity vascular injury. J Vasc Surg, 2021. 73(4): p. 1304-1313. | Not POCUS |
| 299. | Polusny, M.A., et al., Adaptation in Young Military Recruits: Protocol for the Advancing Research on Mechanisms of Resilience (ARMOR) Prospective Longitudinal Study. JMIR Res Protoc, 2023. 12: p. e51235. | Not POCUS |
| 300. | Polusny, M.A., et al., Advancing Research on Mechanisms of Resilience (ARMOR) Prospective Longitudinal Study of Adaptation in Young Military Recruits: Protocol and rationale for methods and measures. medRxiv, 2023. | Not POCUS |
| 301. | Pool, K.L., et al., A Structured Global Health Training Program for Radiology Residents. J Am Coll Radiol, 2018. 15(2): p. 334-339. | Not POCUS |
| 302. | Potter, C.J., et al., Point-of-care SARS-CoV-2 sensing using lens-free imaging and a deep learning-assisted quantitative agglutination assay. Lab Chip, 2022. 22(19): p. 3744-3754. | Not POCUS |
| 303. | Presley, B.C. and M.J. Flannigan, Emergency department bedside ultrasound diagnosis of retinoblastoma in a child. Pediatr Emerg Care, 2013. 29(10): p. 1128-31. | Not in austere environment |
| 304. | Priye, A. and V.M. Ugaz, Smartphone-Enabled Detection Strategies for Portable PCR-Based Diagnostics. Methods Mol Biol, 2017. 1571: p. 251-266. | Not POCUS |
| 305. | Prunet, B., et al., Noninvasive detection of elevated intracranial pressure using a portable ultrasound system. Am J Emerg Med, 2012. 30(6): p. 936-41. | Not POCUS |
| 306. | Quinn, M.K., et al., High-resolution microendoscopy for the detection of cervical neoplasia in low-resource settings. PLoS One, 2012. 7(9): p. e44924. | Not POCUS |
| 307. | Rahsepar, S., et al., Point-of-Care Tests' Role in Time Metrics of Urgent Interventions in Emergency Department; a Systematic Review of Literature. Arch Acad Emerg Med, 2022. 10(1): p. e82. | Not POCUS |
| 308. | Rai, E., R. Alaraimi, and I. Al Aamri, Pediatric lower respiratory tract infection: Considerations for the anesthesiologist. Paediatr Anaesth, 2022. 32(2): p. 181-190. | Not POCUS |
| 309. | Rajsic, S., et al., Diagnostic Modalities in Critical Care: Point-of-Care Approach. Diagnostics (Basel), 2021. 11(12). | Not POCUS |
| 310. | Ramachandraiah, H., et al., Lab-on-DVD: standard DVD drives as a novel laser scanning microscope for image based point of care diagnostics. Lab Chip, 2013. 13(8): p. 1578-85. | Not POCUS |
| 311. | Ramsingh, D., et al., Use of a Smartphone-Based Augmented Reality Video Conference App to Remotely Guide a Point of Care Ultrasound Examination. Diagnostics (Basel), 2019. 9(4). | Not in healthcare workers |
| 312. | Rasmussen, M.B., et al., Comparison of Acute Versus Subacute Coronary Angiography in Patients With NON-ST-Elevation Myocardial Infarction (from the NONSTEMI Trial). Am J Cardiol, 2019. 124(6): p. 825-832. | Not POCUS |
| 313. | Rasooli, F., et al., Comparison of Emergency Echocardiographic Results between Cardiologists and an Emergency Medicine Resident in Acute Coronary Syndrome. Arch Acad Emerg Med, 2021. 9(1): p. e53. | Not in austere environment |
| 314. | Rasooli, F., M.A. Zahraie, and M. Bahreini, Point-of-care ultrasound to complete physical exam and to reach the diagnosis in a young man with syncope. Ultrasound J, 2020. 12(1): p. 29. | Not in austere environment |
| 315. | Rasooly, R., et al., Improving the Sensitivity and Functionality of Mobile Webcam-Based Fluorescence Detectors for Point-of-Care Diagnostics in Global Health. Diagnostics (Basel), 2016. 6(2). | Not POCUS |
| 316. | Reischl, A.T., et al., The clinical impact of PCR-based point-of-care diagnostic in respiratory tract infections in children. J Clin Lab Anal, 2020. 34(5): p. e23203. | Not POCUS |
| 317. | Rey, E.G., J.L. Finkelstein, and D. Erickson, Point-of-Care Assessment of Folate Status in Women of Reproductive Age Using a Fluorescence Lateral Flow Assay(). Annu Int Conf IEEE Eng Med Biol Soc, 2018. 2018: p. 3906-3909. | Not POCUS |
| 318. | Rhon, D.I., G.D. Deyle, and N.W. Gill, Clinical reasoning and advanced practice privileges enable physical therapist point-of-care decisions in the military health care system: 3 clinical cases. Phys Ther, 2013. 93(9): p. 1234-43. | Not POCUS |
| 319. | Richards, E., S. Munakomi, and D. Mathew, Optic Nerve Sheath Ultrasound, in StatPearls. 2024, StatPearls Publishing | Not in austere environment |
| 320. | Roberts, D.R., et al., Mobile point-of-care MRI demonstration of a normal volunteer in a telemedicine-equipped ambulance. J Stroke Cerebrovasc Dis, 2023. 32(10): p. 107301. | Not POCUS |
| 321. | Robertson, T.E., et al., Remote tele-mentored ultrasound for non-physician learners using FaceTime: A feasibility study in a low-income country. J Crit Care, 2017. 40: p. 145-148. | Not physician healthcare workers |
| 322. | Rong, Z., et al., Dual-color magnetic-quantum dot nanobeads as versatile fluorescent probes in test strip for simultaneous point-of-care detection of free and complexed prostate-specific antigen. Biosens Bioelectron, 2019. 145: p. 111719. | Not POCUS |
| 323. | Rozanski, M., et al., Glial Fibrillary Acidic Protein for Prehospital Diagnosis of Intracerebral Hemorrhage. Cerebrovasc Dis, 2017. 43(1-2): p. 76-81. | Not POCUS |
| 324. | Rumpf, T.H., M. Krizmaric, and S. Grmec, Capnometry in suspected pulmonary embolism with positive D-dimer in the field. Crit Care, 2009. 13(6): p. R196. | Not POCUS |
| 325. | Rusina, R., et al., Superficial bedside brain biopsy can be a safe and practical approach to confirm a rare form of prion disease in cerebellar ataxia: A case study. J Neurol Sci, 2017. 375: p. 73-75. | Not POCUS |
| 326. | Sacchettini, A., et al., [Interdisciplinarity in prehospital care:collaboration for better care]. Rev Med Suisse, 2022. 18(791): p. 1504-1506. | Not POCUS |
| 327. | Sagreiya, H., M.A. Jacobs, and A. Akhbardeh, Automated Lung Ultrasound Pulmonary Disease Quantification Using an Unsupervised Machine Learning Technique for COVID-19. Diagnostics (Basel), 2023. 13(16). | Not in healthcare workers |
| 328. | Saltzherr, T.P., et al., An evaluation of a Shockroom located CT scanner: a randomized study of early assessment by CT scanning in trauma patients in the bi-located trauma center North-West Netherlands (REACT trial). BMC Emerg Med, 2008. 8: p. 10. | Not POCUS |
| 329. | Sanossian, N. and E. Fink, What Will the Mobile Stroke Unit of the Future Look Like, and Will EEG Have a Role? Neurology, 2023. 101(24): p. 1085-1086. | Not POCUS |
| 330. | Saravanan, P., et al., Early pregnancy HbA(1c) as the first screening test for gestational diabetes: results from three prospective cohorts. Lancet Diabetes Endocrinol, 2024. 12(8): p. 535-544. | Not POCUS |
| 331. | Sayed, S., et al., Breast Camps for Awareness and Early Diagnosis of Breast Cancer in Countries With Limited Resources: A Multidisciplinary Model From Kenya. Oncologist, 2016. 21(9): p. 1138-48. | Not POCUS |
| 332. | Sazal, H.R., et al., Clinico-Pathological Profile and Outcome of Multiple Ultrasonogram Guided Aspiration of Breast Abscess in Outpatient Department Setup: A Prospective Study. Mymensingh Med J, 2024. 33(2): p. 453-460. | Not in austere environment |
| 333. | Sazawal, S., et al., Machine learning guided postnatal gestational age assessment using new-born screening metabolomic data in South Asia and sub-Saharan Africa. BMC Pregnancy Childbirth, 2021. 21(1): p. 609. | Not POCUS |
| 334. | Schenck, E.J. and K. Rajwani, Ultrasound in the diagnosis and management of pneumonia. Curr Opin Infect Dis, 2016. 29(2): p. 223-8. | Not in austere environment |
| 335. | Schermann, H., et al., Use of a Lightweight Portable Fluoroscopy Device for Obtaining Weightbearing Ankle Images. J Foot Ankle Surg, 2023. 62(1): p. 102-106. | Not POCUS |
| 336. | Scheuzger, J.D., et al., Sublingual microcirculation: a case report. J Med Case Rep, 2019. 13(1): p. 179. | Not POCUS |
| 337. | Schmitz, G., B. Long, and M.D. April, Are Emergency Practitioners Able to Diagnose Posterior Chamber Abnormalities With Point-of-Care Ocular Ultrasonography? Ann Emerg Med, 2020. 76(6): p. 767-769. | Not in austere environment |
| 338. | Schmitz, G.R. and M. Gottlieb, Managing a Cutaneous Abscess in the Emergency Department. Ann Emerg Med, 2021. 78(1): p. 44-48. | Not in austere environment |
| 339. | Schultz, M.J., et al., Pragmatic Recommendations for the Use of Diagnostic Testing and Prognostic Models in Hospitalized Patients with Severe COVID-19 in Low- and Middle-Income Countries. Am J Trop Med Hyg, 2021. 104(3_Suppl): p. 34-47. | Not POCUS |
| 340. | Schwindling, L., et al., Prehospital Imaging-Based Triage of Head Trauma with a Mobile Stroke Unit: First Evidence and Literature Review. J Neuroimaging, 2016. 26(5): p. 489-93. | No abstract |
| 341. | Sebt, S., et al., Acute Thromboembolism from Trauma in a Patient with Abdominal Aortic Aneurysm. Clin Pract Cases Emerg Med, 2021. 5(3): p. 357-359. | Not POCUS |
| 342. | Seyedhosseini, J., et al., Association of optic nerve sheath diameter in ocular ultrasound with prognosis in patients presenting with acute stroke symptoms. Turk J Emerg Med, 2019. 19(4): p. 132-135. | Not in austere environment |
| 343. | Shafiee, H., et al., Paper and flexible substrates as materials for biosensing platforms to detect multiple biotargets. Sci Rep, 2015. 5: p. 8719. | Not POCUS |
| 344. | Shah, I., et al., Update on the management of craniomaxillofacial trauma in low-resource settings. Curr Opin Otolaryngol Head Neck Surg, 2019. 27(4): p. 274-279. | Not POCUS |
| 345. | Shahrestani, S., et al., A systematic review of next-generation point-of-care stroke diagnostic technologies. Neurosurg Focus, 2021. 51(1): p. E11. | Not POCUS |
| 346. | Sharma, A., et al., Efficacy of an online lung ultrasound module on skill acquisition by clinician: a new paradigm. Front Pediatr, 2024. 12: p. 1406630. | Curriculum development |
| 347. | Sharma, B., et al., Missed diagnosis of traumatic brain injury in patients with traumatic spinal cord injury. J Rehabil Med, 2014. 46(4): p. 370-3. | Not POCUS |
| 348. | Sharma, P., et al., Rationale & design of the PROMISES study: a prospective assessment and validation study of salivary progesterone as a test for preterm birth in pregnant women from rural India. Reprod Health, 2018. 15(1): p. 215. | Not POCUS |
| 349. | Sher, M., et al., Paper-based analytical devices for clinical diagnosis: recent advances in the fabrication techniques and sensing mechanisms. Expert Rev Mol Diagn, 2017. 17(4): p. 351-366. | Not POCUS |
| 350. | Shi, D., et al., Evaluation of a new goal-directed training curriculum for point-of-care ultrasound in the emergency department: impact on physician self-confidence and ultrasound skills. Eur J Trauma Emerg Surg, 2021. 47(2): p. 435-444. | Curriculum development |
| 351. | Shu, B., et al., A pocket-sized device automates multiplexed point-of-care RNA testing for rapid screening of infectious pathogens. Biosens Bioelectron, 2021. 181: p. 113145. | Not POCUS |
| 352. | Siddharthan, T., et al., Additive value of lung ultrasound to clinical parameters for prognosticating COVID-19. ERJ Open Res, 2023. 9(3). | Not in austere environment |
| 353. | Sieroszewski, P., J. Suzin, and E. Baś-Budecka, [Diagnostic methods for fetal malformations in the first half of pregnancy]. Ginekol Pol, 2003. 74(10): p. 1276-83. | Not POCUS |
| 354. | Simpson, E., et al., Gene Expression Alterations in Peripheral Blood Following Sport-Related Concussion in a Prospective Cohort of Collegiate Athletes: A Concussion Assessment, Research and Education (CARE) Consortium Study. Sports Med, 2024. 54(4): p. 1021-1032. | Not POCUS |
| 355. | Singh, I., S. Rohilla, and M. Kumawat, Twist drill aspiration of pyogenic brain abscesses: our experience in 103 cases. J Neurol Surg A Cent Eur Neurosurg, 2014. 75(3): p. 189-94. | Not POCUS |
| 356. | Singh, J., et al., Characteristics of Simulation-Based Point-of-Care Ultrasound Education: A Systematic Review of MedEdPORTAL Curricula. Cureus, 2022. 14(2): p. e22249. | Curriculum development |
| 357. | Smith, A., et al., Remote Mentoring of Point-of-Care Ultrasound Skills to Inexperienced Operators Using Multiple Telemedicine Platforms: Is a Cell Phone Good Enough? J Ultrasound Med, 2018. 37(11): p. 2517-2525. | No abstract |
| 358. | Snelling, P.J., et al., Bedside Ultrasound Conducted in Kids with distal upper Limb fractures in the Emergency Department (BUCKLED): a protocol for an open-label non-inferiority diagnostic randomised controlled trial. Trials, 2021. 22(1): p. 282. | Not in austere environment |
| 359. | Sodhi, K.S., et al., Imaging of thoracic tuberculosis in children: current and future directions. Pediatr Radiol, 2017. 47(10): p. 1260-1268. | Not POCUS |
| 360. | Sokunbi, O.J., et al., Maintaining paediatric cardiac services during the COVID-19 pandemic in a developing country in sub-Saharan Africa: guidelines for a "scale up" in the face of a global "scale down". Cardiol Young, 2020. 30(11): p. 1588-1594. | Not POCUS |
| 361. | Song, B., et al., Mobile-based oral cancer classification for point-of-care screening. J Biomed Opt, 2021. 26(6). | Not POCUS |
| 362. | Soták, M., T. Tyll, and K. Roubík, Temporary phrenic nerve stimulated patients: What is the role of ultrasound examination? Artif Organs, 2023. 47(3): p. 464-469. | Not POCUS |
| 363. | Srinivasan, B., et al., Point-of-Care Quantification of Serum Alpha-Fetoprotein for Screening Birth Defects in Resource-Limited Settings: Proof-of-Concept Study. JMIR Biomed Eng, 2021. 6(1). | Not POCUS |
| 364. | Stevens, D.Y., et al., Enabling a microfluidic immunoassay for the developing world by integration of on-card dry reagent storage. Lab Chip, 2008. 8(12): p. 2038-45. | Not POCUS |
| 365. | Stochholm, A. and B.B. Løgstrup, When you hear hoofbeats, think of horses but do not forget the zebras. BMJ Case Rep, 2015. 2015. | Not POCUS |
| 366. | Stolz, L.A., et al., Intussusception detected with ultrasound in a resource-limited setting. Lancet, 2013. 381(9882): p. 2054. | No abstract |
| 367. | Storti, E., A. Nailescu, and P.G. Villani, Lung Ultrasound in COVID-19 Critically Ill Patients with Acute Respiratory Distress Syndrome. J Cardiovasc Echogr, 2020. 30(Suppl 2): p. S11-s17. | Not in austere environment |
| 368. | Stybayeva, G., et al., Lensfree holographic imaging of antibody microarrays for high-throughput detection of leukocyte numbers and function. Anal Chem, 2010. 82(9): p. 3736-44. | Not POCUS |
| 369. | Su, T.W., et al., High-throughput lensfree imaging and characterization of a heterogeneous cell solution on a chip. Biotechnol Bioeng, 2009. 102(3): p. 856-868. | Not POCUS |
| 370. | Sun, H., et al., Safety of percutaneous vertebroplasty for the treatment of metastatic spinal tumors in patients with posterior wall defects. Eur Spine J, 2015. 24(8): p. 1768-77. | Not POCUS |
| 371. | Syverud, S.A., et al., Radiologic assessment of transvenous pacemaker placement during CPR. Ann Emerg Med, 1986. 15(2): p. 131-7. | Not POCUS |
| 372. | Tan, Z., et al., Retinopathy of prematurity screening: A narrative review of current programs, teleophthalmology, and diagnostic support systems. Saudi J Ophthalmol, 2022. 36(3): p. 283-295. | Not POCUS |
| 373. | Tavazzi, G., et al., Letter on "Pre-hospital transthoracic echocardiography for early identification of non-ST-elevation myocardial infarction in patients with acute coronary syndrome". Crit Care, 2018. 22(1): p. 311. | No abstract |
| 374. | Tazarourte, K., et al., Focused assessment with sonography in trauma prehospital triage: an important tool. Crit Care Med, 2010. 38(6): p. 1501-2; author reply 1502. | No abstract |
| 375. | Tazarourte, K., et al., Ultrasound and prehospital triage: a tool for limiting the undertriage. J Trauma, 2010. 69(4): p. 997. | No abstract |
| 376. | Terboven, T., et al., Chest wall thickness and depth to vital structures in paediatric patients - implications for prehospital needle decompression of tension pneumothorax. Scand J Trauma Resusc Emerg Med, 2019. 27(1): p. 45. | Not POCUS |
| 377. | Testa, A., et al., Economic analysis of bedside ultrasonography (US) implementation in an Internal Medicine department. Intern Emerg Med, 2015. 10(8): p. 1015-24. | Not involving healthcare workers |
| 378. | Thapa, A., Need of Integrating Sonoscopy in Undergraduate Medical Education in Developing Countries. J Nepal Health Res Counc, 2020. 18(3): p. 556-559. | Curriculum development |
| 379. | Theodoraki, K., et al., Ultrasonographic evaluation of abdominal organs after cardiac surgery. J Surg Res, 2015. 194(2): p. 351-360. | Not in austere environment |
| 380. | Thomas, D.M., et al., Effects of adenosine and regadenoson on hemodynamics measured using cardiovascular magnetic resonance imaging. J Cardiovasc Magn Reson, 2017. 19(1): p. 96. | Not POCUS |
| 381. | Thorpe, E., et al., The impact of clinical genome sequencing in a global population with suspected rare genetic disease. Am J Hum Genet, 2024. 111(7): p. 1271-1281. | Not POCUS |
| 382. | Tremblay, J.C., et al., UBC-Nepal expedition: upper and lower limb conduit artery shear stress and flow-mediated dilation on ascent to 5,050 m in lowlanders and Sherpa. Am J Physiol Heart Circ Physiol, 2018. 315(6): p. H1532-h1543. | Not POCUS |
| 383. | Tripu, R., et al., Graduating Surgical Residents Lack Competence in Critical Care Ultrasound. J Surg Educ, 2018. 75(3): p. 582-588. | Not POCUS |
| 384. | Tromp, J., et al., Nurse-led home-based detection of cardiac dysfunction by ultrasound: results of the CUMIN pilot study. Eur Heart J Digit Health, 2024. 5(2): p. 163-169. | Not in austere environment |
| 385. | Tung, D., et al., Evaluation of 1-(2-deoxy-2-fluoro-1-D-arabinofuranosyl)-5-iodouracil (FIAU) as an ex vivo bacterial detection agent. World J Microbiol Biotechnol, 2014. 30(11): p. 3003-10. | Not POCUS |
| 386. | Turc, G., et al., Comparison of Mobile Stroke Unit With Usual Care for Acute Ischemic Stroke Management: A Systematic Review and Meta-analysis. JAMA Neurol, 2022. 79(3): p. 281-290. | Not POCUS |
| 387. | Tuzun, H.Y., S. Turkkan, and A. Arsenishvili, The effectiveness of bedside point-of-care ultrasonography in the diagnosis and management of metacarpal fractures: Contribution for management of metacarpal fractures. Am J Emerg Med, 2016. 34(3): p. 674. | No abstract |
| 388. | Tuzun, H.Y., et al., Accuracy of bedside ultrasonography for the diagnosis of phalanx fractures. Am J Emerg Med, 2016. 34(8): p. 1698. | Not in austere environment |
| 389. | Uthoff, R.D., et al., Small form factor, flexible, dual-modality handheld probe for smartphone-based, point-of-care oral and oropharyngeal cancer screening. J Biomed Opt, 2019. 24(10): p. 1-8. | Not POCUS |
| 390. | Valaikiene, J., et al., Point-of-Care Ultrasound in Neurology - Report of the EAN SPN/ESNCH/ERcNsono Neuro-POCUS Working Group. Ultraschall Med, 2022. 43(4): p. 354-366. | Not in austere environment |
| 391. | Valdez, D., et al., Technical note: Low clinical efficacy, but good acceptability of a point-of-care electronic palpation device for breast cancer screening for a lower middle-income environment. Med Phys, 2022. 49(4): p. 2663-2671. | Not POCUS |
| 392. | Van den Bempt, S., L. Wauters, and P. Dewolf, Pulseless Electrical Activity: Detection of Underlying Causes in a Prehospital Setting. Med Princ Pract, 2021. 30(3): p. 212-222. | Not POCUS |
| 393. | van Gaal, S. and A. Demchuk, Clinical and Technological Approaches to the Prehospital Diagnosis of Large Vessel Occlusion. Stroke, 2018. 49(4): p. 1036-1043. | Not POCUS |
| 396. | van Turenhout, E.C., et al., Pre-hospital transfusion of red blood cells. Part 1: A scoping review of current practice and transfusion triggers. Transfus Med, 2020. 30(2): p. 86-105. | Not POCUS |
| 397. | Varrias, D., et al., The Use of Point-of-Care Ultrasound (POCUS) in the Diagnosis of Deep Vein Thrombosis. J Clin Med, 2021. 10(17). | Not in austere environment |
| 398. | Varsou, O., The Use of Ultrasound in Educational Settings: What Should We Consider When Implementing this Technique for Visualisation of Anatomical Structures? Adv Exp Med Biol, 2019. 1156: p. 1-11. | Not in austere environment |
| 399. | Vasios, W.N., 3rd, SOLCUS After-Action Report: From Good Idea to the Largest Ultrasound Training Program in the Department of Defense. J Spec Oper Med, 2016. 16(1): p. 62-5. | No abstract |
| 400. | Veldtman, G.R., et al., Management principles in patients with COVID-19: perspectives from a growing global experience with emphasis on cardiovascular surveillance. Open Heart, 2020. 7(2). | Not POCUS |
| 401. | Vemulapati, S., et al., A Quantitative Point-of-Need Assay for the Assessment of Vitamin D(3) Deficiency. Sci Rep, 2017. 7(1): p. 14142. | Not POCUS |
| 402. | Venkatesh, A.G., et al., Yeast dual-affinity biobricks: Progress towards renewable whole-cell biosensors. Biosens Bioelectron, 2015. 70: p. 462-8. | Not POCUS |
| 403. | Via, G., et al., Point of care ultrasound for sepsis management in resource-limited settings: time for a new paradigm for global health care. Intensive Care Med, 2012. 38(8): p. 1405-7; author reply 1408-9. | No abstract |
| 404. | Walsh, K.A. and R.M. Grivell, Use of endoanal ultrasound for reducing the risk of complications related to anal sphincter injury after vaginal birth. Cochrane Database Syst Rev, 2015. 2015(10): p. Cd010826. | Not in austere environment |
| 405. | Walter, S., I.Q. Grunwald, and K. Fassbender, [Mobile stroke unit for prehospital stroke treatment]. Radiologe, 2016. 56(1): p. 28-31. | Not POCUS |
| 406. | Walter, S., et al., Bringing the hospital to the patient: first treatment of stroke patients at the emergency site. PLoS One, 2010. 5(10): p. e13758. | Not POCUS |
| 407. | Wang, J.P., et al., [Effect of intermittent pneumatic compression on coagulation function and deep venous hemodynamics of lower limbs after rectal cancer resection]. Zhonghua Wei Chang Wai Ke Za Zhi, 2013. 16(8): p. 739-43. | Not POCUS |
| 408. | Wang, P., et al., [Clinical value of emergency bedside ultrasonocardiogram in cardiac care unit]. Di Yi Jun Yi Da Xue Xue Bao, 2002. 22(8): p. 762-3. | Not in austere environment |
| 409. | Wang, S., et al., Flexible Substrate-Based Devices for Point-of-Care Diagnostics. Trends Biotechnol, 2016. 34(11): p. 909-921. | Not POCUS |
| 410. | Wang, S., et al., Micro-a-fluidics ELISA for rapid CD4 cell count at the point-of-care. Sci Rep, 2014. 4: p. 3796. | Not POCUS |
| 411. | Wang, Y., et al., Development of a Naked Eye CRISPR-Cas12a and -Cas13a Multiplex Point-of-Care Detection of Genetically Modified Swine. ACS Synth Biol, 2023. 12(7): p. 2051-2060. | Not POCUS |
| 412. | Ward, D.I., Prehospital point-of-care ultrasound use by the military. Emerg Med Australas, 2007. 19(3): p. 282. | No abstract |
| 413. | Ward, Z.J., et al., The role and contribution of treatment and imaging modalities in global cervical cancer management: survival estimates from a simulation-based analysis. Lancet Oncol, 2020. 21(8): p. 1089-1098. | Not POCUS |
| 414. | Ward, Z.J., et al., Estimating the impact of treatment and imaging modalities on 5-year net survival of 11 cancers in 200 countries: a simulation-based analysis. Lancet Oncol, 2020. 21(8): p. 1077-1088. | Not POCUS |
| 415. | Washington, D.L., et al., Development of Quality Indicators for the Care of Women with Abnormal Uterine Bleeding by Primary Care Providers in the Veterans Health Administration. Womens Health Issues, 2019. 29(2): p. 135-143. | Not POCUS |
| 416. | Watchorn, J., et al., Decreased renal cortical perfusion, independent of changes in renal blood flow and sublingual microcirculatory impairment, is associated with the severity of acute kidney injury in patients with septic shock. Crit Care, 2022. 26(1): p. 261. | Not POCUS |
| 417. | Waterman, B., et al., Quality of abdominal ultrasound image acquisition by novice practitioners following a minimal training session on healthy volunteers. Cjem, 2020. 22(S2): p. S74-s78. | Not in austere environment |
| 418. | Waterman, B., et al., Abdominal ultrasound image acquisition and interpretation by novice practitioners after minimal training on a simulated patient model. Cjem, 2020. 22(S2): p. S62-s66. | Not in austere environment |
| 419. | Waydhas, C., et al., Prehospital management of chest injuries in severely injured patients-a systematic review and clinical practice guideline update. Eur J Trauma Emerg Surg, 2024. | Not POCUS |
| 420. | Weaver, J., et al., Delineating the relationship between Point A prescription dose and pelvic lymph node doses in intracavitary high-dose-rate brachytherapy treatment of cervical cancer for use in low- and middle-income countries. Brachytherapy, 2018. 17(1): p. 201-207. | Not POCUS |
| 421. | Weile, J., J. Brix, and A.B. Moellekaer, Is point-of-care ultrasound disruptive innovation? Formulating why POCUS is different from conventional comprehensive ultrasound. Crit Ultrasound J, 2018. 10(1): p. 25. | Curriculum development |
| 422. | Weimer, J.M., et al., Inter-System Variability of Eight Different Handheld Ultrasound (HHUS) Devices-A Prospective Comparison of B-Scan Quality and Clinical Significance in Intensive Care. Diagnostics (Basel), 2023. 14(1). | Not involving healthcare workers |
| 423. | Welsh, R.C., et al., A novel enoxaparin regime for ST elevation myocardial infarction patients undergoing primary percutaneous coronary intervention: a WEST sub-study. Catheter Cardiovasc Interv, 2007. 70(3): p. 341-8. | Not POCUS |
| 424. | Wheeler, R.T. and J.P. Kovacic, The use of a Foley balloon catheter to control junctional hemorrhage in a dog with severe vascular injury secondary to penetrating trauma. J Vet Emerg Crit Care (San Antonio), 2022. 32(1): p. 119-124. | Not POCUS |
| 425. | White, C.W., Benefit of aggressive lipid-lowering therapy: insights from the post coronary artery bypass graft study and other trials. Am J Med, 1998. 105(1a): p. 63s-68s. | Not POCUS |
| 426. | White-Dzuro, G.A., et al., Portable Handheld Point-of-Care Ultrasound for Detecting Unrecognized Esophageal Intubations. Respir Care, 2022. 67(5): p. 607-612. | Not in austere environment |
| 427. | Williams, J., et al., A Novel Low-Cost Simulation Model for Point-of-Care Ultrasound Intussusception Practice. Cureus, 2024. 16(5): p. e61016. | Not in austere environment |
| 428. | Wilson, L.A., et al., Postnatal gestational age estimation via newborn screening analysis: application and potential. Expert Rev Proteomics, 2019. 16(9): p. 727-731. | Not POCUS |
| 429. | Wu, S., et al., On-treatment changes of liver stiffness at week 26 could predict 2-year clinical outcomes in HBV-related compensated cirrhosis. Liver Int, 2018. 38(6): p. 1045-1054. | Not POCUS |
| 430. | Wu, T.F., et al., A Rapid and Low-Cost Pathogen Detection Platform by Using a Molecular Agglutination Assay. ACS Cent Sci, 2018. 4(11): p. 1485-1494. | Not POCUS |
| 431. | Xiong, F., et al., Arterial spin labeling magnetic resonance evaluates changes of cerebral blood flow in patients with mild traumatic brain injury. Zhong Nan Da Xue Xue Bao Yi Xue Ban, 2022. 47(8): p. 1016-1024. | Not POCUS |
| 432. | Xu, X., et al., Smartphone-Based Accurate Analysis of Retinal Vasculature towards Point-of-Care Diagnostics. Sci Rep, 2016. 6: p. 34603. | Not POCUS |
| 433. | Yan, F., et al., Tumor-penetrating Peptide-integrated Thermally Sensitive Liposomal Doxorubicin Enhances Efficacy of Radiofrequency Ablation in Liver Tumors. Radiology, 2017. 285(2): p. 462-471. | Not POCUS |
| 434. | Yildiz, C., et al., AO tension band technique application in proximal humerus fractures. Eklem Hastalik Cerrahisi, 2010. 21(2): p. 62-7. | Not POCUS |
| 435. | Yin, Z., et al., Ultrastable Plasmonic Bioink for Printable Point-Of-Care Biosensors. ACS Appl Mater Interfaces, 2020. 12(32): p. 35977-35985. | Not POCUS |
| 436. | You, D., et al., Automated image processing with point-of-care ocular ultrasound for real-time icp monitoring. Critical Care Medicine, 2022. 50(1 SUPPL): p. 274. | Not involving healthcare workers |
| 437. | Yue, Z., et al., Comparison of invisalign mandibular advancement and twin-block on upper airway and hyoid bone position improvements for skeletal class II children: a retrospective study. BMC Oral Health, 2023. 23(1): p. 661. | Not POCUS |
| 438. | Yuniar, I., et al., Vascular Reactivity Index and PELOD-2 as a mortality predictor in paediatric septic shock: a single-centre retrospective study. BMJ Paediatr Open, 2022. 6(1). | Not POCUS |
| 439. | Zhang, R.Y., et al., Three-dimensional morphological analysis of the femoral neck torsion angle-an anatomical study. J Orthop Surg Res, 2020. 15(1): p. 192. | Not POCUS |
| 440. | Zouridakis, G., et al., Melanoma and other skin lesion detection using smart handheld devices. Methods Mol Biol, 2015. 1256: p. 459-96. | Not POCUS |
| 441. | Zühlke, L. and B.M. Mayosi, Echocardiographic screening for subclinical rheumatic heart disease remains a research tool pending studies of impact on prognosis. Curr Cardiol Rep, 2013. 15(3): p. 343. | Not in austere environment |
| 442. | Zwank, M.D., et al., Improving CT scanner efficiency for trauma team activations in the emergency department. Am J Emerg Med, 2024. 75: p. 87-89. | Not POCUS |
