# Supplementary material for: Point-of-care ultrasound use in austere environments: A scoping review
Source: PLoS One. 2024 Dec 5;19(12):e0312017. doi: 10.1371/journal.pone.0312017 (PMC11620461; doi:10.1371/journal.pone.0312017)
Supplement: Attachment — Submitted filename: Reviewer Confidential Comments to Editor.doc [file pone.0312017.s008.doc]

Dear editor:

It's my great honor to be the reviewer of this magazine.

After thoroughly review, I found that although the novelty factor of the article is generally good, minor revision is indispensable.

With best regards,

Yours sincerely,

Xiang Ji
