# Supplementary material for: Point-of-care ultrasound use in austere environments: A scoping review
Source: PLoS One. 2024 Dec 5;19(12):e0312017. doi: 10.1371/journal.pone.0312017 (PMC11620461; doi:10.1371/journal.pone.0312017)
Supplement: Attachment — Submitted filename: Response to reviewers - PLOS ONE Austere US 22.9.24.docx [file pone.0312017.s009.docx]

PONE-D-24-38925

Point-of-care ultrasound use in austere environments: a scoping review

PLOS ONE

Dear Dr. Theophanous,

Thank you for submitting your manuscript to PLOS ONE. After careful consideration, we feel that it has merit but does not fully meet PLOS ONE’s publication criteria as it currently stands. Therefore, we invite you to submit a revised version of the manuscript that addresses the points raised during the review process.

Please submit your revised manuscript by Nov 04 2024 11:59PM. If you will need more time than this to complete your revisions, please reply to this message or contact the journal office at [plosone@plos.org](mailto:plosone@plos.org). When you're ready to submit your revision, log on to https://www.editorialmanager.com/pone/ and select the 'Submissions Needing Revision' folder to locate your manuscript file.

Please include the following items when submitting your revised manuscript:

- A rebuttal letter that responds to each point raised by the academic editor and reviewer(s). You should upload this letter as a separate file labeled 'Response to Reviewers'.
- A marked-up copy of your manuscript that highlights changes made to the original version. You should upload this as a separate file labeled 'Revised Manuscript with Track Changes'.
- An unmarked version of your revised paper without tracked changes. You should upload this as a separate file labeled 'Manuscript'.

If you would like to make changes to your financial disclosure, please include your updated statement in your cover letter. Guidelines for resubmitting your figure files are available below the reviewer comments at the end of this letter.

If applicable, we recommend that you deposit your laboratory protocols in protocols.io to enhance the reproducibility of your results. Protocols.io assigns your protocol its own identifier (DOI) so that it can be cited independently in the future. For instructions see: [https://journals.plos.org/plosone/s/submission-guidelines#loc-laboratory-protocols](https://nam11.safelinks.protection.outlook.com/?url=https%3A%2F%2Fc05y1x9s.r.us-east-2.awstrack.me%2FL0%2Fhttps%3A%252F%252Fjournals.plos.org%252Fplosone%252Fs%252Fsubmission-guidelines%2523loc-laboratory-protocols%2F1%2F010f01920fcdaf5b-7b2186f1-1e10-4240-9b69-1dc5a7a6a638-000000%2Fn3-jLJ4rvzq3Ww7iQTLer08JSG8%3D177&data=05%7C02%7Crebecca.theophanous%40duke.edu%7C1785d741909b41b02f6608dcd97f4b3a%7Ccb72c54e4a314d9eb14a1ea36dfac94c%7C0%7C0%7C638624391318759282%7CUnknown%7CTWFpbGZsb3d8eyJWIjoiMC4wLjAwMDAiLCJQIjoiV2luMzIiLCJBTiI6Ik1haWwiLCJXVCI6Mn0%3D%7C0%7C%7C%7C&sdata=07HIGL0QDy%2F%2BImQsPB74kYIU2GhHDUzoDmqStzI7Buc%3D&reserved=0). Additionally, PLOS ONE offers an option for publishing peer-reviewed Lab Protocol articles, which describe protocols hosted on protocols.io. Read more information on sharing protocols at [https://plos.org/protocols?utm_medium=editorial-email&utm_source=authorletters&utm_campaign=protocols](https://nam11.safelinks.protection.outlook.com/?url=https%3A%2F%2Fc05y1x9s.r.us-east-2.awstrack.me%2FL0%2Fhttps%3A%252F%252Fplos.org%252Fprotocols%253Futm_medium%3Deditorial-email%2526utm_source%3Dauthorletters%2526utm_campaign%3Dprotocols%2F1%2F010f01920fcdaf5b-7b2186f1-1e10-4240-9b69-1dc5a7a6a638-000000%2FZci0Y8Iv3AoDfcbgWoACK2CJvAA%3D177&data=05%7C02%7Crebecca.theophanous%40duke.edu%7C1785d741909b41b02f6608dcd97f4b3a%7Ccb72c54e4a314d9eb14a1ea36dfac94c%7C0%7C0%7C638624391318783845%7CUnknown%7CTWFpbGZsb3d8eyJWIjoiMC4wLjAwMDAiLCJQIjoiV2luMzIiLCJBTiI6Ik1haWwiLCJXVCI6Mn0%3D%7C0%7C%7C%7C&sdata=NshX8qa9mBX2pMOvf5E3P1QZ4N%2B65CmPv3rJQOQFDnY%3D&reserved=0).

We look forward to receiving your revised manuscript.

Kind regards,

Juan Antonio Valera-Calero
Academic Editor
PLOS ONE

Journal requirements:

When submitting your revision, we need you to address these additional requirements.

1. Please ensure that your manuscript meets PLOS ONE's style requirements, including those for file naming. The PLOS ONE style templates can be found at

<https://journals.plos.org/plosone/s/file?id=wjVg/PLOSOne_formatting_sample_main_body.pdf> and

[https://journals.plos.org/plosone/s/file?id=ba62/PLOSOne_formatting_sample_title_authors_affiliations.pdf](https://nam11.safelinks.protection.outlook.com/?url=https%3A%2F%2Fc05y1x9s.r.us-east-2.awstrack.me%2FL0%2Fhttps%3A%252F%252Fjournals.plos.org%252Fplosone%252Fs%252Ffile%253Fid%3Dba62%252FPLOSOne_formatting_sample_title_authors_affiliations.pdf%2F1%2F010f01920fcdaf5b-7b2186f1-1e10-4240-9b69-1dc5a7a6a638-000000%2F1Gno_bX6sbhgdcsszqYinEE0X9o%3D177&data=05%7C02%7Crebecca.theophanous%40duke.edu%7C1785d741909b41b02f6608dcd97f4b3a%7Ccb72c54e4a314d9eb14a1ea36dfac94c%7C0%7C0%7C638624391318798395%7CUnknown%7CTWFpbGZsb3d8eyJWIjoiMC4wLjAwMDAiLCJQIjoiV2luMzIiLCJBTiI6Ik1haWwiLCJXVCI6Mn0%3D%7C0%7C%7C%7C&sdata=7lKDAFJxw232hyxsOTgz0eXDster2fm26H78vHp8HXQ%3D&reserved=0) - manuscript and cover/title pages were reformatted per the PLOS ONE journal guidelines

2. As required by our policy on Data Availability, please ensure your manuscript or supplementary information includes the following:

A numbered table of all studies identified in the literature search, including those that were excluded from the analyses. -added numbered tables of included and excluded studies as S5 and S6, also the included studies were already detailed in S3 Austere US included studies

For every excluded study, the table should list the reason(s) for exclusion. -exclusion reasons are listed in S6 and also added to Fig1 study flow diagram

If any of the included studies are unpublished, include a link (URL) to the primary source or detailed information about how the content can be accessed. N/A

A table of all data extracted from the primary research sources for the systematic review and/or meta-analysis. The table must include the following information for each study: - all included study details are in S3 Table of included studies. Added text answering the below questions.

Name of data extractors and date of data extraction

Confirmation that the study was eligible to be included in the review.

All data extracted from each study for the reported systematic review and/or meta-analysis that would be needed to replicate your analyses.

If data or supporting information were obtained from another source (e.g. correspondence with the author of the original research article), please provide the source of data and dates on which the data/information were obtained by your research group. – added text about emails to study authors in the methods section

If applicable for your analysis, a table showing the completed risk of bias and quality/certainty assessments for each study or outcome.  Please ensure this is provided for each domain or parameter assessed. For example, if you used the Cochrane risk-of-bias tool for randomized trials, provide answers to each of the signalling questions for each study. If you used GRADE to assess certainty of evidence, provide judgements about each of the quality of evidence factor. This should be provided for each outcome.  – GRADE assessments are explained both within the manuscript and for each individual study in S3 Table of all included studies

An explanation of how missing data were handled. – added text in the methods and in Table 2 as not reported by the study authors (NR)

This information can be included in the main text, supplementary information, or relevant data repository. Please note that providing these underlying data is a requirement for publication in this journal, and if these data are not provided your manuscript might be rejected.

3. Please review your reference list to ensure that it is complete and correct. If you have cited papers that have been retracted, please include the rationale for doing so in the manuscript text, or remove these references and replace them with relevant current references. Any changes to the reference list should be mentioned in the rebuttal letter that accompanies your revised manuscript. If you need to cite a retracted article, indicate the article’s retracted status in the References list and also include a citation and full reference for the retraction notice. – reference list was updated with standardized formatting. No retracted papers were cited.

Additional Editor Comments (if provided):
[Note: HTML markup is below. Please do not edit.]

Reviewers' comments:

Reviewer's Responses to Questions

**Comments to the Author**

1. Is the manuscript technically sound, and do the data support the conclusions?

The manuscript must describe a technically sound piece of scientific research with data that supports the conclusions. Experiments must have been conducted rigorously, with appropriate controls, replication, and sample sizes. The conclusions must be drawn appropriately based on the data presented.

Reviewer #1: Yes

Reviewer #2: Yes

Reviewer #3: Yes

2. Has the statistical analysis been performed appropriately and rigorously?

Reviewer #1: Yes

Reviewer #2: Yes

Reviewer #3: Yes

3. Have the authors made all data underlying the findings in their manuscript fully available?

The [PLOS Data policy](https://nam11.safelinks.protection.outlook.com/?url=http%3A%2F%2Fc05y1x9s.r.us-east-2.awstrack.me%2FL0%2Fhttp%3A%252F%252Fwww.plosone.org%252Fstatic%252Fpolicies.action%2523sharing%2F1%2F010f01920fcdaf5b-7b2186f1-1e10-4240-9b69-1dc5a7a6a638-000000%2FVxQn-RQUmD_4G9jLx9anfigyzcc%3D177&data=05%7C02%7Crebecca.theophanous%40duke.edu%7C1785d741909b41b02f6608dcd97f4b3a%7Ccb72c54e4a314d9eb14a1ea36dfac94c%7C0%7C0%7C638624391318811799%7CUnknown%7CTWFpbGZsb3d8eyJWIjoiMC4wLjAwMDAiLCJQIjoiV2luMzIiLCJBTiI6Ik1haWwiLCJXVCI6Mn0%3D%7C0%7C%7C%7C&sdata=%2Be22O5msZrjEGflU%2BXtH0o9jLmZeE9oS%2B27I579gFGk%3D&reserved=0) requires authors to make all data underlying the findings described in their manuscript fully available without restriction, with rare exception (please refer to the Data Availability Statement in the manuscript PDF file). The data should be provided as part of the manuscript or its supporting information, or deposited to a public repository. For example, in addition to summary statistics, the data points behind means, medians and variance measures should be available. If there are restrictions on publicly sharing data—e.g. participant privacy or use of data from a third party—those must be specified.

Reviewer #1: Yes

Reviewer #2: Yes

Reviewer #3: Yes

4. Is the manuscript presented in an intelligible fashion and written in standard English?

PLOS ONE does not copyedit accepted manuscripts, so the language in submitted articles must be clear, correct, and unambiguous. Any typographical or grammatical errors should be corrected at revision, so please note any specific errors here.

Reviewer #1: Yes

Reviewer #2: Yes

Reviewer #3: Yes

5. Review Comments to the Author

Please use the space provided to explain your answers to the questions above. You may also include additional comments for the author, including concerns about dual publication, research ethics, or publication ethics. (Please upload your review as an attachment if it exceeds 20,000 characters)

Reviewer #1: Review Comments to the Author:
1.The manuscript presents a well-organized scoping review on the use of point-of-care ultrasound (POCUS) in austere environments. The methodology follows the PRISMA-ScR guidelines, and the GRADE framework is used to assess the quality of the included studies. However, I recommend changing the category of the manuscript from "Research Article" to "Review Article" to reflect its nature more accurately. – changed to “review article”

2. Statistical Analysis
Since this is a scoping review, statistical analysis is not the primary focus. The authors use descriptive statistics to summarize the number and types of studies included in the review, as well as their quality, as assessed by the GRADE tool. This approach is suitable for a review, but the manuscript does not include any advanced statistical analyses. Consider incorporating more advanced statistical tools where applicable to strengthen the findings. – We followed the PRISMA-ScR guidelines, which use descriptive statistics and basic qualitative content analysis. The methods section was expanded to better explain the authors’ process. Hopefully this is more transparent for the reviewers and editors.

3. Data Availability
The authors have made all relevant data underlying the manuscript's findings available without restriction, in accordance with PLOS ONE's data policy. This is clearly stated in the Data Availability Statement. As this is a review article, the data consist of extracted information from previously published studies, which are fully described in the manuscript and supporting information. The authors have met the journal's requirements by ensuring transparency in data sharing.

4. Language and Clarity
The manuscript is generally well-written and adheres to the standards of academic English. The scientific content is clear, but minor revisions could improve readability and flow. Specifically, some sentences are quite long and could be split into shorter, more concise statements. Additionally, there are a few grammatical issues and inconsistencies in the use of abbreviations that need to be addressed for better clarity. A thorough review for grammar and sentence structure would be beneficial. – grammar was corrected, abbreviations changed to parentheses after the first mention, and sentences shortened

5. General Comments
(A) This manuscript makes a valuable contribution to the understanding of POCUS applications in austere environments. It emphasizes the potential of portable ultrasound devices in resource-limited settings and other challenging environments. The use of the GRADE assessment tool adds rigor by providing an objective measure of the quality of the studies included, though it also highlights the need for more robust, high-quality research in this field.

(B) There are no concerns regarding dual publication, research ethics, or publication ethics. As this is a review article and does not involve new experimental research, participant consent and privacy issues are not applicable. The authors have declared no competing interests, and no specific funding was received, ensuring transparency.

(C) Other Recommendations for Revision:
1. Clarify and shorten complex sentences to improve readability. -corrected
2. Ensure consistent use of abbreviations after their introduction. -corrected
3. Correct minor grammatical and typographical errors noted during proofreading. -corrected
4. Expand the discussion on future research directions, particularly in relation to the use of AI and telemedicine in POCUS applications. – added more details to teleultrasound and AI explanations in the future directions section
5. Consider using more statistical analysis tools where appropriate to provide deeper insights. – see above comments

Reviewer #2: This manuscript by Theophanous RG et al. entitled “Point-of-care ultrasound（POCUS） use in austere environments: a scoping review” aims to explore the use of POCUS in a variety of Settings, including military and conflict areas, prehospital, RLS including LMIC, outer space, and high altitudes. The authors provide an interesting and potentially important manuscript that adequately describes current advances in the POCUS, as well as potential future directions. But there are still some content need to explain further.

Major：
1. What’s the difference between “point-of-care ultrasound” and “bedside ultrasound”? What is the reason for this paper to choose POCUS for in-depth study? – POCUS and bedside US are the same. POCUS is a more well-known and accepted term, thus is used throughout the manuscript. The reason for the scoping review for POCUS in austere environments is further explained in the methods section. A study aim was added at the end of the introduction section.
2. Because POCUS models and manufacturers are different, so the image display and application are different. Is there any relevant standard or classification basis in the process of literature collection? If you can add improved methods to the manuscript, it will be of great value to those who read the paper. -the methods section was expanded and details added regarding the PCC framework and other methodology to make this transparent for the reviewers/editors.
3. There were some minor issues with the references, including incomplete citations or outdated sources. Please check the references carefully and make the necessary corrections. – references were updated and standardized

Reviewer #3: International work! The manuscript entitled "Point-of-care ultrasound use in austere environments: a scoping review” updated narrative scoping review describes POCUS use in these global settings. The novelty factor of the article is good. I find the article potentially acceptable for publication after minor revision, my comments are below:

1. Please standardize the font, including the documents in the supplementary materials. -font was standardized to Arial size 11 across all documents

2. Please add tables or figures to summarize each setting. – S3 Table 1a-f and S4 Table 2 summarize all included studies by each study setting. Table 2 synthesizes the data from all study categories and compares POCUS examinations by study setting.

3. Please unify the tables into three-line tables. – Tables were all reformatted into three-line tables

6. PLOS authors have the option to publish the peer review history of their article ([what does this mean?](https://nam11.safelinks.protection.outlook.com/?url=https%3A%2F%2Fc05y1x9s.r.us-east-2.awstrack.me%2FL0%2Fhttps%3A%252F%252Fjournals.plos.org%252Fplosone%252Fs%252Feditorial-and-peer-review-process%2523loc-peer-review-history%2F1%2F010f01920fcdaf5b-7b2186f1-1e10-4240-9b69-1dc5a7a6a638-000000%2FDbtg4phTike0Jv2JNOhb6JEajDQ%3D177&data=05%7C02%7Crebecca.theophanous%40duke.edu%7C1785d741909b41b02f6608dcd97f4b3a%7Ccb72c54e4a314d9eb14a1ea36dfac94c%7C0%7C0%7C638624391318824928%7CUnknown%7CTWFpbGZsb3d8eyJWIjoiMC4wLjAwMDAiLCJQIjoiV2luMzIiLCJBTiI6Ik1haWwiLCJXVCI6Mn0%3D%7C0%7C%7C%7C&sdata=5qC2EAv6I%2Bvl31BiVarDXfQ2lSOVFY7VQ%2BnahNtBX4g%3D&reserved=0)). If published, this will include your full peer review and any attached files.

If you choose “no”, your identity will remain anonymous but your review may still be made public.

**Do you want your identity to be public for this peer review?** For information about this choice, including consent withdrawal, please see our [Privacy Policy](https://nam11.safelinks.protection.outlook.com/?url=https%3A%2F%2Fc05y1x9s.r.us-east-2.awstrack.me%2FL0%2Fhttps%3A%252F%252Fwww.plos.org%252Fprivacy-policy%2F1%2F010f01920fcdaf5b-7b2186f1-1e10-4240-9b69-1dc5a7a6a638-000000%2FO_VmEJmHlZjNacZm35_GR3WGTM8%3D177&data=05%7C02%7Crebecca.theophanous%40duke.edu%7C1785d741909b41b02f6608dcd97f4b3a%7Ccb72c54e4a314d9eb14a1ea36dfac94c%7C0%7C0%7C638624391318838007%7CUnknown%7CTWFpbGZsb3d8eyJWIjoiMC4wLjAwMDAiLCJQIjoiV2luMzIiLCJBTiI6Ik1haWwiLCJXVCI6Mn0%3D%7C0%7C%7C%7C&sdata=wfzN34DBGc2M6s6y9x2S47RLCgXdaGnbVg0VziWpRE8%3D&reserved=0).

Reviewer #1: No

Reviewer #2: No

Reviewer #3: No

[NOTE: If reviewer comments were submitted as an attachment file, they will be attached to this email and accessible via the submission site. Please log into your account, locate the manuscript record, and check for the action link "View Attachments". If this link does not appear, there are no attachment files.]

While revising your submission, please upload your figure files to the Preflight Analysis and Conversion Engine (PACE) digital diagnostic tool, [https://pacev2.apexcovantage.com/](https://nam11.safelinks.protection.outlook.com/?url=https%3A%2F%2Fc05y1x9s.r.us-east-2.awstrack.me%2FL0%2Fhttps%3A%252F%252Fpacev2.apexcovantage.com%252F%2F1%2F010f01920fcdaf5b-7b2186f1-1e10-4240-9b69-1dc5a7a6a638-000000%2FRZgR1uwJ-TF9FOTCbnjkhuB4aN4%3D177&data=05%7C02%7Crebecca.theophanous%40duke.edu%7C1785d741909b41b02f6608dcd97f4b3a%7Ccb72c54e4a314d9eb14a1ea36dfac94c%7C0%7C0%7C638624391318850734%7CUnknown%7CTWFpbGZsb3d8eyJWIjoiMC4wLjAwMDAiLCJQIjoiV2luMzIiLCJBTiI6Ik1haWwiLCJXVCI6Mn0%3D%7C0%7C%7C%7C&sdata=fC6QyH6jeXvTuo67uDsH0MKoFIOA%2BuV%2Bmi69zZKlDOo%3D&reserved=0). PACE helps ensure that figures meet PLOS requirements. To use PACE, you must first register as a user. Registration is free. Then, login and navigate to the UPLOAD tab, where you will find detailed instructions on how to use the tool. If you encounter any issues or have any questions when using PACE, please email PLOS at [figures@plos.org](mailto:figures@plos.org). Please note that Supporting Information files do not need this step.

*In compliance with data protection regulations, you may request that we remove your personal registration details at any time.* [*(Remove my information/details)*](https://nam11.safelinks.protection.outlook.com/?url=https%3A%2F%2Fc05y1x9s.r.us-east-2.awstrack.me%2FL0%2Fhttps%3A%252F%252Fwww.editorialmanager.com%252Fpone%252Flogin.asp%253Fa%3Dr%2F1%2F010f01920fcdaf5b-7b2186f1-1e10-4240-9b69-1dc5a7a6a638-000000%2F8XaqKEZwgBkJd9FIHoc4UtPjpGU%3D177&data=05%7C02%7Crebecca.theophanous%40duke.edu%7C1785d741909b41b02f6608dcd97f4b3a%7Ccb72c54e4a314d9eb14a1ea36dfac94c%7C0%7C0%7C638624391318867349%7CUnknown%7CTWFpbGZsb3d8eyJWIjoiMC4wLjAwMDAiLCJQIjoiV2luMzIiLCJBTiI6Ik1haWwiLCJXVCI6Mn0%3D%7C0%7C%7C%7C&sdata=FGkUm5FgmC81GVgPrARzYL8t6VReJ2woI7zNwEH%2F66c%3D&reserved=0)*. Please contact the publication office if you have any questions.*
