# Supplementary material for: Point-of-care ultrasound use in austere environments: A scoping review
Source: PLoS One. 2024 Dec 5;19(12):e0312017. doi: 10.1371/journal.pone.0312017 (PMC11620461; doi:10.1371/journal.pone.0312017)
Supplement: Attachment — Submitted filename: Specific comments to the authors.doc [file pone.0312017.s010.doc]

The manuscript entitled "Point-of-care ultrasound use in austere environments: a scoping review” updated narrative scoping review describes POCUS use in these global settings. The paper was well structured, and the novelty factor of the paper was generally good. The authors have well completed the revision. In my opinion, this paper is suitable to be published in PLOS ONE.
