# Supplementary material for: Point-of-care ultrasound use in austere environments: A scoping review
Source: PLoS One. 2024 Dec 5;19(12):e0312017. doi: 10.1371/journal.pone.0312017 (PMC11620461; doi:10.1371/journal.pone.0312017)
Supplement: Attachment — Submitted filename: Confidential Comments to the Editor.doc [file pone.0312017.s011.doc]

Dear editor:

It's my great honor to be the reviewer of this magazine.

The paper was well structured, and the novelty factor of the paper was generally good. In my opinion, this paper is suitable to be published in PLOS ONE.

With best regards,

Yours sincerely,

Xiang Ji
