# Supplementary material for: Point-of-care ultrasound use in austere environments: A scoping review
Source: PLoS One. 2024 Dec 5;19(12):e0312017. doi: 10.1371/journal.pone.0312017 (PMC11620461; doi:10.1371/journal.pone.0312017)
Supplement: Attachment — Submitted filename: Reviewer Comments to Author.doc [file pone.0312017.s007.doc]

International work! The manuscript entitled "Point-of-care ultrasound use in austere environments: a scoping review” updated narrative scoping review describes POCUS use in these global settings. The novelty factor of the article is good. I find the article potentially acceptable for publication after minor revision, my comments are below:

1. Please standardize the font, including the documents in the supplementary materials.
2. Please add tables or figures to summarize each setting.
3. Please unify the tables into three-line tables.
